# C-X-C motif chemokine ligand 1 derived from oral squamous cell carcinoma promotes cancer-associated fibroblast differentiation and tumor growth

**DOI:** 10.1186/s43556-025-00281-8

**Published:** 2025-06-10

**Authors:** Soon Chul Heo, In-hye Nam, Bo Ram Keum, Yeo Gyun Yun, Jae-Yeol Lee, Hyung Joon Kim

**Affiliations:** 1 Department of Oral Physiology, Periodontal Diseases Signaling Network Research Center, Dental and Life Science Institute, School of Dentistry, Pusan National University, Yangsan, 50612 Republic of Korea; 2 Institute of Tissue Regeneration Engineering (ITREN), Mechanobiology Dental Medicine Research Center, Dankook University, Cheonan, 31116 Republic of Korea; 3 Department of Oral and Maxillofacial Surgery, Dental Research Institute, and Dental and Life Science Institute, Pusan National University, School of Dentistry, Yangsan, 50612 Republic of Korea; 4 Institute of Tissue Regeneration Engineering (ITREN), Department of Nanobiomedical Science and BK21 Four NBM Global Research Center for Regenerative Medicine, Dankook University, Cheonan, 31116 Republic of Korea

**Keywords:** Oral squamous cell carcinoma, Cancer-associated fibroblasts, C-X-C motif chemokine ligand 1, C-X-C motif chemokine receptor 2

## Abstract

Cancer-associated fibroblasts (CAFs), the predominant stromal cells in the tumor microenvironment (TME), play a critical role in the progression of solid tumors, including oral squamous cell carcinoma (OSCC). However, the molecular mechanisms by which OSCC-derived factors mediate CAF differentiation remain incompletely understood. This study investigates the role of the C-X-C motif chemokine ligand 1 (CXCL1), secreted by OSCC cells, in promoting CAF differentiation and its downstream impact on tumor progression. Gingival fibroblasts (GFs) were treated with conditioned medium (CM) from various OSCC cell lines to assess their potential to induce CAF differentiation. Proteomic analysis using liquid chromatography–mass spectrometry identified CXCL1 as a key factor highly secreted in SCC25-derived CM, which exhibited the strongest capacity to induce CAF differentiation. CXCL1 synergistically enhanced TGF-β1-induced differentiation of GFs into α-smooth muscle actin (αSMA)- and vimentin-expressing CAFs by approximately 1.5-fold, confirming its co-stimulatory function. Conversely, silencing its receptor CXCR2 reduced CAF marker expression by over 50%, indicating a strong inhibitory effect on CAF differentiation. In vivo, co-injection of SCC25 cells with GFs significantly promoted tumor growth and stromal CAF marker expression, whereas CXCR2 knockdown in GFs led to a ~ 40% reduction in tumor volume and reduced αSMA/vimentin-positive CAFs. These findings establish CXCL1 as a pivotal mediator of CAF differentiation through CXCR2-dependent signaling, and highlight that the CXCL1–CXCR2 axis is a promising therapeutic target for modulating stromal–tumor interactions in OSCC.

**Supplementary Information:**

The online version contains supplementary material available at 10.1186/s43556-025-00281-8.

## Introduction

Oral cancer is the sixth most common cancer worldwide, with oral squamous cell carcinoma (OSCC) accounting for 90% of all oral cancer cases [1, 2]. Recent epidemiological studies indicate that OSCC has a recurrence rate of around 26% after primary curative therapy, and notably, more than 80% of these cases reappear within the initial two-year period [3]. Despite advancements in early diagnosis and treatment, the five-year survival rate of patients with OSCC remains low because of distant metastasis and recurrence, which greatly affects patients’ quality of life [4]. A more comprehensive understanding of the complex pathogenesis of OSCC is required to develop effective treatments.

The complexity of tumors arises from their multicellular nature, in which cancer cells interact dynamically with the tumor microenvironment (TME). The TME is crucial for tumor development, progression, and metastasis and comprises the extracellular matrix and various noncancerous cell types, including immune cells, endothelial cells, pericytes, and fibroblasts [5]. Cancer-associated fibroblasts (CAFs) are a varied and predominant stromal cell type that strongly influence the microenvironment of solid tumors. CAFs contribute to tumor progression and metastasis by influencing the TME and enhancing malignant cell proliferation [6, 7]. The presence and function of activated CAFs within the microenvironment correlate with an unfavorable prognosis in multiple cancers [8]. In addition, high levels of stromal signatures in tumors are associated with a reduced effectiveness of therapy and a higher incidence of disease recurrence [9]. Thus, studies of CAFs are needed to gain comprehensive insights into cancer initiation, progression, and therapeutic strategies.

Despite being a heterogeneous and poorly characterized cell population, CAFs, particularly myofibroblastic CAFs, have been the focus of several studies. Although immunohistochemistry of α-smooth muscle actin (αSMA, encoded by *ACTA2*) is frequently employed to identify these cells in tissue sections, this method is not specific to CAFs, as it is also expressed by pericytes and smooth muscle cells; other markers including fibroblast-specific protein, fibroblast activation protein, and vimentin have become commonly used to distinguish CAFs [10, 11]. These contractile cells, which are αSMA-positive, are mainly generated via mechanotransduction and transforming growth factor (TGF)-β signaling [12].

C-X-C motif chemokine ligand 1 (CXCL1), a member of the CXC chemokine family, is controlled by multiple signaling cues, including those from the tumor microenvironment. Numerous studies reported that CXCL1 is highly expressed in most OSCC cell lines, linking it to OSCC invasion and metastasis [13, 14]. CAF-derived CXCL1 contributes significantly to inducing dormancy in OSCC cells and simultaneously facilitates oral cancer cell motility and invasiveness, which is closely linked to unfavorable clinical outcomes, especially in cases of tongue carcinoma [15, 16]. Through its interaction with CXCL1, CXCR2, a receptor common to CXC chemokines, drives the progression of malignant carcinoma and promotes proliferation, migration, angiogenesis, and resistance to therapy [17]. In addition, circulating CXCL1 levels are higher in patients with metastasis than in those with stage IA-IIB non-small cell lung cancer [18]. Blocking CXCR2 significantly increased the response to chemotherapy and reduced tumor growth, angiogenesis, and metastasis [19, 20]. However, the specific role of CXCL1-CXCR2 interactions in OSCC, focusing on CAF differentiation, remains unclear.

Understanding the molecular mechanisms driving CAF differentiation in the TME is critical for developing new therapeutic strategies and improving the clinical outcomes of patients with OSCC. Emerging evidence suggests that chemokine-mediated stromal activation, particularly through the CXCL1–CXCR2 axis, may play a pivotal role in shaping the pro-tumorigenic TME; however, its functional contribution in OSCC remains unclear. This study aims to investigate the role of CXCL1 secreted by OSCC cells in promoting CAF differentiation via CXCR2-dependent signaling, and to evaluate how these activated CAFs contribute to OSCC progression through paracrine mechanisms. By elucidating these mechanisms, we aim to identify potential therapeutic targets, such as the CXCL1-CXCR2 interaction, for suppressing OSCC progression.

## Results

### Paracrine stimulation of gingival fibroblasts (GFs) by OSCC promotes CAF differentiation and accelerates OSCC proliferation, migration, and invasion

**Fig. 1:**
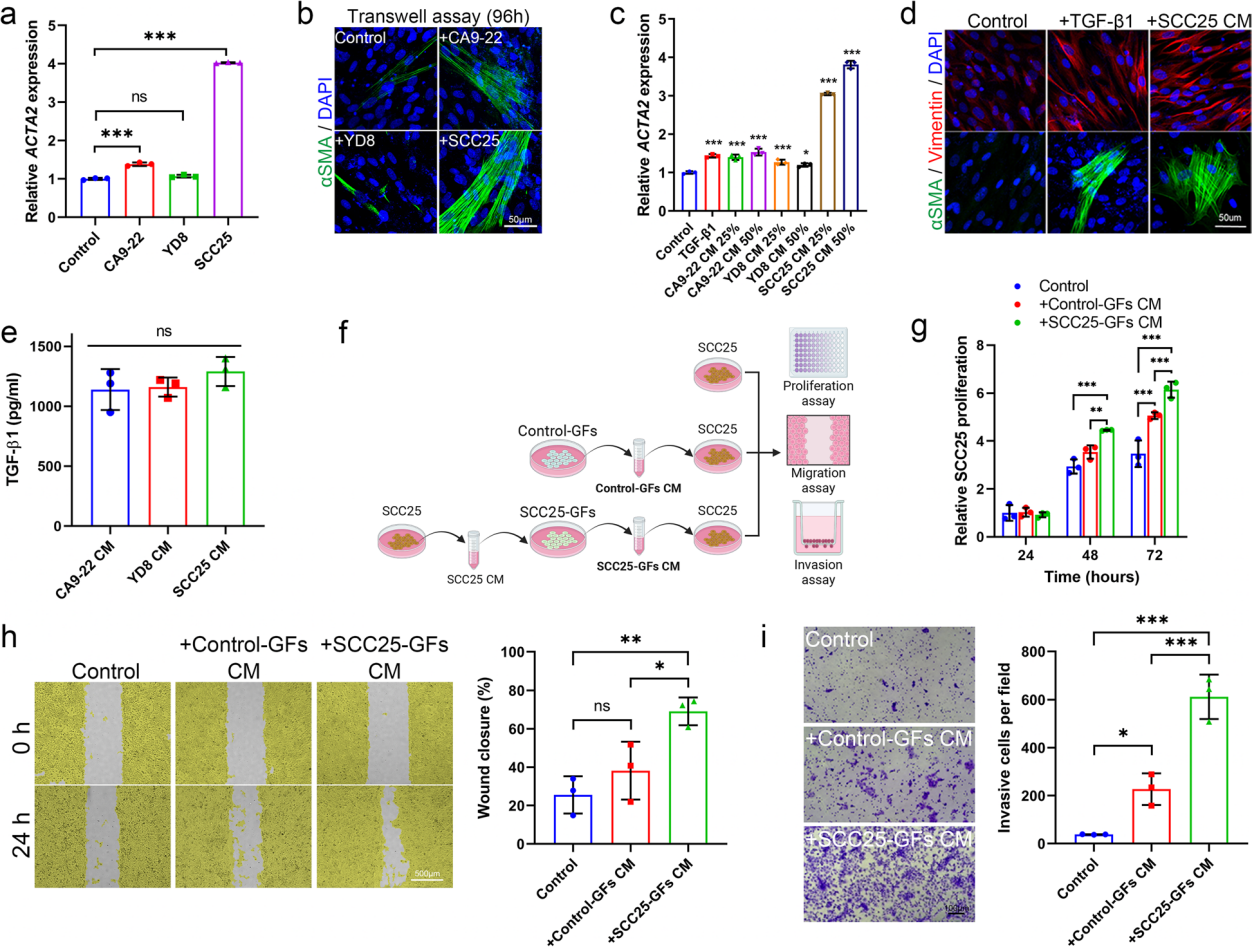
Paracrine stimulation of oral squamous cell carcinoma (OSCC) induces cancer-associated fibroblast (CAF) differentiation in gingival fibroblasts (GFs) and accelerates OSCC proliferation, migration and invasion. **a** *ACTA2* mRNA expression in GFs cultured in the lower chamber of a transwell system, with OSCC cell lines in the upper chamber for 96 h. **b** αSMA expression (green) in GFs cultured using the transwell system was detected by immunocytochemistry. Nuclei were counterstained with DAPI (blue). Scale bar = 50 μm. **c** GFs were incubated with 25% or 50% OSCC conditioned medium (CM) for 96 h. TGF-β1 (10 ng/mL) was used as positive control for CAF differentiation. mRNA expression of *ACTA2* was analyzed using qRT-PCR. **d** GFs were treated with TGF-β1 or SCC25 CM, and the expression of vimentin (red) and αSMA (green) was detected using immunocytochemistry. Nuclei were counterstained with DAPI (blue). Scale bar = 50 μm. **e** Secreted TGF-β1 proteins in OSCC CM were measured using ELISA. **f** Schematic illustration of the experimental procedure for CM-based assays, including proliferation, migration, and invasion experiments in SCC25 cells using CM derived from either control GFs or SCC25 CM-stimulated GFs. Illustration was created with BioRender.com. **g** SCC25 cell proliferation was analyzed using the cell counting kit-8 assay. **h** Migration of SCC25 cells was analyzed by scratch wound healing assay. Yellow-highlighted areas represent regions covered by cells. Scale bar = 500 μm. **i** SCC25 cell invasion was evaluated using transwell inserts. Scale bar = 100 μm. ^*^p < 0.05, ^**^p < 0.01, ^***^p < 0.001. DAPI, 4′,6-diamidino-2-phenylindole; ns, not significant

To investigate whether OSCC can stimulate CAF differentiation through a paracrine mechanism, we exposed GFs, the predominant cells in gingival tissues, to conditioned medium (CM) derived from OSCC cell lines for 96 h. Additionally, we used transwell assays to ensure that the observed CAF differentiation was not due to direct cell–cell contact. In the transwell system, GFs treated with OSCC CM showed significantly upregulated *ACTA2* mRNA expression, a primary marker of CAFs, as measured by qRT-PCR (Fig. 1a). Although the differences in *ACTA2* expression, particularly between the control and CA9-22 groups, appeared modest, they were statistically significant owing to the consistent and reproducible transcriptional responses observed across independent biological replicates. The minimal intra-group variability observed in these assays underscores the methodological rigor and analytical sensitivity of our experimental system, which enables the reliable detection of even subtle changes in gene expression. This transcript-level observation was supported by immunocytochemical analysis, which showed increased αSMA expression in GFs within the transwell culture system (Fig. 1b). While all tested OSCC CMs promoted *ACTA2* expression to varying degrees, SCC25 CM induced the most prominent upregulation (Fig. 1c). Consistently, treatment with SCC25 CM markedly enhanced αSMA and vimentin protein expression in GFs, comparable to the effect of TGF-β1, a canonical inducer of CAF differentiation (Fig. 1d). Interestingly, ELISA analysis revealed no significant differences in TGF-β1 protein levels across the OSCC cell lines (Fig. 1e), suggesting that additional factors beyond TGF-β1 are present in OSCC CM to promote CAF differentiation. To evaluate the functional impact of these induced CAFs on OSCC cells, we collected CM from either control GFs or SCC25 CM-treated GFs and applied it to SCC25 cells (Fig. 1f). Treatment with SCC25-GF-CM significantly enhanced SCC25 proliferation compared to CM from control GFs (Fig. 1g). Moreover, in a scratch wound healing assay, SCC25-GF-CM promoted more efficient wound closure than control GF-CM, indicating enhanced cell motility (Fig. 1h). Likewise, transwell invasion assays showed a significant increase in the number of invasive SCC25 cells in the SCC25-GF-CM group (Fig. 1i). Collectively, these results demonstrate that OSCC-derived factors can induce CAF differentiation in GFs via paracrine signaling and that these differentiated CAFs, in turn, enhance OSCC cell proliferation, migration, and invasion. The findings further suggest that factors other than TGF-β1 in SCC25 CM are critical for driving CAF-mediated tumor-promoting phenotypes.

### CXCL1 is a crucial factor in OSCC CM-induced CAF differentiation and its prognostic implications in patients with OSCC

**Fig. 2:**
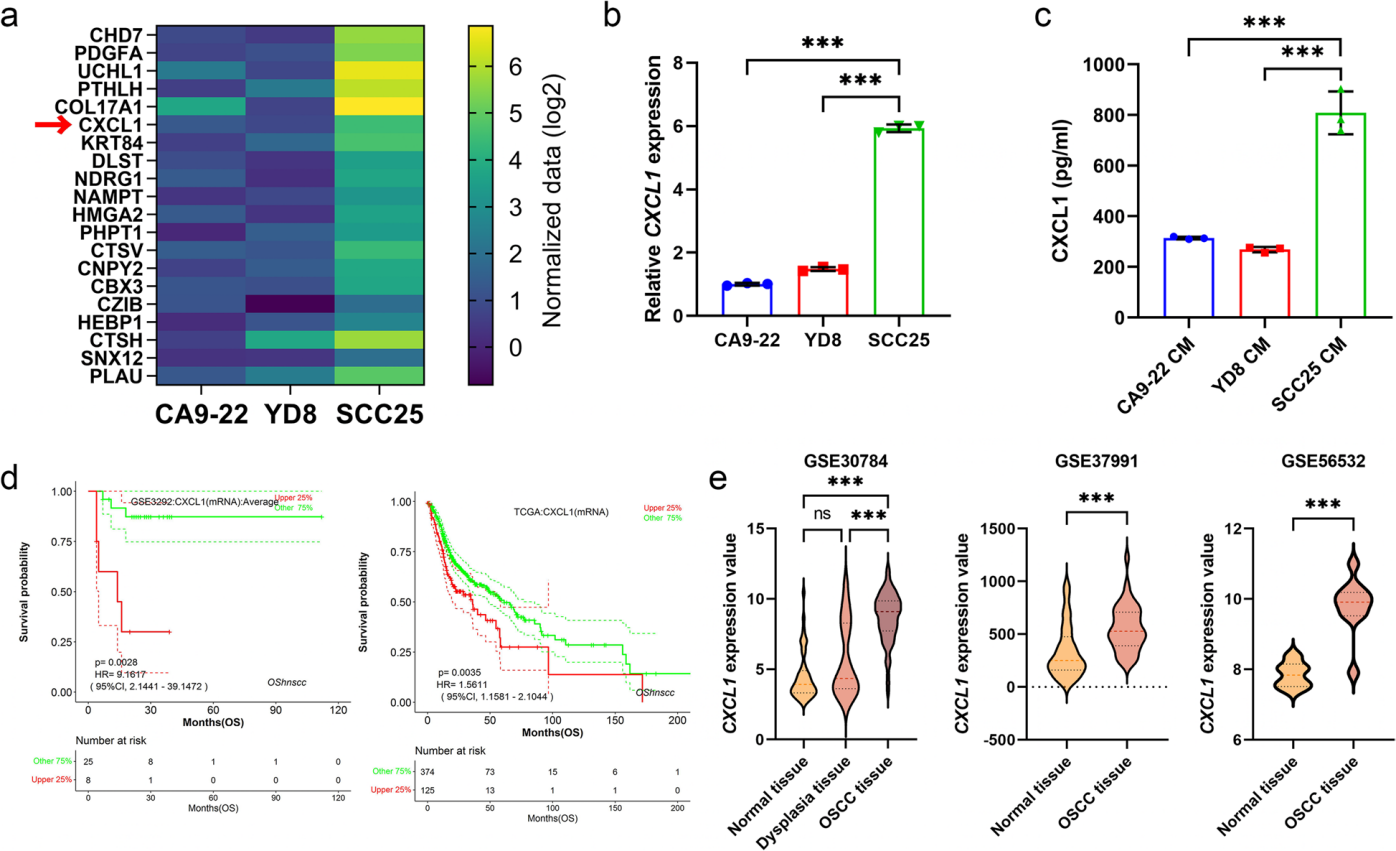
CXCL1 expression in OSCC CM and its prognostic significance in patients with OSCC. **a** Secretomic analysis of OSCC CM: Heatmap showing the expression of 20 significant proteins in CM derived from three oral cancer cell lines CA9-22, YD8, and SCC25. Data represent a single secretome analysis. **b** mRNA expression of *CXCL1* was analyzed using qRT-PCR in CA9-22, YD8, and SCC25 cells. **c** Secreted CXCL1 proteins in OSCC CM were measured using ELISA. **d** Kaplan–Meier curve showing overall survival based on *CXCL1* expression in head and neck squamous cell carcinoma using data from GSE3292 and TCGA. **e** Comparison of *CXCL1* expression between normal individuals and patients with OSCC using data from the GEO database. ^***^p < 0.001; OS, overall survival; TCGA, The Cancer Genome Atlas; ns, not significant

To further investigate the factors contributing to CAF differentiation, we hypothesized that SCC25 CM contains elements beyond TGF-β1 that enhance CAF differentiation. To explore this, we performed liquid chromatography-mass spectrometry (LC–MS/MS) analysis on OSCC CM to identify potential CAF-inducing proteins. We identified 1,129 proteins in the OSCC CM and primarily screened those that were more highly expressed in SCC25 cells, as SCC25 CM induced CAF differentiation at a higher level than CA9-22 and YD8 cells in a previous experiment (Table S1). Among the top 20 proteins showing relatively high expression in SCC25 cells (Fig. 2a), we focused on CXCL1, which was recently reported to be significant in oral cancer [21]. Although PDGFA, which has also been positively correlated with OSCC progression [22], was identified as a highly expressed protein, our analysis of GEO datasets revealed inconsistent trends in PDGFA expression across the datasets (Fig. S1). In contrast, CXCL1 showed a more robust and consistent pattern of upregulation, further supporting its selection as the key focus of this study. To validate the secretomic data, the mRNA expression levels of *CXCL1* in OSCC cell lines were verified using qRT-PCR (Fig. 2b). To further validate the protein expression levels of CXCL1 in OSCC CM, we conducted an ELISA, which revealed that CXCL1 levels were significantly higher in SCC25 CM compared to CA9-22 and YD8 CM (Fig. 2c). The Kaplan–Meier survival curve showed that patients with OSCC with high CXCL1 expression had a significantly lower overall survival probability than did those with lower expression (GSE3292- HR 9.1617; 95% CI: 2.1441–39.1472; p = 0.0028, TCGA-HR 1.5611; 95% CI: 1.1581–2.1044; p = 0.0035), suggesting that elevated CXCL1 expression was associated with a poorer prognosis in patients with OSCC (Fig. 2d). Additionally, public GEO data showed that CXCL1 expression was markedly increased in OSCC tissues compared to in normal tissues (Fig. 2e). These findings suggest that CXCL1 may serve not only as a prognostic biomarker but also as a potential therapeutic target in OSCC.

### CXCL1 potentiates TGF-β1-induced CAF differentiation in GFs by activating Smad2/3 and AKT signaling

**Fig. 3:**
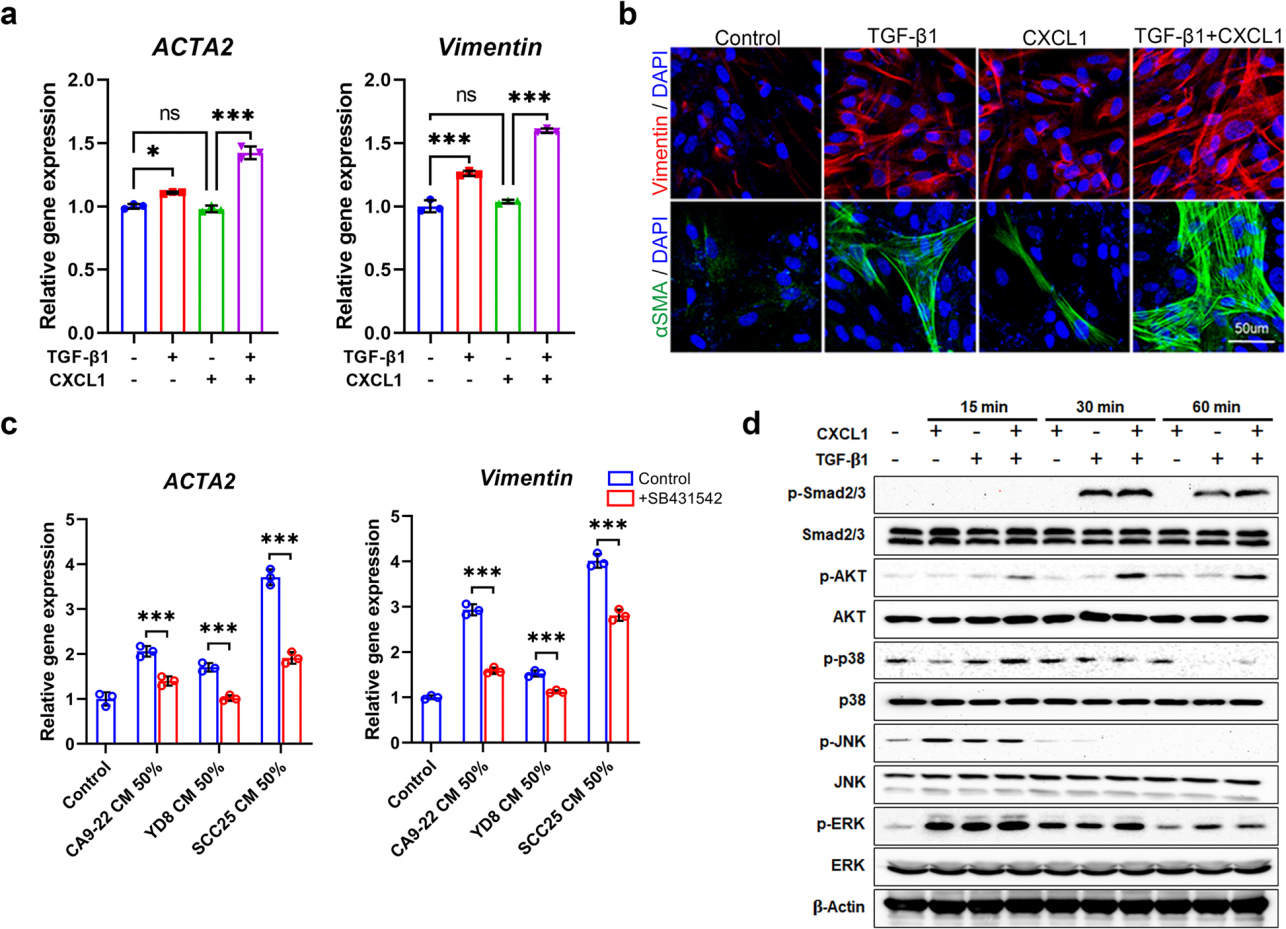
CXCL1 upregulates TGF-β1-mediated expression of ACTA2 and vimentin in GFs by activating the Smad2/3 and AKT pathways. GFs were treated with TGF-β1 (10 ng/mL), either alone or in combination with CXCL1 (10 ng/mL) for 96 h. **a** mRNA expression of *ACTA2* and *vimentin* was analyzed using qRT-PCR. **b** Immunocytochemistry was performed to determine the protein expression of vimentin (red) and αSMA (green). Nuclei were counterstained with DAPI (blue). Scale bar = 50 μm. **c** GFs were pretreated with the TGF-β1 inhibitor SB431542 (1 µM) for 30 min, followed by treatment with OSCC CM in the presence of the inhibitor for 96 h. mRNA expression levels of *ACTA2* and *vimentin* were analyzed using qRT-PCR. **d** GFs were treated with TGF-β1, either alone or in combination with CXCL1, for the indicated time. Phosphorylation of Smad2/3, AKT, p38, JNK, and ERK was analyzed using western blotting. ^*^p < 0.05, ^***^p < 0.001. ns, not significant; DAPI, 4′,6-diamidino-2-phenylindole

Our proteomic and qRT-PCR analyses identified CXCL1 as a highly expressed factor in SCC25-conditioned medium, strongly associated with poor prognosis in OSCC patients and suggesting its role as both a prognostic marker and a mediator of CAF differentiation. Based on these results, we hypothesized that CXCL1 plays a functional role in SCC25-induced GF-to-CAF differentiation, which was investigated further in subsequent experiments. To examine the role of CXCL1 in CAF differentiation, we added recombinant CXCL1 protein during the TGF-β1-mediated CAF differentiation of GFs. Administration of CXCL1 alone did not stimulate *ACTA2* and *vimentin* mRNA expression in GFs but significantly induced these CAF markers in the presence of TGF-β1 (Fig. 3a). These results were validated at the protein level using immunofluorescence staining (Fig. 3b). Furthermore, pre-treatment of GFs with the TGF-β receptor inhibitor SB431542 significantly reduced the CAF differentiation-inducing effects of OSCC-conditioned medium, as evidenced by decreased *ACTA2* and *vimentin* mRNA expression (Fig. 3c). This result further confirms that TGF-β1 signaling is essential for CAF differentiation and highlights the synergistic role of CXCL1 in enhancing TGF-β1-mediated effects. Accumulating evidence indicates that the TGF-β, AKT, and MAPK pathways are positively correlated with the differentiation of various cells into CAFs [23]. We performed western blotting to identify the signaling pathways involved in TGF-β1 and CXCL1-mediated CAF differentiation. CXCL1 or TGF-β1 treatment induced phosphorylation of Smad2/3 and AKT. This effect was markedly enhanced when both were administered together, peaking 60 min after treatment. Although phosphorylation of JNK and ERK was induced by CXCL1 or TGF-β1 after 15 min, co-treatment did not result in a synergistic increase. This result indicates that the Smad2/3 and AKT signaling pathways are involved in CXCL1 and TGF-β1-induced CAF differentiation of GFs (Fig. 3d).

### CXCR2 mediates CXCL1-induced CAF differentiation and pro-tumorigenic activity via AKT signaling in GFs

**Fig. 4:**
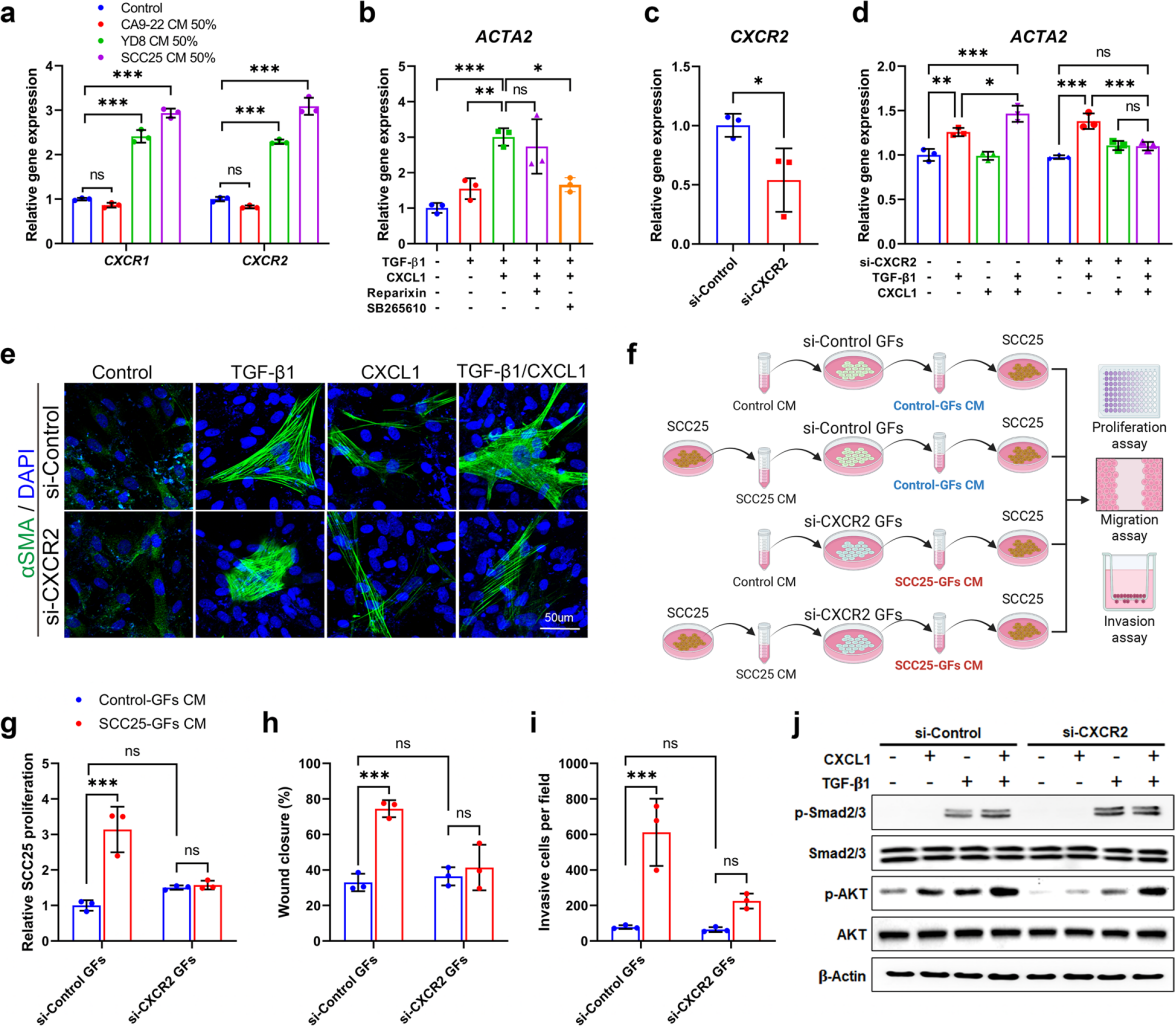
CXCR2 is responsible for CXCL1-induced CAF differentiation in GFs via an AKT-dependent pathway. **a** mRNA expression of *CXCR1* and *CXCR2* in GFs treated with OSCC CM for 48 h was analyzed using qRT-PCR. **b** GFs were pretreated with reparixin (1 µM) or SB265610 (10 µM) for 30 min, followed by treatment with TGF-β1 (10 ng/mL) and CXCL1 (10 ng/mL) for 96 h. mRNA expression of *ACTA2* was analyzed using qRT-PCR. **c** Endogenous expression of CXCR2 in GFs was reduced by transfection with CXCR2-specific siRNA. mRNA expression of *CXCR2* in GFs was analyzed using qRT-PCR. **d** mRNA expression of *ACTA2* was analyzed using qRT-PCR in GFs treated with TGF-β1, either alone or in combination with CXCL1. **e** Immunocytochemistry was performed to determine the protein expression of αSMA (green). Nuclei were counterstained with DAPI (blue). Scale bar = 50 μm. **f** Schematic illustration of the experimental procedure for CM-based assays, including proliferation, migration, and invasion experiments in SCC25 cells using CM derived from si-Control or si-CXCR2 GFs treated with either control CM or SCC25 CM. **g** SCC25 cell proliferation was analyzed using the cell counting kit-8 assay. Illustration was created with BioRender.com. **h** Migration of SCC25 cells was analyzed by scratch wound healing assay. **i** SCC25 cell invasion was evaluated using transwell inserts. **j** Phosphorylation of Smad2/3 and AKT was analyzed using western blotting in GFs transfected with either si-Control- or si-CXCR2-specific RNA. ^*^p < 0.05, ^**^p < 0.01, ^***^p < 0.001. ns, not significant; DAPI, 4′,6-diamidino-2-phenylindole

To investigate which receptor mediates CXCL1-induced CAF differentiation, we analyzed the expression levels of CXCR1 and CXCR2, both key receptors for CXCL1 [24], using qRT-PCR. The results showed that treatment with YD8 and SCC25 CM significantly increased the mRNA expression of both *CXCR1* and *CXCR2* compared to the untreated control (Fig. 4a). To further clarify which receptor plays a dominant role in CXCL1-mediated CAF differentiation, inhibitor experiments were performed. Reparixin, which targets both CXCR1 and CXCR2 but is slightly more specific to CXCR1 due to its lower IC50, and SB265610, a CXCR2-specific inhibitor, were used. The results revealed that the CXCL1- and TGF-β1-induced increase in *ACTA2* expression was significantly reduced by pretreatment with SB265610, bringing *ACTA2* levels down to those observed with TGF-β1 treatment alone. In contrast, reparixin had a less pronounced effect. These findings indicate that most of the CXCL1-driven effects on *ACTA2* expression are abolished by SB265610, suggesting that CXCL1 promotes CAF differentiation predominantly through a CXCR2-dependent mechanism (Fig. 4b). To further confirm the role of CXCR2 in CXCL1-induced CAF differentiation, we performed siRNA transfection to specifically silence CXCR2 expression in GFs. Transfection with CXCR2-specific siRNA effectively downregulated *CXCR2* mRNA expression by approximately 47% in GFs, as validated by qRT-PCR (Fig. 4c). The suppression of CXCR2 expression eliminated the CXCL1-mediated upregulation of *ACTA2* mRNA, resulting in levels comparable to the untreated condition (Fig. 4d). To support these findings at the protein level, immunofluorescence staining was conducted to assess the expression of αSMA. The results demonstrated that silencing CXCR2 markedly diminished the CXCL1-enhanced αSMA protein expression in GFs (Fig. 4e). To evaluate whether CXCR2 knockdown attenuates the pro-tumorigenic activity of CAFs, SCC25 cells were treated with CM derived from si-Control or si-CXCR2 GFs preconditioned with either control or SCC25 CM (Fig. 4f). CM from SCC25 CM-stimulated GFs markedly enhanced SCC25 cell proliferation, whereas this pro-proliferative effect was abrogated when CXCR2 was silenced in GFs (Fig. 4g). Similarly, the enhanced migratory capacity of SCC25 cells observed with CM from SCC25-activated GFs was diminished upon CXCR2 knockdown, as shown by reduced wound closure in scratch assays (Fig. 4h). Consistently, the increase in SCC25 cell invasion induced by CAF-derived CM was also attenuated when CXCR2 expression in GFs was suppressed (Fig. 4i). To explore the signaling pathways involved in the CXCL1-CXCR2 axis during CAF differentiation, western blot analysis was performed to examine the phosphorylation status of Smad2/3 and AKT in GFs transfected with CXCR2-specific siRNA. While silencing CXCR2 did not affect Smad2/3 phosphorylation, it resulted in a significant decrease in AKT phosphorylation, suggesting that the AKT signaling pathway plays a critical role in CXCL1-CXCR2-mediated CAF differentiation (Fig. 4j). These results indicate that the differentiation and functional activity of CAFs by CXCL1 are mediated through a CXCR2-dependent pathway involving AKT signaling.

### Stromal CXCR2 drives OSCC tumor expansion in a xenograft model

**Fig. 5:**
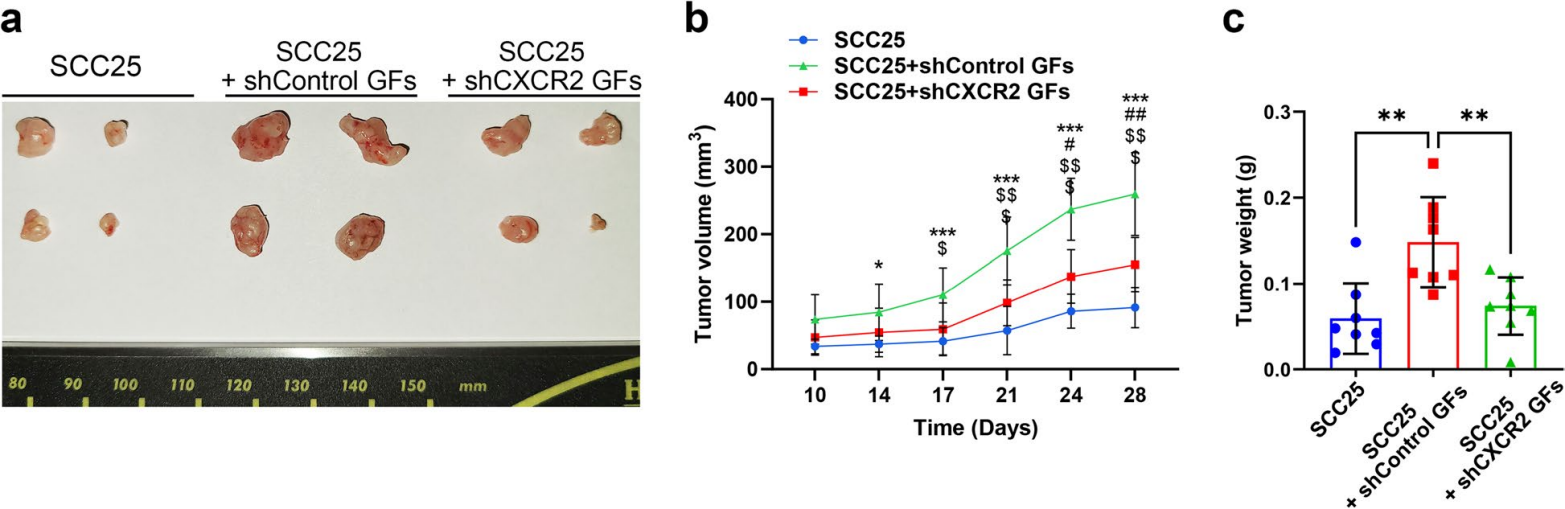
Knockdown of CXCR2 resulted in reduced SCC25 tumor growth mediated by GFs in vivo. SCC25 cells, either alone or mixed with shControl GFs or shCXCR2 GFs, were implanted into nude mice (n = 8; two independent experiments with 4 mice per group). **a** Representative images of SCC25 tumors obtained from nude mice (shown from one of two independent experimental replicates). **b** Tumor volume was monitored on the indicated days. **c** Tumor weight was measured on day 28. Data are presented as mean ± SD. Statistical analysis was performed using one-way ANOVA followed by Bonferroni post-hoc test. ^*^p < 0.05, ^**^p < 0.01, ^***^p < 0.001; *SCC25 vs SCC25 + shControl GFs; ^#^SCC25 vs SCC25 + shCXCR2 GFs; ^$^SCC25 + shControl GFs vs SCC25 + shCXCR2 GFs

To investigate whether CXCR2 downregulation contributes to tumor progression by affecting CAF differentiation in vivo, we examined the role of CXCR2 in stromal cells using a murine tumor xenograft model. SCC25 cells were transplanted subcutaneously either alone or with GFs transfected with shControl (shControl GFs) or shCXCR2 (shCXCR2 GFs) lentiviruses. Transplantation of SCC25 cells results in tumor formation. Co-transplantation with shControl GFs led to a significant increase in tumor size over time compared with the effects of SCC25 cells alone. In contrast, co-transplantation with shCXCR2 GFs caused a notable reduction in the tumor volume compared with the effects of co-transplantation with shControl GFs (Fig. 5a and b). Consistent with this result, co-transplantation with shControl GFs increased SCC25 xenograft tumor weight, whereas co-transplantation with shCXCR2 GFs did not (Fig. 5c). This result indicates that CXCR2 has a crucial role in GF-stimulated tumor growth of SCC25 cells in vivo.

### CXCR2 promotes CAF differentiation in the TME

**Fig. 6:**
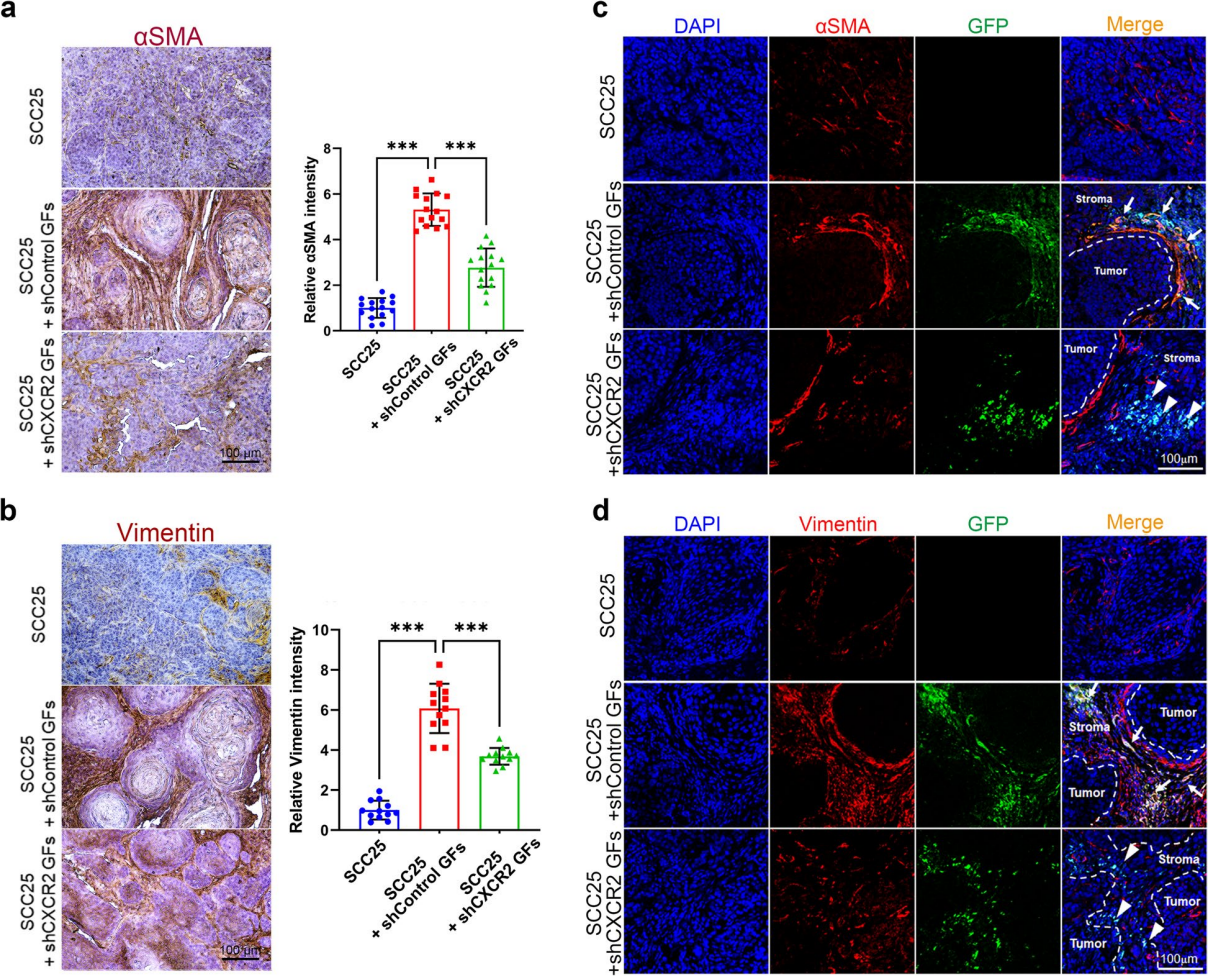
Implication of CXCR2 on the differentiation of CAFs in the tumor microenvironment. **a** Immunohistochemical images (left) and quantitative analysis (right) of αSMA expression in SCC25 tumors. **b** Immunohistochemical images (left) and quantitative analysis (right) of vimentin expression in SCC25 tumors. **c**, **d** Expression of αSMA and vimentin in xenograft tissues was determined using immunofluorescence staining. Transplanted GFs were tracked using GFP delivered by lentivirus. Images of αSMA or vimentin expression (red) were overlaid with images of GFs (GFP-positive cells, green) and nuclei (DAPI, blue). Arrows indicate co-expression of αSMA and vimentin in GFP-positive cells within tumor tissues transplanted with SCC25 plus shControl GFs. Arrowheads indicate the absence of αSMA and vimentin expression in GFP-positive cells within tumor tissues transplanted with SCC25 plus shCXCR2 GFs. Scale bar = 100 μm. Data are presented as mean ± SD. Statistical analysis was performed using one-way ANOVA followed by Bonferroni post-hoc test. ^***^p < 0.001. GFP, green fluorescent protein; DAPI, 4′,6-diamidino-2-phenylindole

To determine whether CXCR2 is involved in the differentiation of GFs to CAFs in vivo, we examined αSMA and vimentin expression in xenograft tumors using immunohistochemical analysis. The intensity of αSMA and vimentin staining was increased in xenograft tumors with co-transplantation of SCC25 cells and shControl GFs compared with that in SCC25 cells alone. However, co-transplantation with shCXCR2 GFs resulted in a reduced staining intensity compared with that in shControl GFs (Fig. 6a and b). To assess whether CXCR2 is responsible for the differentiation of GFs into αSMA- or vimentin-expressing CAFs within tumors, we conducted immunofluorescence double staining of tumor tissues. The transplanted GFs were tracked using GFP delivered by a lentivirus. In tumors co-transplanted with SCC25 cells and shControl GFs, αSMA- and vimentin-positive immunoreactivity was primarily observed in GFP-expressing GFs. However, this immunoreactivity was rarely observed in GFP-expressing GFs when SCC25 cells were co-transplanted with shCXCR2 GFs (Fig. 6c and d). These results suggest that CXCR2 is responsible for CAF differentiation in TME.

## Discussion

CXCL1, a chemokine known for its role in inflammation and wound healing, has been implicated in tumor progression and the modulation of the TME. In OSCC, CXCL1 secretion was shown to induce the transformation of normal fibroblasts into senescent CAFs via an autocrine loop [25]. However, our findings reveal that CXCL1 alone is insufficient to directly induce CAF differentiation in gingival fibroblasts. Instead, CXCL1 acts as a co-stimulatory factor, significantly enhancing the differentiation process in the presence of TGF-β1. Our western blot analysis showed that co-treatment with CXCL1 and TGF-β1 led to enhanced phosphorylation of both Smad2/3 and AKT compared to each factor alone, suggesting that these pathways cooperate during CAF differentiation. To further delineate their roles, CXCR2 knockdown experiments demonstrated that inhibition of CXCL1 signaling selectively reduced AKT phosphorylation without affecting Smad2/3, highlighting AKT as the dominant CXCL1-responsive pathway. These results suggest a model in which TGF-β1 initiates canonical Smad2/3 signaling, while CXCL1 amplifies the differentiation response through AKT activation, leading to a more robust induction of CAF marker expression. This interplay between Smad2/3 and AKT represents a potential mechanism by which TGF-β1 and CXCL1 synergize to promote myofibroblastic transformation. The AKT signaling pathway is a critical mediator of cellular processes such as growth, survival, and migration, and it has been extensively studied in the context of cancer [26]. The aberrant activation of the phosphoinositide 3‐kinase (PI3K)/AKT signaling cascade in OSCC is associated with the suppression of apoptosis, increased drug resistance, enhanced angiogenesis, metastasis promotion, and the induction of EMT [27]. In CAFs, AKT signaling has been shown to be crucial for their infiltration and function within tumors. Specifically, Integrin β2 is overexpressed in CAFs within OSCC, where it enhances glycolytic activity by mechanically influencing the PI3K/AKT/mTOR pathway [28]. These findings align with the hypothesis that CAFs play an active role in shaping the metabolic and signaling landscapes of OSCC tumors. Furthermore, TGF-β1, a well-known regulator of CAF differentiation, has been shown to interact with CXCL1 expression. In mammary carcinoma-associated fibroblasts, TGF-β negatively regulates CXCL1 expression through Smad2/3 and HGF/c-Met signaling mechanisms [29]. This regulatory relationship demonstrates the dual role of TGF-β1 in CAF biology, acting both as a driver of differentiation and as a modulator of chemokine networks, including CXCL1. Notably, in esophageal squamous cell carcinoma (ESCC), TGF-β1-induced LAMC1 expression promotes CXCL1 secretion, which in turn activates the AKT-NFκB signaling axis, leading to the formation of inflammatory CAFs (iCAFs) [30]. The existing literature provides robust evidence that CXCL1 can regulate CAF differentiation through the AKT pathway, driven upstream signals such as TGF-β1. This is consistent with our findings in this study, where we demonstrated that OSCC-derived CXCL1 enhances the differentiation of GFs into CAFs via an AKT-dependent signaling pathway, but only in the presence of TGF-β1. These results highlight the cooperative dynamics of CXCL1 and TGF-β1 in orchestrating CAF differentiation and underscore the multifaceted interactions between key signaling pathways within the tumor microenvironment.

CAFs promote cancer cell proliferation across various tumor types [31, 32]. CM derived from CAFs stimulates the proliferation of head and neck squamous cell carcinoma cells in vitro, which is consistent with findings showing that various secreted cytokines and growth factors can drive tumor growth [33]. For example, interleukin (IL)−6 upregulates osteopontin (SPP1) expression in head and neck squamous cell carcinoma cells, leading to increased proliferation via integrin/NF-κB signaling [34]. Bae et al. reported a case of reciprocal paracrine communication between tumor cells and CAFs, where the volume of orthotopic tumors was associated with the number of CAFs co-injected with tumor cells [35]. Their study showed that IL-1α secreted by OSCC cells drove CAF proliferation and increased cytokine production, including that of CCL7, CXCL1, and IL-8. Similarly, we found that αSMA- or vimentin-expressing CAFs facilitated OSCC cell proliferation both in vitro and in vivo, suggesting that these CAF subtypes promote OSCC progression. While αSMA and vimentin expression levels suggest an increased CAF differentiation in SCC25 cells co-transplanted with shControl GFs, the proliferation observed in Fig. 5a reflects additional tumor-promoting mechanisms beyond differentiation status alone. Specifically, well-differentiated tumors may exhibit enhanced stromal interactions that facilitate growth, as evidenced by the robust tumor expansion in the SCC25 group co-transplanted with shControl GFs.

Although numerous studies have emphasized the tumor-promoting actions of CAFs, evidence suggests that CAFs play a role in restraining cancer growth, potentially serving as a defense mechanism against tumor formation [36]. This apparent contradiction may be attributed to the remarkable heterogeneity and plasticity of CAFs. In fact, several distinct CAF subtypes have been identified, each with unique molecular markers and functions, such as myofibroblastic CAFs, inflammatory CAFs, and antigen-presenting CAFs [37, 38]. In the current study, the identified CAFs exhibited high expression of αSMA, which is characteristic of myofibroblastic CAFs, while also showing significantly increased expression of CXCL1, suggesting features of inflammatory CAFs. The functional heterogeneity of CAFs has important therapeutic implications. Certain CAF subtypes may promote tumor progression through immune suppression or matrix remodeling, while others may restrain growth. Therefore, targeting stromal factors like CXCL1 or its receptor CXCR2 could offer a means of modulating specific CAF populations rather than globally depleting CAFs, which has been shown to yield adverse outcomes in some contexts.

Head and neck squamous cell carcinomas, including OSCC, are characterized by elevated CXCL1 expression compared to normal tissues [14, 39]. Bioinformatics analyses identified CXCL1 as a hub gene with significant protein–protein interactions in oral cancer, particularly in OSCC and oral tongue squamous cell carcinoma [40–42]. Elevated CXCL1 levels in the blood of patients with head and neck cancer have been linked to radiation therapy failure [43], and higher CXCL1 expression in tumors is associated with a worse prognosis in head and neck squamous cell carcinoma [44]. CXCL1 may be involved in the development of head and neck cancer, as its expression is elevated in fibroblasts during oral submucosal fibrosis, a precancerous condition that can lead to head and neck cancer. In oral submucosal fibrosis, CXCL1 drives tumorigenic processes, including keratinocyte proliferation and migration, which contribute to the transition from oral submucosal fibrosis to head and neck cancer [45]. Our findings support those of previous studies, showing that CXCL1 promotes the differentiation of GFs into CAFs, which, in turn, may contribute to OSCC progression. Furthermore, the GEO database revealed that CXCL1 expression increased with OSCC progression (Fig. 2d; GSE30784).

Targeting CXCL1 for anticancer therapy is theoretically possible; however, CXCR2, the primary receptor of CXCL1, is a more comprehensive and practical therapeutic target. CXCR2 is not only activated by CXCL1 but also by several other CXC chemokines, including CXCL2, CXCL3, CXCL5, CXCL6, and CXCL8, all of which are closely associated with cancer progression and the tumor microenvironment [46]. By targeting CXCR2, it is possible to disrupt multiple pro-tumorigenic signaling pathways simultaneously, providing a broader therapeutic impact compared to solely targeting CXCL1. Moreover, our experimental findings further support this rationale. We demonstrated that CXCR2 is essential for mediating the effects of CXCL1 in GF-to-CAF differentiation, a critical process in the progression of OSCC. Specifically, knocking down CXCR2 in GFs effectively blocked CAF differentiation and reduced tumor-promoting activity, as shown in our in vitro and in vivo models. This indicates that CXCR2 serves as a functional bottleneck for the CXCL1-driven paracrine signaling cascade, making it a strategically advantageous therapeutic target. Importantly, our in vivo findings corroborate the in vitro results, demonstrating that the CXCL1–CXCR2 axis not only induces CAF differentiation under controlled culture conditions, but also plays a central role in modulating tumor growth and stromal composition in vivo. The consistent suppression of CAF markers and tumor volume upon CXCR2 knockdown in both settings highlights a mechanistically conserved role for this signaling pathway across cellular and organismal levels. This integration strengthens the translational relevance of targeting CXCR2 in OSCC. Additionally, CXCR2 antagonists such as SB225002 and SCH-527123 have shown promising preclinical anticancer activity, inhibiting tumor cell proliferation, migration, and therapy resistance across multiple cancer types [47–50]. These compounds highlight the feasibility of CXCR2 as a druggable target. Current clinical trials exploring CXCR2 inhibitors further validate its potential as an effective therapeutic strategy [51]. Nevertheless, given the presence of multiple CXCR2 ligands, the possibility of compensatory mechanisms and redundant signaling should be considered. Tumors may adapt by upregulating alternative CXCR2 ligands such as CXCL2 or CXCL8 in response to CXCL1 inhibition, potentially undermining therapeutic efficacy. This underscores the value of targeting the receptor itself rather than individual ligands. Beyond OSCC, CXCL1 expression has also been associated with stromal activation in oral potentially malignant disorders such as oral submucosal fibrosis and oral lichen planus (OLP). Recent studies have highlighted molecular overlaps between OLP and early-stage OSCC, particularly in inflammation-driven pathways that may promote malignant transformation [52, 53]. Although our study did not directly examine OLP-derived tissues, the CXCL1–CXCR2 axis identified here may represent a common stromal mechanism linking chronic inflammation to tumor progression. Further studies are warranted to explore the relevance of this signaling pathway in preneoplastic conditions.

In summary, targeting the CXCL1–CXCR2 axis holds significant promise for future cancer therapies, and focusing on CXCR2 allows for a more comprehensive disruption of tumor-promoting signaling pathways, as well as direct translational applicability. Nonetheless, several limitations of this study should be acknowledged to guide the interpretation of our findings and highlight directions for future research. First, our analysis is based on bulk-level gene expression and immunohistochemical characterization, which limits the ability to resolve CXCL1 expression at the level of specific stromal subpopulations, such as CAFs. Although recent OSCC single-cell RNA sequencing datasets have emerged, such analyses were beyond the scope of the current study. Second, we primarily employed αSMA and vimentin as markers for CAFs, but these do not fully capture the phenotypic heterogeneity of CAF subsets. Further classification using additional markers such as IL-6, CXCL12, PDPN, or MHC class II would allow for more precise delineation of CAF subtypes. Third, although we identified CXCR2 as a promising therapeutic target, the presence of multiple CXCR2 ligands raises the possibility of signaling redundancy. Further investigation into these contexts will be essential to clarify the precise role and therapeutic relevance of the CXCL1–CXCR2 axis in OSCC and related pathological conditions.

## Materials and methods

### Reagents and cell culture

Various cell culture reagents including α-MEM, DMEM, RPMI 1640, and DPBS were obtained from Welgene (Daegu, Republic of Korea), and fetal bovine serum was purchased from Gibco (Grand Island, NY, USA). Recombinant human CXCL1 and TGF-β1 were purchased from PeproTech (Rocky Hill, NJ, USA). Reparixin and SB265610 were obtained from MedChemExpress (Monmouth Junction, NJ, USA), and SB431542 was purchased from Selleckchem (Houston, TX, USA). Human GFs, YD8, CA9-22, and SCC25 OSCC cell lines were cultured in appropriate media supplemented with 10% FBS at 37 °C in a humidified incubator with 5% CO₂. All cell lines were confirmed to be mycoplasma-free prior to experimentation (Fig. S2). Detailed information on antibodies is provided in Table S2.

### Transwell assay

Transwell co-culture experiments were conducted using 24-well plates equipped with insert membranes featuring 0.4 µm pores (SPL, Pocheon, Republic of Korea). GFs and OSCC cells were plated in the lower and upper chambers, respectively, each at a density of 2.5 × 10^4^ cells per compartment. The co-culture was maintained for 96 h, and the medium was refreshed every 48 h.

### Preparation of conditioned medium

To generate OSCC-derived CM, cells were seeded in 150 mm culture dishes and allowed to reach subconfluence. After rinsing three times with DPBS to eliminate serum traces, the cells were incubated with 20 mL serum-free α-MEM for 48 h. For GF-derived CM, cells were cultured with serum-free α-MEM containing either vehicle or 50% OSCC CM for 48 h. The collected media were centrifuged at 3000 × g for 10 min to remove debris, passed through a 0.2 µm syringe filter (Sartorius, Göttingen, Germany), and stored at − 70 °C until use.

### ELISA

The concentrations of secreted CXCL1 and TGF-β1 in CM samples were quantified using ELISA kits (Invitrogen, Carlsbad, CA, USA) according to the manufacturer’s instructions.

### Immunocytochemistry

Cells were fixed using 4% paraformaldehyde and subsequently permeabilized in 0.2% Triton X-100. After blocking nonspecific binding sites with 5% bovine serum albumin, the samples were incubated with primary antibodies, followed by species-appropriate Alexa Fluor-conjugated secondary antibodies. To visualize the nuclei, cells were stained with DAPI (4′,6-diamidino-2-phenylindole), and images were captured using a Carl Zeiss LSM 700 confocal microscope (Jena, Germany).

### Cell counting kit 8 assay

SCC25 cells were plated in 96-well microplates at a density of 5 × 10^3^ cells per well. At the designated time points, 20 µL of CCK-8 reagent was added to each well, and the plates were incubated under standard culture conditions. Optical density was recorded at 450 nm using a microplate reader (Opsys MR, DYNEX Technologies, Denkendorf, Germany) to determine relative cell viability.

### Cell migration and invasion assays

SCC25 cell motility was evaluated using scratch wound healing and transwell invasion assays. For the scratch assay, confluent monolayers in 24-well plates were scratched with a pipette tip and incubated for 24 h in medium or conditioned medium (CM) containing 1% FBS. Wound closure was quantified using JuLI-STAT software (NanoEnTek, Republic of Korea). For the transwell invasion assay, 5 × 10^4^ SCC25 cells in serum-free medium were seeded into Matrigel-coated transwell inserts (8 μm pore size), with CM or control medium placed in the lower chamber. After 16 h, invaded cells on the lower membrane surface were fixed, stained with 0.1% crystal violet, and counted under a microscope.

### Gene expression and survival analysis from public datasets

Gene expression microarray datasets (GSE30784, GSE37991, GSE56532) were obtained from the Gene Expression Omnibus (GEO) database, and CXCL1 expression levels in human tissue samples were analyzed using the GEO2R tool. Kaplan–Meier survival analyses were performed using the Online Consensus Survival Tool for Head and Neck Squamous Cell Carcinoma based on CXCL1 expression in cohorts including GSE3292 and TCGA (https://bioinfo.henu.edu.cn/DatabaseList.jsp) [54]. Analyses were conducted using the following parameters: overall survival (OS) as the endpoint, patient stratification by the upper 25% expression cutoff, and inclusion of all TNM stages. The TCGA and GEO survival data used in this tool were based on the database version available as of April 2018.

### LC–MS/MS–based proteomic analysis

Protein concentration was measured using the BCA assay, and 100 µg of protein per sample was digested using a filter-aided sample preparation protocol with TCEP reduction and iodoacetic acid alkylation. Tryptic digestion was performed at 37 °C for 18 h, and the resulting peptides were acidified, desalted using C18 spin columns, and eluted in 80% acetonitrile with 0.1% formic acid. Peptide separation and analysis were carried out on a SCIEX TripleTOF 5600 + mass spectrometer coupled to an ekspert™ nanoLC 425 system. SWATH-MS acquisition was performed with 100 variable m/z windows across the 400–1250 m/z range, and MS2 spectra were collected in high-sensitivity mode. DIA data were analyzed against a human UniProt spectral library using a 1% FDR cutoff, and quantification results were log₂-transformed and statistically compared using paired t-tests with Benjamini–Hochberg correction (q < 0.05).

### Quantitative real-time PCR (qRT-PCR)

Using the RNeasy Mini Kit (Qiagen, Hilden, Germany), total RNA was extracted as per the manufacturer's guidelines, and 2 μg of the extracted RNA was used for cDNA synthesis with Superscript II reverse transcriptase (Invitrogen, Carlsbad, CA, USA). For qPCR, 50 ng of cDNA was combined with SYBR Green PCR Master Mix (Applied Biosystems, Foster City, CA, USA) and amplified for 40 cycles using the AB7500 real-time PCR system (Applied Biosystems). All reactions were performed in triplicate, and gene expression was normalized to β-actin. Quantitative analysis of gene expression was performed using the 2^–ΔΔCt^ approach, with the specific primer sequences detailed in the Table S3.

### Transfection with small interfering RNA (siRNA)

CXCR2-targeting and control siRNAs were obtained from Thermo Fisher Scientific. Transfections were carried out using jetPRIME reagent (Polyplus, Berkeley, CA, USA) according to the manufacturer's instructions. Briefly, siRNAs were mixed with jetPRIME buffer and reagent, incubated for 10 min, and then added to GFs. Following a 6-h incubation, the medium was replaced.

### Western blot analysis

Cells were lysed in RIPA buffer containing standard protease and phosphatase inhibitors. Equal amounts of protein (20 μg) were resolved on SDS–polyacrylamide gels and transferred to nitrocellulose membranes. Membranes were blocked with 5% skim milk for 1 h, then sequentially incubated with primary and HRP-conjugated secondary antibodies. Signal detection was performed using enhanced chemiluminescence reagents (Amersham, UK).

### Short hairpin RNA (shRNA) lentivirus-mediated depletion of CXCR2

Lentiviral constructs targeting CXCR2 (shCXCR2) and a non-targeting control (shControl) were generated and packaged by VectorBuilder (vector IDs: VB010000-0009 mxc and VB900047-9028rjv). Additional construct details are available at vectorbuilder.com. For transduction, GFs were incubated with shRNA-expressing lentiviruses in the presence of 5 μg/mL polybrene (Sigma-Aldrich). Stable lines were established via puromycin selection (5 μg/mL).

### In vivo xenograft tumorigenesis model

All animal procedures followed the Principles of Laboratory Animal Care and were approved by the Pusan National University Institutional Animal Use and Care Committee (PNU‐2023‐0271). Six-week-old male BALB/c-nu/nu mice (20 ± 2 g; Orient Co., Republic of Korea) were randomly assigned to three groups (n = 4 per group). To ensure reproducibility and reduce experimental bias, the xenograft study was conducted in two independent rounds using the same protocol. In each round, two injection sites per mouse were used to generate a total of eight tumors per group (n = 8 tumors per group). All tumors analyzed in this study were included (n = 8 per group) without any exclusions. Representative tumor images in Fig. 5a are from the second round only. Mice were injected subcutaneously with SCC25 cells (5 × 10⁶) alone or in combination with either shControl or shCXCR2 GFs (2.5 × 10⁶) into the dorsal flank. Two subcutaneous injections were administered per mouse to enhance reproducibility of tumor growth. Tumor dimensions were recorded twice weekly using digital calipers, and volume was estimated using the formula: (length × width^2^)/2. After 4 weeks, mice were euthanized and xenograft tumors were excised, weighed, and embedded in OCT for cryosectioning (10 µm thickness).

### Immunohistochemical and Immunofluorescence Analysis

Tissue sections were processed for immunohistochemistry (IHC) using the DAKO EnVision System (streptavidin–peroxidase method). Endogenous peroxidase activity was quenched with 0.3% hydrogen peroxide in methanol, followed by blocking with normal goat serum and incubation with primary antibodies against αSMA or vimentin. HRP-conjugated secondary reagents and diaminobenzidine were used for detection, and sections were counterstained with Mayer’s hematoxylin. For immunofluorescence staining, samples were incubated with Alexa Fluor 488-conjugated anti-GFP antibody together with anti-αSMA or anti-vimentin antibodies, followed by Alexa Fluor 555-labeled secondary antibody. Nuclei were counterstained with DAPI using VECTASHIELD mounting medium, and images were acquired using a LSM 700 confocal microscope (Carl Zeiss).

### Statistical analysis

Differences between the two groups were compared using Student’s *t*-test. For multiple comparisons, one- or two-way analysis of variance with Bonferroni post-hoc test was used. Differences were considered statistically significant at *p* < 0.05. All data are presented as the mean ± SD from at least three biologically independent experiments, each performed using separate cell preparations and independently collected conditioned media. The results shown are representative of reproducible findings across experiments with low inter-replicate variability.

## Supplementary Information

Supplementary Material 1.

## Data Availability

The data of this study are available from the corresponding author upon reasonable request.

## References

[1] Lala M, Chirovsky D, Cheng JD, Mayawala K. Clinical outcomes with therapies for previously treated recurrent/metastatic head-and-neck squamous cell carcinoma (R/M HNSCC): A systematic literature review. Oral Oncol. 2018;84:108–20. 10.1016/j.oraloncology.2018.07.005 10.1016/j.oraloncology.2018.07.005 30115469

[2] Siegel RL, Miller KD, Jemal A. Cancer statistics, 2019. CA Cancer J Clin. 2019;69(1):7–34. 10.3322/caac.21551 10.3322/caac.21551 30620402

[3] Kim H, Lee SM, Ahn KM. The epidemiological and histopathological factors for delayed local recurrence in oral squamous cell carcinoma. Maxillofac Plast Reconstr Surg. 2024;46(1):38. 10.1186/s40902-024-00443-8 10.1186/s40902-024-00443-8 PMC11557773 39531141

[4] Linsen SS, Gellrich NC, Kruskemper G. Age- and localization-dependent functional and psychosocial impairments and health related quality of life six months after OSCC therapy. Oral Oncol. 2018;81:61–8. 10.1016/j.oraloncology.2018.04.011 10.1016/j.oraloncology.2018.04.011 29884415

[5] Quail DF, Joyce JA. Microenvironmental regulation of tumor progression and metastasis. Nat Med. 2013;19(11):1423–37. 10.1038/nm.3394 10.1038/nm.3394 PMC3954707 24202395

[6] Orimo A, Gupta PB, Sgroi DC, Arenzana-Seisdedos F, Delaunay T, Naeem R et al. Stromal fibroblasts present in invasive human breast carcinomas promote tumor growth and angiogenesis through elevated SDF-1/CXCL12 secretion. Cell. 2005;121(3):335–48. 10.1016/j.cell.2005.02.034 10.1016/j.cell.2005.02.034 15882617

[7] Lv K, He T. Cancer-associated fibroblasts: heterogeneity, tumorigenicity and therapeutic targets. Mol Biomed. 2024;5(1):70. 10.1186/s43556-024-00233-8 10.1186/s43556-024-00233-8 PMC11649616 39680287

[8] Liu L, Liu L, Yao HH, Zhu ZQ, Ning ZL, Huang Q. Stromal Myofibroblasts Are Associated with Poor Prognosis in Solid Cancers: A Meta-Analysis of Published Studies. PLoS One. 2016;11(7):e0159947. 10.1371/journal.pone.0159947 10.1371/journal.pone.0159947 PMC4961396 27459365

[9] Calon A, Lonardo E, Berenguer-Llergo A, Espinet E, Hernando-Momblona X, Iglesias M et al. Stromal gene expression defines poor-prognosis subtypes in colorectal cancer. Nat Genet. 2015;47(4):320–9. 10.1038/ng.3225 10.1038/ng.3225 25706628

[10] Kalluri R. The biology and function of fibroblasts in cancer. Nat Rev Cancer. 2016;16(9):582–98. 10.1038/nrc.2016.73 10.1038/nrc.2016.73 27550820

[11] Kahounova Z, Kurfurstova D, Bouchal J, Kharaishvili G, Navratil J, Remsik J et al. The fibroblast surface markers FAP, anti-fibroblast, and FSP are expressed by cells of epithelial origin and may be altered during epithelial-to-mesenchymal transition. Cytometry A. 2018;93(9):941–51. 10.1002/cyto.a.23101 10.1002/cyto.a.23101 28383825

[12] Calvo F, Ege N, Grande-Garcia A, Hooper S, Jenkins RP, Chaudhry SI et al. Mechanotransduction and YAP-dependent matrix remodelling is required for the generation and maintenance of cancer-associated fibroblasts. Nat Cell Biol. 2013;15(6):637–46. 10.1038/ncb2756 10.1038/ncb2756 PMC3836234 23708000

[13] Acharyya S, Oskarsson T, Vanharanta S, Malladi S, Kim J, Morris PG et al. A CXCL1 paracrine network links cancer chemoresistance and metastasis. Cell. 2012;150(1):165–78. 10.1016/j.cell.2012.04.042 10.1016/j.cell.2012.04.042 PMC3528019 22770218

[14] Wei LY, Lee JJ, Yeh CY, Yang CJ, Kok SH, Ko JY et al. Reciprocal activation of cancer-associated fibroblasts and oral squamous carcinoma cells through CXCL1. Oral Oncol. 2019;88:115–23. 10.1016/j.oraloncology.2018.11.002 10.1016/j.oraloncology.2018.11.002 30616781

[15] Zhang WL, Fan HY, Chen BJ, Wang HF, Pang X, Li M et al. Cancer-associated fibroblasts-derived CXCL1 activates DEC2-mediated dormancy in oral squamous cell carcinoma. Heliyon. 2024;10(20):e39133. 10.1016/j.heliyon.2024.e39133 10.1016/j.heliyon.2024.e39133 PMC11513488 39469703

[16] Zhang S, Dong Y, Zhao S, Bi F, Xuan M, Zhu G et al. CXCL1 promoted the migration and invasion abilities of oral cancer cells and might serve as a promising marker of prognosis in tongue cancer. J Oral Pathol Med. 2023;52(7):583–92. 10.1111/jop.13418 10.1111/jop.13418 36829264

[17] Miyake M, Furuya H, Onishi S, Hokutan K, Anai S, Chan O et al. Monoclonal Antibody against CXCL1 (HL2401) as a Novel Agent in Suppressing IL6 Expression and Tumoral Growth. Theranostics. 2019;9(3):853–67. 10.7150/thno.29553 10.7150/thno.29553 PMC6376461 30809313

[18] Spaks A, Jaunalksne I, Spaka I, Chudasama D, Pirtnieks A, Krievins D. Diagnostic Value of Circulating CXC Chemokines in Non-small Cell Lung Cancer. Anticancer Res. 2015;35(12):6979–83. 26637925

[19] Keane MP, Belperio JA, Xue YY, Burdick MD, Strieter RM. Depletion of CXCR2 inhibits tumor growth and angiogenesis in a murine model of lung cancer. J Immunol. 2004;172(5):2853–60. 10.4049/jimmunol.172.5.2853 10.4049/jimmunol.172.5.2853 14978086

[20] Sharma B, Nawandar DM, Nannuru KC, Varney ML, Singh RK. Targeting CXCR2 enhances chemotherapeutic response, inhibits mammary tumor growth, angiogenesis, and lung metastasis. Mol Cancer Ther. 2013;12(5):799–808.10.1158/1535-7163.MCT-12-0529 10.1158/1535-7163.MCT-12-0529 PMC3653628 23468530

[21] Huang W, Jiang M, Lin Y, Qi Y, Li B. Crosstalk between cancer cells and macrophages promotes OSCC cell migration and invasion through a CXCL1/EGF positive feedback loop. Discov Oncol. 2024;15(1):145. 10.1007/s12672-024-00972-8 10.1007/s12672-024-00972-8 PMC11076430 38713320

[22] Lin LH, Lin JS, Yang CC, Cheng HW, Chang KW, Liu CJ. Overexpression of Platelet-Derived Growth Factor and Its Receptor Are Correlated with Oral Tumorigenesis and Poor Prognosis in Oral Squamous Cell Carcinoma. Int J Mol Sci. 2020;21(7).10.3390/ijms21072360 10.3390/ijms21072360 PMC7177415 32235327

[23] Wu F, Yang J, Liu J, Wang Y, Mu J, Zeng Q et al. Signaling pathways in cancer-associated fibroblasts and targeted therapy for cancer. Signal Transduct Target Ther. 2021;6(1):218. 10.1038/s41392-021-00641-0 10.1038/s41392-021-00641-0 PMC8190181 34108441

[24] Ahuja SK, Murphy PM. The CXC chemokines growth-regulated oncogene (GRO) alpha, GRObeta, GROgamma, neutrophil-activating peptide-2, and epithelial cell-derived neutrophil-activating peptide-78 are potent agonists for the type B, but not the type A, human interleukin-8 receptor. J Biol Chem. 1996;271(34):20545–50. 10.1074/jbc.271.34.20545 10.1074/jbc.271.34.20545 8702798

[25] Kim EK, Moon S, Kim DK, Zhang X, Kim J. CXCL1 induces senescence of cancer-associated fibroblasts via autocrine loops in oral squamous cell carcinoma. PLoS One. 2018;13(1):e0188847. 10.1371/journal.pone.0188847 10.1371/journal.pone.0188847 PMC5779641 29360827

[26] Zheng S, He S, Liang Y, Tan Y, Liu Q, Liu T et al. Understanding PI3K/Akt/mTOR signaling in squamous cell carcinoma: mutated PIK3CA as an example. Mol Biomed. 2024;5(1):13. 10.1186/s43556-024-00176-0 10.1186/s43556-024-00176-0 PMC11016524 38616230

[27] Wang J, Jiang C, Li N, Wang F, Xu Y, Shen Z et al. The circEPSTI1/mir-942–5p/LTBP2 axis regulates the progression of OSCC in the background of OSF via EMT and the PI3K/Akt/mTOR pathway. Cell Death Dis. 2020;11(8):682. 10.1038/s41419-020-02851-w 10.1038/s41419-020-02851-w PMC7443145 32826876

[28] Zhang X, Dong Y, Zhao M, Ding L, Yang X, Jing Y et al. ITGB2-mediated metabolic switch in CAFs promotes OSCC proliferation by oxidation of NADH in mitochondrial oxidative phosphorylation system. Theranostics. 2020;10(26):12044–59. 10.7150/thno.47901 10.7150/thno.47901 PMC7667693 33204328

[29] Fang WB, Mafuvadze B, Yao M, Zou A, Portsche M, Cheng N. TGF-beta Negatively Regulates CXCL1 Chemokine Expression in Mammary Fibroblasts through Enhancement of Smad2/3 and Suppression of HGF/c-Met Signaling Mechanisms. PLoS One. 2015;10(8):e0135063. 10.1371/journal.pone.0135063 10.1371/journal.pone.0135063 PMC4529193 26252654

[30] Fang L, Che Y, Zhang C, Huang J, Lei Y, Lu Z et al. LAMC1 upregulation via TGFbeta induces inflammatory cancer-associated fibroblasts in esophageal squamous cell carcinoma via NF-kappaB-CXCL1-STAT3. Mol Oncol. 2021;15(11):3125–46. 10.1002/1878-0261.13053 10.1002/1878-0261.13053 PMC8564640 34218518

[31] Curtis M, Kenny HA, Ashcroft B, Mukherjee A, Johnson A, Zhang Y et al. Fibroblasts Mobilize Tumor Cell Glycogen to Promote Proliferation and Metastasis. Cell Metab. 2019;29(1):141–55 e9. 10.1016/j.cmet.2018.08.007 10.1016/j.cmet.2018.08.007 PMC6326875 30174305

[32] Jahangiri B, Khalaj-Kondori M, Asadollahi E, Sadeghizadeh M. Cancer-associated fibroblasts enhance cell proliferation and metastasis of colorectal cancer SW480 cells by provoking long noncoding RNA UCA1. J Cell Commun Signal. 2019;13(1):53–64. 10.1007/s12079-018-0471-5 10.1007/s12079-018-0471-5 PMC6381368 29948578

[33] Wheeler SE, Shi H, Lin F, Dasari S, Bednash J, Thorne S et al. Enhancement of head and neck squamous cell carcinoma proliferation, invasion, and metastasis by tumor-associated fibroblasts in preclinical models. Head Neck. 2014;36(3):385–92. 10.1002/hed.23312 10.1002/hed.23312 PMC4111913 23728942

[34] Qin X, Yan M, Wang X, Xu Q, Wang X, Zhu X et al. Cancer-associated Fibroblast-derived IL-6 Promotes Head and Neck Cancer Progression via the Osteopontin-NF-kappa B Signaling Pathway. Theranostics. 2018;8(4):921–40. 10.7150/thno.22182 10.7150/thno.22182 PMC5817102 29463991

[35] Bae JY, Kim EK, Yang DH, Zhang X, Park YJ, Lee DY et al. Reciprocal interaction between carcinoma-associated fibroblasts and squamous carcinoma cells through interleukin-1alpha induces cancer progression. Neoplasia. 2014;16(11):928–38. 10.1016/j.neo.2014.09.003 10.1016/j.neo.2014.09.003 PMC4240921 25425967

[36] Rhim AD, Oberstein PE, Thomas DH, Mirek ET, Palermo CF, Sastra SA et al. Stromal elements act to restrain, rather than support, pancreatic ductal adenocarcinoma. Cancer Cell. 2014;25(6):735–47. 10.1016/j.ccr.2014.04.021 10.1016/j.ccr.2014.04.021 PMC4096698 24856585

[37] Ohlund D, Handly-Santana A, Biffi G, Elyada E, Almeida AS, Ponz-Sarvise M et al. Distinct populations of inflammatory fibroblasts and myofibroblasts in pancreatic cancer. J Exp Med. 2017;214(3):579–96. 10.1084/jem.20162024 10.1084/jem.20162024 PMC5339682 28232471

[38] Elyada E, Bolisetty M, Laise P, Flynn WF, Courtois ET, Burkhart RA et al. Cross-Species Single-Cell Analysis of Pancreatic Ductal Adenocarcinoma Reveals Antigen-Presenting Cancer-Associated Fibroblasts. Cancer Discov. 2019;9(8):1102–23. 10.1158/2159-8290.CD-19-0094 10.1158/2159-8290.CD-19-0094 PMC6727976 31197017

[39] Shintani S, Ishikawa T, Nonaka T, Li C, Nakashiro K, Wong DT et al. Growth-regulated oncogene-1 expression is associated with angiogenesis and lymph node metastasis in human oral cancer. Oncology. 2004;66(4):316–22. 10.1159/000078333 10.1159/000078333 15218300

[40] Reyimu A, Chen Y, Song X, Zhou W, Dai J, Jiang F. Identification of latent biomarkers in connection with progression and prognosis in oral cancer by comprehensive bioinformatics analysis. World J Surg Oncol. 2021;19(1):240. 10.1186/s12957-021-02360-w 10.1186/s12957-021-02360-w PMC8361649 34384424

[41] Yang B, Dong K, Guo P, Guo P, Jie G, Zhang G et al. Identification of Key Biomarkers and Potential Molecular Mechanisms in Oral Squamous Cell Carcinoma by Bioinformatics Analysis. J Comput Biol. 2020;27(1):40–54. 10.1089/cmb.2019.0211 10.1089/cmb.2019.0211 31424263

[42] Sun W, Qiu Z, Huang W, Cao M. Gene expression profiles and protein‑protein interaction networks during tongue carcinogenesis in the tumor microenvironment. Mol Med Rep. 2018;17(1):165–71. 10.3892/mmr.2017.7843 10.3892/mmr.2017.7843 PMC5780107 29115421

[43] Brondum L, Eriksen JG, Singers Sorensen B, Mortensen LS, Toustrup K, Overgaard J et al. Plasma proteins as prognostic biomarkers in radiotherapy treated head and neck cancer patients. Clin Transl Radiat Oncol. 2017;2:46–52.10.1016/j.ctro.2017.01.001 10.1016/j.ctro.2017.01.001 PMC5893530 29658000

[44] Dufies M, Grytsai O, Ronco C, Camara O, Ambrosetti D, Hagege A et al. New CXCR1/CXCR2 inhibitors represent an effective treatment for kidney or head and neck cancers sensitive or refractory to reference treatments. Theranostics. 2019;9(18):5332–46. 10.7150/thno.34681 10.7150/thno.34681 PMC6691587 31410218

[45] Ye MY, Chen MY, Chang YH, Huang JS, Huang TT, Wong TY et al. Growth-regulated oncogene-alpha from oral submucous fibrosis fibroblasts promotes malignant transformation of oral precancerous cells. J Oral Pathol Med. 2018;47(9):880–6.10.1111/jop.12768 10.1111/jop.12768 30035347

[46] Hughes CE, Nibbs RJB. A guide to chemokines and their receptors. FEBS J. 2018;285(16):2944–71. 10.1111/febs.14466 10.1111/febs.14466 PMC6120486 29637711

[47] White JR, Lee JM, Young PR, Hertzberg RP, Jurewicz AJ, Chaikin MA et al. Identification of a potent, selective non-peptide CXCR2 antagonist that inhibits interleukin-8-induced neutrophil migration. J Biol Chem. 1998;273(17):10095–8.10.1074/jbc.273.17.10095 10.1074/jbc.273.17.10095 9553055

[48] Romanini J, Mielcke TR, Leal PC, Figueiredo CP, Calixto JB, Morrone FB et al. The role of CXCR2 chemokine receptors in the oral squamous cell carcinoma. Invest New Drugs. 2012;30(4):1371–8. 10.1007/s10637-011-9701-x 10.1007/s10637-011-9701-x 21670971

[49] Sueoka H, Hirano T, Uda Y, Iimuro Y, Yamanaka J, Fujimoto J. Blockage of CXCR2 suppresses tumor growth of intrahepatic cholangiocellular carcinoma. Surgery. 2014;155(4):640–9. 10.1016/j.surg.2013.12.037 10.1016/j.surg.2013.12.037 24582495

[50] Ning Y, Labonte MJ, Zhang W, Bohanes PO, Gerger A, Yang D et al. The CXCR2 antagonist, SCH-527123, shows antitumor activity and sensitizes cells to oxaliplatin in preclinical colon cancer models. Mol Cancer Ther. 2012;11(6):1353–64. 10.1158/1535-7163.MCT-11-0915 10.1158/1535-7163.MCT-11-0915 22391039

[51] Korbecki J, Bosiacki M, Barczak K, Lagocka R, Chlubek D, Baranowska-Bosiacka I. The Clinical Significance and Role of CXCL1 Chemokine in Gastrointestinal Cancers. Cells. 2023;12(10). 10.3390/cells12101406 10.3390/cells12101406 PMC10217339 37408240

[52] Polizzi A, Santonocito S, Distefano A, De Pasquale R, Alibrandi A, Alanazi AM et al. Analysis of oral lichen planus severity on micro-RNA linked with malignant transformation risks. Oral Dis. 2024;30(5):2918–28.10.1111/odi.14758 10.1111/odi.14758 37837187

[53] Polizzi A, Tartaglia GM, Santonocito S, Alibrandi A, Verzi AE, Isola G. Impact of Topical Fluocinonide on Oral Lichen Planus Evolution: Randomized Controlled Clinical Trial. Oral Dis. 2025;31(2):510–21.10.1111/odi.15156 10.1111/odi.15156 PMC11976136 39402896

[54] Zhang G, Wang Q, Qi X, Yang H, Su X, Yang M et al. OShnscc: a novel user-friendly online survival analysis tool for head and neck squamous cell carcinoma based on RNA expression profiles and long-term survival information. J Zhejiang Univ Sci B. 2022;23(3):249–57. 10.1631/jzus.B2100512 10.1631/jzus.B2100512 PMC8913923 35261220

