# Supplementary material for: C-X-C motif chemokine ligand 1 derived from oral squamous cell carcinoma promotes cancer-associated fibroblast differentiation and tumor growth
Source: Mol Biomed. 2025 Jun 10;6:40. doi: 10.1186/s43556-025-00281-8 (PMC12149066; doi:10.1186/s43556-025-00281-8)
Supplement: Supplementary file 1 — Supplementary Material 1. [file 43556_2025_281_MOESM1_ESM.docx]

**Supplementary Information**

C-X-C motif chemokine ligand 1 derived from oral squamous cell carcinoma promotes cancer-associated fibroblast differentiation and tumor growth

Soon Chul Heo^1,2,†^, In-hye Nam^3†^, Bo Ram Keum^1^, Yeo Gyun Yun^4^, Jae-Yeol Lee^3*^, Hyung Joon Kim^1*^

^1^Department of Oral Physiology, Periodontal Diseases Signaling Network Research Center, Dental and Life Science Institute, School of Dentistry, Pusan National University, Yangsan 50612, Republic of Korea.

^2^Institute of Tissue Regeneration Engineering (ITREN), Mechanobiology Dental Medicine Research Center, Dankook University, Cheonan 31116, Republic of Korea.

^3^Department of Oral and Maxillofacial Surgery, Dental Research Institute, and Dental and Life Science Institute, Pusan National University, School of Dentistry, Yangsan 50612, Republic of Korea.

^4^Institute of Tissue Regeneration Engineering (ITREN), Department of Nanobiomedical Science and BK21 Four NBM Global Research Center for Regenerative Medicine, Dankook University, Cheonan 31116, Republic of Korea.

^†^Soon Chul Heo and In-hye Nam contributed equally to this study.

^*^Corresponding author: Jae-Yeol Lee, omsljy@pusan.ac.kr and Hyung Joon Kim, hjoonkim@pusan.ac.kr


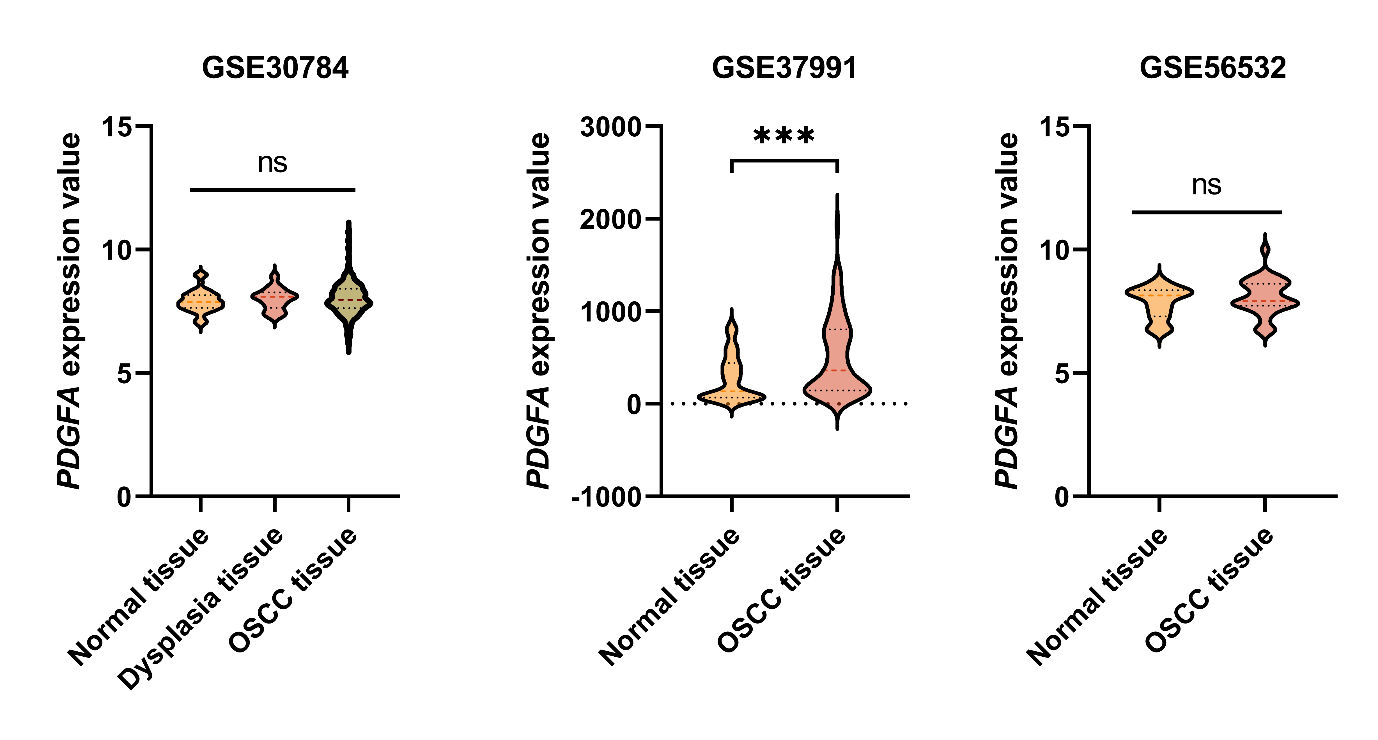


Figure S1. Comparison of *PDGFA* expression between normal individuals and patients with OSCC using data from the GEO database. ****p* < 0.001; ns, not significant


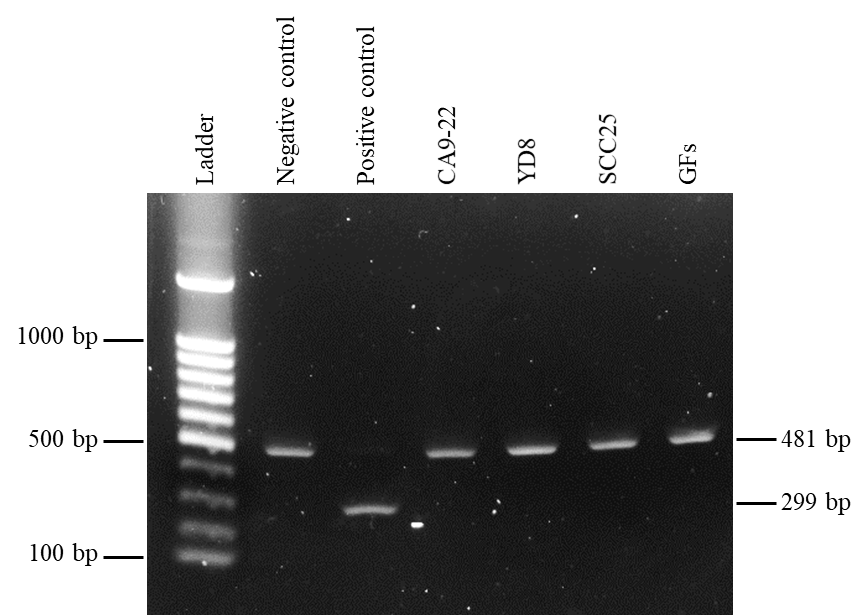


Figure S2. PCR-based detection of mycoplasma contamination in cell culture samples. Mycoplasma contamination was evaluated in four cell culture samples using the Mycoplasma PCR Detection Kit (Sigma, MP0035) following the manufacturer’s instructions. The expected band sizes for the negative control (~481 bp) and positive control (~299 bp) are indicated. All tested samples showed only the negative control-sized band, confirmed the absence of mycoplasma contamination.

Table S1. Secretomic profiling of conditioned media from OSCC cell lines.

| **Gene symbol** | **Normalized data (log2)** | | | **Description** |
| --- | --- | --- | --- | --- |
|  | **CA9-22** | **YD8** | **SCC25** |  |
| CHD7 | 0.877 | 0.457 | 5.635 | Chromodomain-helicase-DNA-binding protein 7 |
| PDGFA | 0.713 | 1.047 | 5.373 | Platelet-derived growth factor subunit A |
| UCHL1 | 2.263 | 0.796 | 6.587 | Ubiquitin carboxyl-terminal hydrolase isozyme L1 |
| PTHLH | 0.577 | 2.283 | 6.096 | Parathyroid hormone-related protein |
| COL17A1 | 3.719 | 0.715 | 6.855 | Collagen alpha-1(XVII) chain |
| CXCL1 | 1.270 | 0.846 | 4.446 | Growth-regulated alpha protein |
| KRT84 | 0.635 | 1.712 | 4.630 | Keratin, type II cuticular Hb4 |
| DLST | 0.956 | 0.357 | 3.477 | Dihydrolipoyllysine-residue succinyltransferase component of 2-oxoglutarate dehydrogenase complex |
| NDRG1 | 1.332 | 0.242 | 3.688 | Protein NDRG1 |
| NAMPT | 0.359 | 0.837 | 3.170 | Nicotinamide phosphoribosyltransferase |
| HMGA2 | 1.326 | 0.355 | 3.584 | High mobility group protein HMGI-C |
| PHPT1 | 0.025 | 1.438 | 3.344 | 14 kDa phosphohistidine phosphatase |
| CTSV | 1.394 | 1.204 | 4.356 | Cathepsin L2 |
| CNPY2 | 0.696 | 1.370 | 3.778 | Protein canopy homolog 2 |
| CBX3 | 1.179 | 1.005 | 3.718 | Chromobox protein homolog 3 |
| CZIB | 1.166 | -0.806 | 1.875 | CXXC motif containing zinc binding protein |
| HEBP1 | 0.144 | 1.151 | 2.646 | Heme-binding protein 1 |
| CTSH | 0.608 | 3.759 | 5.700 | Pro-cathepsin H |
| SNX12 | 0.287 | 0.369 | 1.943 | Sorting nexin-12 |
| PLAU | 1.252 | 2.350 | 4.860 | Urokinase-type plasminogen activator |
| DCTD | 0.284 | 0.871 | 2.281 | Deoxycytidylate deaminase |
| PPIE | 1.253 | 0.038 | 2.357 | Peptidyl-prolyl cis-trans isomerase E |
| RPSA | 1.620 | 1.087 | 3.761 | 40S ribosomal protein SA |
| PLS1 | 0.137 | 1.017 | 2.193 | Plastin-1 |
| PPIL3 | 0.972 | 0.338 | 2.319 | Peptidyl-prolyl cis-trans isomerase-like 3 |
| SRP9 | 1.697 | -0.156 | 2.490 | Signal recognition particle 9 kDa protein |
| DDX42 | 0.931 | 0.944 | 2.822 | ATP-dependent RNA helicase DDX42 |
| GLRX | 0.440 | 0.573 | 1.945 | Glutaredoxin-1 |
| CORO1C | 3.294 | -0.102 | 4.096 | Coronin-1C |
| IGFBP3 | 0.838 | 4.271 | 6.006 | Insulin-like growth factor-binding protein 3 |
| LAMA1 | 0.771 | 1.109 | 2.740 | Laminin subunit alpha-1 |
| FBN2 | 1.201 | 0.518 | 2.572 | Fibrillin-2 |
| UBE2D2 | 1.235 | 0.835 | 2.891 | Ubiquitin-conjugating enzyme E2 D2 |
| CCDC25 | 1.107 | 0.040 | 1.962 | Coiled-coil domain-containing protein 25 |
| DLD | 2.597 | 1.159 | 4.558 | Dihydrolipoyl dehydrogenase, mitochondrial |
| PLIN3 | 1.083 | 0.807 | 2.647 | Perilipin-3 |
| MOB1B | 0.237 | 1.493 | 2.483 | MOB kinase activator 1B |
| TCP1 | 0.929 | 0.462 | 2.116 | T-complex protein 1 subunit alpha |
| PIN4 | 1.667 | 1.155 | 3.515 | Peptidyl-prolyl cis-trans isomerase NIMA-interacting 4 |
| CTSS | 4.617 | 1.051 | 6.321 | Cathepsin S |
| CHMP2B | 1.242 | 0.264 | 2.134 | Charged multivesicular body protein 2b |
| DNAJC8 | 3.305 | 1.089 | 5.004 | DnaJ homolog subfamily C member 8 |
| RACK1 | 3.040 | 0.897 | 4.507 | Receptor of activated protein C kinase 1 |
| TNNC2 | 1.398 | 0.584 | 2.549 | Troponin C, skeletal muscle |
| PGD | 3.954 | 0.220 | 4.741 | 6-phosphogluconate dehydrogenase, decarboxylating |
| KRT85 | 1.325 | 2.009 | 3.897 | Keratin, type II cuticular Hb5 |
| PPP3R1 | 1.004 | 0.151 | 1.710 | Calcineurin subunit B type 1 |
| ALMS1 | 0.791 | 1.578 | 2.882 | Centrosome-associated protein ALMS1 |
| NUDT1 | 1.082 | 1.637 | 3.223 | Oxidized purine nucleoside triphosphate hydrolase |
| PXDN | 1.273 | 2.389 | 4.146 | Peroxidasin homolog |
| PDCD10 | 1.752 | -0.147 | 2.084 | Programmed cell death protein 10 |
| PAXX | 0.891 | -0.021 | 1.348 | Protein PAXX |
| PFAS | 1.537 | 0.604 | 2.614 | Phosphoribosylformylglycinamidine synthase |
| SERPINF1 | 1.241 | 0.364 | 2.055 | Pigment epithelium-derived factor |
| ADSS2 | 1.154 | 0.126 | 1.718 | Adenylosuccinate synthetase isozyme 2 |
| UBE2T | 0.512 | 0.944 | 1.878 | Ubiquitin-conjugating enzyme E2 T |
| CNN3 | 1.289 | 0.561 | 2.257 | Calponin-3 |
| NARS1 | 1.043 | 0.429 | 1.875 | Asparagine--tRNA ligase, cytoplasmic |
| CSPG4 | 1.340 | 2.904 | 4.635 | Chondroitin sulfate proteoglycan 4 |
| UFM1 | 0.406 | 1.348 | 2.135 | Ubiquitin-fold modifier 1 |
| HSPA9 | 1.290 | 1.718 | 3.379 | Stress-70 protein, mitochondrial |
| TMEM132A | 0.763 | 1.484 | 2.615 | Transmembrane protein 132A |
| FAHD1 | 1.233 | 1.308 | 2.879 | Acylpyruvase FAHD1, mitochondrial |
| PAFAH1B1 | 1.993 | 1.322 | 3.645 | Platelet-activating factor acetylhydrolase IB subunit beta |
| ARHGDIB | 0.494 | 1.439 | 2.255 | Rho GDP-dissociation inhibitor 2 |
| FXN | 1.666 | 0.871 | 2.841 | Frataxin, mitochondrial |
| DUT | 4.201 | 0.776 | 5.277 | Deoxyuridine 5'-triphosphate nucleotidohydrolase, mitochondrial |
| ELOB | 0.657 | 1.146 | 2.099 | Elongin-B |
| NRAS | 1.463 | 1.074 | 2.828 | GTPase NRas |
| CCT7 | 0.740 | 1.724 | 2.736 | T-complex protein 1 subunit eta |
| EIF4B | 4.192 | 1.721 | 6.182 | Eukaryotic translation initiation factor 4B |
| PFDN5 | 1.158 | 0.628 | 2.009 | Prefoldin subunit 5 |
| PPP4C | 1.602 | 0.790 | 2.612 | Serine/threonine-protein phosphatase 4 catalytic subunit |
| SNRPB2 | 0.773 | 0.013 | 1.002 | U2 small nuclear ribonucleoprotein B'' |
| TXNL4A | 0.527 | 1.007 | 1.750 | Thioredoxin-like protein 4A |
| PUDP | 1.103 | 0.739 | 2.056 | Pseudouridine-5'-phosphatase |
| NMT1 | 4.537 | 0.900 | 5.645 | Glycylpeptide N-tetradecanoyltransferase 1 |
| UBQLN1 | 1.190 | 1.887 | 3.265 | Ubiquilin-1 |
| PLG | 0.684 | 0.137 | 1.005 | Plasminogen |
| COL2A1 | 1.051 | 0.065 | 1.295 | Isoform 1 of Collagen alpha-1(II) chain |
| DIABLO | 0.889 | 1.259 | 2.322 | Diablo IAP-binding mitochondrial protein |
| EFNB1 | 3.732 | 1.156 | 5.052 | Ephrin-B1 |
| MAGOH | 1.309 | 1.292 | 2.763 | Protein mago nashi homolog |
| DSTN | 1.522 | 1.947 | 3.623 | Destrin |
| DNAJC9 | 0.912 | 0.416 | 1.462 | DnaJ homolog subfamily C member 9 |
| EML4 | 1.437 | 0.963 | 2.500 | Echinoderm microtubule-associated protein-like 4 |
| PTGR1 | 0.561 | 1.151 | 1.811 | Prostaglandin reductase 1 |
| PBDC1 | 0.709 | 1.292 | 2.090 | Protein PBDC1 |
| KRT31 | 1.902 | 1.426 | 3.411 | Keratin, type I cuticular Ha1 |
| U2AF2 | 1.074 | 1.095 | 2.249 | Splicing factor U2AF 65 kDa subunit |
| RGS10 | 1.579 | 0.932 | 2.571 | Regulator of G-protein signaling 10 |
| NAA25 | 1.019 | 0.746 | 1.822 | N-alpha-acetyltransferase 25, NatB auxiliary subunit |
| YTHDF3 | 1.334 | 0.419 | 1.806 | YTH domain-containing family protein 3 |
| EIF4A1 | 3.769 | 0.315 | 4.118 | Eukaryotic initiation factor 4A-I |
| LCN2 | 6.020 | 2.178 | 8.231 | Neutrophil gelatinase-associated lipocalin |
| YBX1 | 2.922 | 1.249 | 4.198 | Y-box-binding protein 1 |
| LSM4 | 1.842 | 0.910 | 2.773 | U6 snRNA-associated Sm-like protein LSm4 |
| ELOC | 1.357 | 0.948 | 2.314 | Elongin-C |
| MTPN | 2.438 | 1.904 | 4.338 | Myotrophin |
| TMTC3 | 0.396 | 1.096 | 1.463 | Protein O-mannosyl-transferase TMTC3 |
| VEGFC | 3.955 | 1.742 | 5.655 | Vascular endothelial growth factor C |
| UFC1 | 0.595 | 1.143 | 1.694 | Ubiquitin-fold modifier-conjugating enzyme 1 |
| F11R | 4.484 | 0.143 | 4.580 | Junctional adhesion molecule A |
| PPA1 | 1.483 | 0.706 | 2.127 | Inorganic pyrophosphatase |
| CDH13 | 3.103 | 1.030 | 4.066 | Cadherin-13 |
| ACYP1 | 1.138 | 0.844 | 1.902 | Acylphosphatase-1 |
| CS | 1.389 | 1.328 | 2.624 | Citrate synthase, mitochondrial |
| EPRS1 | 2.825 | 1.483 | 4.212 | Bifunctional glutamate/proline--tRNA ligase |
| HNRNPL | 0.549 | 0.550 | 0.999 | Heterogeneous nuclear ribonucleoprotein L |
| UROD | 0.747 | 1.064 | 1.706 | Uroporphyrinogen decarboxylase |
| HNRNPD | 3.497 | 0.786 | 4.174 | Heterogeneous nuclear ribonucleoprotein D0 |
| CPVL | 1.212 | 0.520 | 1.598 | Probable serine carboxypeptidase CPVL |
| ARPC4 | 0.745 | 1.019 | 1.621 | Actin-related protein 2/3 complex subunit 4 |
| SRSF2 | 3.063 | 1.179 | 4.095 | Serine/arginine-rich splicing factor 2 |
| PCNA | 3.118 | 1.434 | 4.403 | Proliferating cell nuclear antigen |
| CHORDC1 | 0.594 | 1.688 | 2.128 | Cysteine and histidine-rich domain-containing protein 1 |
| GC | 0.743 | 0.995 | 1.576 | Vitamin D-binding protein |
| NUDC | 4.531 | 0.560 | 4.905 | Nuclear migration protein nudC |
| NUMA1 | 1.896 | 1.007 | 2.716 | Nuclear mitotic apparatus protein 1 |
| DDX39B | 3.802 | 1.116 | 4.718 | Spliceosome RNA helicase DDX39B |
| CAPRIN1 | 4.685 | 0.473 | 4.956 | Caprin-1 |
| AKR1C3 | 3.818 | 1.502 | 5.110 | Aldo-keto reductase family 1 member C3 |
| PACSIN2 | 1.115 | 1.443 | 2.331 | Protein kinase C and casein kinase substrate in neurons protein 2 |
| AAMP | 0.773 | 1.417 | 1.959 | Angio-associated migratory cell protein |
| TIPRL | 0.234 | 1.881 | 1.849 | TIP41-like protein |
| RPIA | 2.177 | 0.710 | 2.617 | Ribose-5-phosphate isomerase |
| DNAJB6 | 1.158 | 1.310 | 2.197 | DnaJ homolog subfamily B member 6 |
| FHL1 | 1.169 | 0.855 | 1.752 | Four and a half LIM domains protein 1 |
| ELAVL1 | 1.456 | 1.712 | 2.889 | ELAV-like protein 1 |
| CAPG | 2.524 | 1.739 | 3.976 | Macrophage-capping protein |
| CYRIB | 1.329 | 1.396 | 2.434 | CYFIP-related Rac1 interactor B |
| SERPINI1 | 1.753 | 1.096 | 2.558 | Neuroserpin |
| MMP3 | 1.723 | 1.303 | 2.719 | Stromelysin-1 |
| VAT1 | 0.572 | 1.537 | 1.798 | Synaptic vesicle membrane protein VAT-1 homolog |
| MESD | 1.198 | 1.629 | 2.511 | LRP chaperone MESD |
| SF3A1 | 4.150 | 1.194 | 5.025 | Splicing factor 3A subunit 1 |
| CD248 | 0.458 | 1.821 | 1.960 | Endosialin |
| PPP2R1A | 0.946 | 0.808 | 1.426 | Serine/threonine-protein phosphatase 2A 65 kDa regulatory subunit A alpha isoform |
| MFAP2 | 0.559 | 4.237 | 4.462 | Microfibrillar-associated protein 2 |
| GOLGA3 | 1.298 | 0.434 | 1.394 | Golgin subfamily A member 3 |
| PPP5C | 0.626 | 1.421 | 1.703 | Serine/threonine-protein phosphatase 5 |
| UBFD1 | 1.513 | 1.273 | 2.439 | Ubiquitin domain-containing protein UBFD1 |
| GNPNAT1 | 1.266 | 1.640 | 2.558 | Glucosamine 6-phosphate N-acetyltransferase |
| LTBP3 | 0.694 | 2.032 | 2.367 | Latent-transforming growth factor beta-binding protein 3 |
| CNBP | 3.217 | 1.411 | 4.269 | CCHC-type zinc finger nucleic acid binding protein |
| PTK2 | 0.978 | 1.231 | 1.840 | Focal adhesion kinase 1 |
| PTBP1 | 3.679 | 0.703 | 4.012 | Polypyrimidine tract-binding protein 1 |
| OTUB1 | 1.228 | 0.883 | 1.737 | Ubiquitin thioesterase OTUB1 |
| C10orf67 | 0.541 | 1.537 | 1.696 | Uncharacterized protein C10orf67, mitochondrial |
| FKBP4 | 3.958 | 0.721 | 4.288 | Peptidyl-prolyl cis-trans isomerase FKBP4 |
| COPZ1 | 1.482 | 1.109 | 2.173 | Coatomer subunit zeta-1 |
| SERPINA3 | 2.095 | 1.155 | 2.823 | Alpha-1-antichymotrypsin |
| BSG | 6.256 | 0.518 | 6.346 | Basigin |
| SF3B3 | 1.265 | 0.598 | 1.423 | Splicing factor 3B subunit 3 |
| NPEPL1 | 1.369 | 1.293 | 2.216 | Probable aminopeptidase NPEPL1 |
| MTAP | 4.035 | 1.304 | 4.892 | S-methyl-5'-thioadenosine phosphorylase |
| NFATC4 | 1.422 | 1.488 | 2.463 | Nuclear factor of activated T-cells, cytoplasmic 4 |
| VPS26A | 2.990 | 0.685 | 3.223 | Vacuolar protein sorting-associated protein 26A |
| MET | 2.288 | 1.253 | 3.079 | Hepatocyte growth factor receptor |
| RPRD1B | 1.804 | 1.158 | 2.497 | Regulation of nuclear pre-mRNA domain-containing protein 1B |
| GLRX5 | 1.306 | 1.173 | 2.013 | Glutaredoxin-related protein 5, mitochondrial |
| SOD2 | 4.157 | 2.368 | 6.052 | Superoxide dismutase [Mn], mitochondrial |
| MIEN1 | 0.609 | 1.581 | 1.717 | Migration and invasion enhancer 1 |
| ZYX | 1.276 | 1.219 | 2.020 | Zyxin |
| CHI3L1 | 1.047 | 1.553 | 2.122 | Chitinase-3-like protein 1 |
| ITGB4 | 3.457 | 0.391 | 3.371 | Integrin beta-4 |
| KHSRP | 1.245 | 2.091 | 2.846 | Far upstream element-binding protein 2 |
| TTN | 1.301 | 1.378 | 2.181 | Titin |
| WDR12 | 3.254 | 0.907 | 3.659 | Ribosome biogenesis protein WDR12 |
| CIAPIN1 | 1.332 | 0.924 | 1.743 | Anamorsin |
| ATOX1 | 0.865 | 1.457 | 1.804 | Copper transport protein ATOX1 |
| AK1 | 2.655 | 0.344 | 2.477 | Adenylate kinase isoenzyme 1 |
| PAICS | 1.298 | 1.647 | 2.419 | Phosphoribosylaminoimidazole carboxylase/phosphoribosylaminoimidazole succinocarboxamide synthetase |
| ATIC | 1.228 | 1.584 | 2.283 | Bifunctional purine biosynthesis protein ATIC |
| XP32 | 5.812 | 1.049 | 6.330 | Skin-specific protein 32 |
| SNCG | 1.499 | 0.691 | 1.652 | Gamma-synuclein |
| MCM6 | 1.122 | 0.986 | 1.570 | DNA replication licensing factor MCM6 |
| MED23 | 1.800 | 0.749 | 2.005 | Mediator of RNA polymerase II transcription subunit 23 |
| ADGRG6 | 0.282 | 1.958 | 1.694 | Adhesion G-protein coupled receptor G6 |
| TPD52 | 1.477 | 1.778 | 2.705 | Tumor protein D52 |
| CAPZA2 | 1.358 | 1.995 | 2.798 | F-actin-capping protein subunit alpha-2 |
| BGN | 0.335 | 2.309 | 2.078 | Biglycan |
| NEO1 | 0.891 | 1.275 | 1.599 | Neogenin |
| MANF | 3.084 | 0.540 | 3.049 | Mesencephalic astrocyte-derived neurotrophic factor |
| CCDC138 | 1.004 | 2.157 | 2.577 | Coiled-coil domain-containing protein 138 |
| RAD23A | 5.937 | 0.748 | 6.076 | UV excision repair protein RAD23 homolog A |
| WARS1 | 2.836 | 0.731 | 2.955 | Tryptophan--tRNA ligase, cytoplasmic |
| IMUP | 3.005 | 1.304 | 3.697 | Immortalization up-regulated protein |
| GALNT7 | 1.386 | 0.650 | 1.421 | N-acetylgalactosaminyltransferase 7 |
| TENM2 | 1.149 | 2.035 | 2.566 | Teneurin-2 |
| SCRN2 | 1.286 | 1.338 | 1.990 | Secernin-2 |
| UBE2M | 0.862 | 1.309 | 1.533 | NEDD8-conjugating enzyme Ubc12 |
| SYNCRIP | 1.536 | 2.565 | 3.459 | Isoform 3 of Heterogeneous nuclear ribonucleoprotein Q |
| CAPZA1 | 3.410 | 1.046 | 3.811 | F-actin-capping protein subunit alpha-1 |
| SERPINE2 | 1.527 | 6.030 | 6.904 | Glia-derived nexin |
| ENO1 | 1.646 | 0.602 | 1.586 | Isoform MBP-1 of Alpha-enolase |
| APOH | 1.739 | 2.437 | 3.511 | Beta-2-glycoprotein 1 |
| RWDD1 | 1.164 | 1.219 | 1.717 | RWD domain-containing protein 1 |
| SEC24C | 2.029 | 0.847 | 2.201 | Protein transport protein Sec24C |
| NUDT14 | 1.157 | 1.266 | 1.746 | Uridine diphosphate glucose pyrophosphatase NUDT14 |
| CUTC | 0.751 | 1.574 | 1.644 | Copper homeostasis protein cutC homolog |
| PARP1 | -0.053 | 1.768 | 1.029 | Poly [ADP-ribose] polymerase 1 |
| RSU1 | 2.187 | -0.001 | 1.499 | Ras suppressor protein 1 |
| ABRACL | 0.712 | 1.830 | 1.850 | Costars family protein ABRACL |
| BAG3 | 4.246 | 0.812 | 4.365 | BAG family molecular chaperone regulator 3 |
| LANCL1 | 1.226 | 1.478 | 2.004 | Glutathione S-transferase LANCL1 |
| NIT2 | 1.379 | 1.216 | 1.891 | Omega-amidase NIT2 |
| HNRNPAB | 4.702 | 0.623 | 4.608 | Isoform 2 of Heterogeneous nuclear ribonucleoprotein A/B |
| TRAP1 | 6.259 | 1.252 | 6.793 | Heat shock protein 75 kDa, mitochondrial |
| CA2 | 6.346 | 0.525 | 6.151 | Carbonic anhydrase 2 |
| AK2 | 3.846 | 1.271 | 4.396 | Adenylate kinase 2, mitochondrial |
| ABHD14B | 2.354 | 1.090 | 2.723 | Putative protein-lysine deacylase ABHD14B |
| GALM | 2.216 | 1.003 | 2.498 | Galactose mutarotase |
| UCHL3 | 1.486 | 1.437 | 2.194 | Ubiquitin carboxyl-terminal hydrolase isozyme L3 |
| PGM1 | 1.757 | 1.499 | 2.527 | Phosphoglucomutase-1 |
| PCDH7 | 3.633 | 0.738 | 3.637 | Protocadherin-7 |
| FST | 3.652 | 1.578 | 4.494 | Follistatin |
| DAZAP1 | 1.117 | 1.771 | 2.150 | DAZ-associated protein 1 |
| SKP1 | 3.578 | 1.467 | 4.285 | S-phase kinase-associated protein 1 |
| PCBP2 | 1.313 | 1.064 | 1.613 | Poly(rC)-binding protein 2 |
| NID1 | 1.140 | 1.519 | 1.891 | Nidogen-1 |
| CAPNS1 | 3.476 | 0.557 | 3.262 | Calpain small subunit 1 |
| PYGL | 1.135 | 2.020 | 2.383 | Glycogen phosphorylase, liver form |
| C9 | 1.201 | 1.824 | 2.249 | Complement component C9 |
| UBR7 | 0.020 | 1.438 | 0.668 | Putative E3 ubiquitin-protein ligase UBR7 |
| PLCB3 | 0.506 | 1.675 | 1.389 | 1-phosphatidylinositol 4,5-bisphosphate phosphodiesterase beta-3 |
| MECP2 | 2.220 | 0.892 | 2.315 | Methyl-CpG-binding protein 2 |
| ADPRS | 1.923 | 0.950 | 2.076 | ADP-ribosylhydrolase ARH3 |
| VAPA | 1.466 | 1.193 | 1.859 | Vesicle-associated membrane protein-associated protein A |
| IGFBP2 | 0.698 | 1.514 | 1.408 | Insulin-like growth factor-binding protein 2 |
| MATR3 | 0.927 | 2.175 | 2.296 | Matrin-3 |
| DSC2 | 3.676 | 1.669 | 4.537 | Desmocollin-2 |
| HSPA7 | 1.592 | 1.310 | 2.093 | Putative heat shock 70 kDa protein 7 |
| ISOC1 | 0.897 | 1.702 | 1.789 | Isochorismatase domain-containing protein 1 |
| CACYBP | 1.874 | 1.885 | 2.949 | Calcyclin-binding protein |
| MACROH2A1 | 1.433 | 1.021 | 1.644 | Core histone macro-H2A.1 |
| PITPNB | 1.029 | 1.256 | 1.470 | Phosphatidylinositol transfer protein beta isoform |
| ECI1 | 1.100 | 2.088 | 2.372 | Enoyl-CoA delta isomerase 1, mitochondrial |
| CCT2 | 0.744 | 1.602 | 1.525 | T-complex protein 1 subunit beta |
| DCAF8 | 1.164 | 2.085 | 2.422 | Isoform 2 of DDB1- and CUL4-associated factor 8 |
| VCAN | 1.560 | 0.620 | 1.353 | Versican core protein |
| ITGAE | 1.186 | 2.147 | 2.499 | Integrin alpha-E |
| NUDT21 | 1.339 | 1.745 | 2.247 | Cleavage and polyadenylation specificity factor subunit 5 |
| ANKMY1 | 0.989 | 1.026 | 1.169 | Ankyrin repeat and MYND domain-containing protein 1 |
| NUDT3 | 1.364 | 0.990 | 1.508 | Diphosphoinositol polyphosphate phosphohydrolase 1 |
| CLIC1 | 4.707 | 1.687 | 5.533 | Chloride intracellular channel protein 1 |
| PFDN1 | 0.691 | 2.234 | 2.063 | Prefoldin subunit 1 |
| SSBP1 | 3.515 | 1.417 | 4.066 | Single-stranded DNA-binding protein, mitochondrial |
| HSPB11 | 1.728 | 1.133 | 1.993 | Intraflagellar transport protein 25 homolog |
| TRIP10 | 1.106 | 0.864 | 1.098 | Cdc42-interacting protein 4 |
| RPLP0 | 2.614 | 1.602 | 3.343 | 60S acidic ribosomal protein P0 |
| PCBP1 | 3.154 | 1.717 | 3.998 | Poly(rC)-binding protein 1 |
| CCT8 | 2.541 | 0.552 | 2.206 | T-complex protein 1 subunit theta |
| PTGDS | 1.611 | 1.169 | 1.891 | Prostaglandin-H2 D-isomerase |
| TLN1 | 1.867 | 0.727 | 1.696 | Talin-1 |
| TGM2 | 1.909 | 1.375 | 2.381 | Protein-glutamine gamma-glutamyltransferase 2 |
| ZNF207 | 1.566 | 1.396 | 2.056 | BUB3-interacting and GLEBS motif-containing protein ZNF207 |
| DSC3 | 7.509 | 0.748 | 7.349 | Desmocollin-3 |
| CPOX | 1.766 | 1.206 | 2.062 | Oxygen-dependent coproporphyrinogen-III oxidase, mitochondrial |
| SAFB | 1.934 | 0.814 | 1.838 | Scaffold attachment factor B1 |
| ISG15 | 5.110 | 1.074 | 5.264 | Ubiquitin-like protein ISG15 |
| XRCC5 | 1.943 | 0.767 | 1.788 | X-ray repair cross-complementing protein 5 |
| AFP | 5.564 | 0.838 | 5.479 | Alpha-fetoprotein |
| PCNP | 1.502 | 1.281 | 1.860 | PEST proteolytic signal-containing nuclear protein |
| GATD3B | 1.971 | 1.169 | 2.209 | Putative glutamine amidotransferase-like class 1 domain-containing protein 3B, mitochondrial |
| PPP2CB | 0.661 | 1.600 | 1.329 | Serine/threonine-protein phosphatase 2A catalytic subunit beta isoform |
| EEF1D | 3.710 | 1.388 | 4.165 | Elongation factor 1-delta |
| CRKL | 2.991 | 0.696 | 2.750 | Crk-like protein |
| RNPEP | 1.256 | 1.579 | 1.897 | Aminopeptidase B |
| EPCAM | 2.021 | 1.414 | 2.495 | Epithelial cell adhesion molecule |
| NUCKS1 | 5.649 | 1.929 | 6.633 | Nuclear ubiquitous casein and cyclin-dependent kinase substrate 1 |
| NAP1L1 | 2.256 | 1.050 | 2.359 | Nucleosome assembly protein 1-like 1 |
| PSMD9 | 2.507 | 1.089 | 2.643 | 26S proteasome non-ATPase regulatory subunit 9 |
| FKBP3 | 3.818 | 1.309 | 4.175 | Peptidyl-prolyl cis-trans isomerase FKBP3 |
| ATP1A1 | 2.913 | 0.649 | 2.609 | Sodium/potassium-transporting ATPase subunit alpha-1 |
| HYPK | 1.310 | 0.939 | 1.286 | Huntingtin-interacting protein K |
| NASP | 3.643 | 1.368 | 4.047 | Nuclear autoantigenic sperm protein |
| API5 | 1.131 | 2.419 | 2.586 | Apoptosis inhibitor 5 |
| APIP | 1.491 | 1.230 | 1.756 | Methylthioribulose-1-phosphate dehydratase |
| CLINT1 | 1.362 | 1.477 | 1.869 | Clathrin interactor 1 |
| BCAM | 4.897 | 1.055 | 4.981 | Basal cell adhesion molecule |
| DLL1 | 4.398 | 0.593 | 4.019 | Delta-like protein 1 |
| H2AC8 | 5.368 | 1.591 | 5.982 | Histone H2A type 1-B/E |
| MRPL12 | 0.668 | 1.095 | 0.786 | 39S ribosomal protein L12, mitochondrial |
| UBE2R2 | 0.873 | 2.078 | 1.970 | Ubiquitin-conjugating enzyme E2 R2 |
| SBDS | 0.672 | 1.234 | 0.920 | Ribosome maturation protein SBDS |
| HNRNPC | 4.824 | 2.109 | 5.945 | Heterogeneous nuclear ribonucleoproteins C1/C2 |
| PAM | 0.226 | 1.863 | 1.075 | Peptidyl-glycine alpha-amidating monooxygenase |
| EGFR | 4.179 | 1.322 | 4.486 | Epidermal growth factor receptor |
| TUBB | 1.267 | 1.803 | 2.036 | Tubulin beta chain |
| RBBP9 | 1.175 | 1.974 | 2.112 | Serine hydrolase RBBP9 |
| UBE2H | 1.697 | 2.042 | 2.699 | Ubiquitin-conjugating enzyme E2 H |
| HNRNPH1 | 0.954 | 1.474 | 1.387 | Heterogeneous nuclear ribonucleoprotein H |
| SERPINB5 | 3.315 | 1.016 | 3.290 | Serpin B5 |
| RRM2 | 1.180 | 1.226 | 1.364 | Ribonucleoside-diphosphate reductase subunit M2 |
| CCT3 | 1.600 | 1.599 | 2.152 | T-complex protein 1 subunit gamma |
| EIF2S3 | 1.218 | 1.524 | 1.691 | Eukaryotic translation initiation factor 2 subunit 3 |
| HSPH1 | 3.298 | 1.610 | 3.849 | Heat shock protein 105 kDa |
| COL4A3 | 1.306 | 1.782 | 2.027 | Collagen alpha-3(IV) chain |
| ACBD3 | 1.429 | 1.279 | 1.646 | Golgi resident protein GCP60 |
| EIF1AX | 1.297 | 2.207 | 2.436 | Eukaryotic translation initiation factor 1A, X-chromosomal |
| EIF3I | 1.501 | 1.085 | 1.514 | Eukaryotic translation initiation factor 3 subunit I |
| RPLP1 | 2.140 | 2.019 | 3.083 | 60S acidic ribosomal protein P1 |
| ARPC5 | 1.063 | 1.760 | 1.743 | Actin-related protein 2/3 complex subunit 5 |
| PDCD5 | 2.337 | 1.982 | 3.234 | Programmed cell death protein 5 |
| UBA1 | 1.430 | 1.465 | 1.809 | Ubiquitin-like modifier-activating enzyme 1 |
| CSRP1 | 1.794 | 1.014 | 1.714 | Cysteine and glycine-rich protein 1 |
| CASP3 | 6.448 | 1.626 | 6.978 | Caspase-3 |
| AIMP1 | 3.643 | 1.541 | 4.086 | Aminoacyl tRNA synthase complex-interacting multifunctional protein 1 |
| PRDX3 | 1.911 | 1.654 | 2.463 | Thioredoxin-dependent peroxide reductase, mitochondrial |
| ACLY | 2.054 | 1.124 | 2.076 | ATP-citrate synthase |
| TARS1 | 4.264 | 0.606 | 3.763 | Threonine--tRNA ligase 1, cytoplasmic |
| PNP | 5.303 | 1.432 | 5.621 | Purine nucleoside phosphorylase |
| MMP2 | 2.235 | 3.555 | 4.672 | 72 kDa type IV collagenase |
| MAT2B | 5.117 | 0.938 | 4.937 | Methionine adenosyltransferase 2 subunit beta |
| RNH1 | 1.327 | 1.232 | 1.435 | Ribonuclease inhibitor |
| PCOLCE | 0.660 | 1.669 | 1.203 | Procollagen C-endopeptidase enhancer 1 |
| RAB1A | 2.664 | 1.176 | 2.703 | Ras-related protein Rab-1A |
| HYI | 0.847 | 2.502 | 2.211 | Putative hydroxypyruvate isomerase |
| EEA1 | 1.733 | 0.642 | 1.236 | Early endosome antigen 1 |
| THBS2 | 1.212 | 1.165 | 1.238 | Thrombospondin-2 |
| NUDCD1 | 1.215 | 1.742 | 1.812 | NudC domain-containing protein 1 |
| XRCC6 | 2.415 | 0.598 | 1.863 | X-ray repair cross-complementing protein 6 |
| AKAP12 | 1.629 | 1.203 | 1.681 | A-kinase anchor protein 12 |
| GLUD1 | 1.551 | 1.175 | 1.571 | Glutamate dehydrogenase 1, mitochondrial |
| DEK | 1.263 | 1.002 | 1.106 | Protein DEK |
| NID2 | 2.279 | 1.342 | 2.462 | Nidogen-2 |
| SSB | 3.687 | 0.622 | 3.147 | Lupus La protein |
| AK3 | 0.957 | 1.855 | 1.644 | GTP:AMP phosphotransferase AK3, mitochondrial |
| SUMO3 | 1.947 | 1.235 | 2.014 | Small ubiquitin-related modifier 3 |
| USP17L3 | 0.747 | 0.982 | 0.559 | Ubiquitin carboxyl-terminal hydrolase 17-like protein 3 |
| PYCARD | 1.242 | 1.122 | 1.177 | Apoptosis-associated speck-like protein containing a CARD |
| SERBP1 | 3.878 | 1.771 | 4.461 | Plasminogen activator inhibitor 1 RNA-binding protein |
| GLRX3 | 2.552 | 1.529 | 2.891 | Glutaredoxin-3 |
| INHBA | 4.577 | 1.466 | 4.849 | Inhibin beta A chain |
| CAND1 | 2.902 | 0.075 | 1.781 | Cullin-associated NEDD8-dissociated protein 1 |
| SLK | 2.091 | 1.310 | 2.193 | STE20-like serine/threonine-protein kinase |
| CFH | 3.958 | 1.733 | 4.481 | Complement factor H |
| IWS1 | 1.051 | 1.453 | 1.288 | Protein IWS1 homolog |
| EIF3CL | 1.798 | 1.834 | 2.412 | Eukaryotic translation initiation factor 3 subunit C-like protein |
| CAB39 | 1.146 | 1.952 | 1.877 | Calcium-binding protein 39 |
| GBE1 | 1.226 | 1.325 | 1.330 | 1,4-alpha-glucan-branching enzyme |
| EFHD2 | 3.922 | 1.745 | 4.445 | EF-hand domain-containing protein D2 |
| TBL1XR1 | 1.462 | 1.770 | 2.007 | F-box-like/WD repeat-containing protein TBL1XR1 |
| FDPS | 4.378 | 1.094 | 4.243 | Farnesyl pyrophosphate synthase |
| SMARCC2 | 2.051 | 1.391 | 2.210 | SWI/SNF complex subunit SMARCC2 |
| TXNL1 | 1.764 | 1.153 | 1.684 | Thioredoxin-like protein 1 |
| HAT1 | 1.419 | 1.352 | 1.536 | Histone acetyltransferase type B catalytic subunit |
| MYH10 | 3.020 | 0.998 | 2.773 | Myosin-10 |
| TSNAX | 1.947 | 1.531 | 2.230 | Translin-associated protein X |
| PAIP1 | 0.862 | 1.967 | 1.577 | Polyadenylate-binding protein-interacting protein 1 |
| DPH3 | 1.690 | 1.743 | 2.175 | Diphthamide biosynthesis protein 3 |
| RPS12 | 3.734 | 0.607 | 3.071 | 40S ribosomal protein S12 |
| SCRN1 | 3.754 | 1.093 | 3.577 | Secernin-1 |
| COPS4 | 1.585 | 1.435 | 1.748 | COP9 signalosome complex subunit 4 |
| FCGR1A | 0.844 | 2.357 | 1.928 | High affinity immunoglobulin gamma Fc receptor I |
| CRABP2 | 1.496 | 1.543 | 1.765 | Cellular retinoic acid-binding protein 2 |
| ACO1 | 1.602 | 0.370 | 0.688 | Cytoplasmic aconitate hydratase |
| GCSH | 2.679 | 0.582 | 1.965 | Glycine cleavage system H protein, mitochondrial |
| AP1B1 | 1.029 | 1.860 | 1.578 | Isoform C of AP-1 complex subunit beta-1 |
| SH3KBP1 | 2.183 | 1.076 | 1.944 | SH3 domain-containing kinase-binding protein 1 |
| LRRFIP1 | 3.484 | 1.682 | 3.830 | Leucine-rich repeat flightless-interacting protein 1 |
| POFUT1 | 1.655 | 1.280 | 1.587 | GDP-fucose protein O-fucosyltransferase 1 |
| TSN | 2.829 | 1.455 | 2.927 | Translin |
| CCT6A | 0.946 | 1.789 | 1.373 | T-complex protein 1 subunit zeta |
| DCTN2 | 1.158 | 1.771 | 1.563 | Dynactin subunit 2 |
| PFN3 | 1.071 | 1.720 | 1.421 | Profilin-3 |
| GRPEL1 | 1.053 | 2.443 | 2.124 | GrpE protein homolog 1, mitochondrial |
| DNPEP | 1.632 | 1.937 | 2.193 | Aspartyl aminopeptidase |
| STX7 | 2.056 | 0.325 | 1.005 | Syntaxin-7 |
| NANS | 1.609 | 1.052 | 1.281 | Sialic acid synthase |
| IPO5 | 2.406 | 0.733 | 1.756 | Importin-5 |
| BLVRA | 1.849 | 1.520 | 1.985 | Biliverdin reductase A |
| YIPF3 | 6.769 | 1.591 | 6.974 | Protein YIPF3 |
| YWHAH | 3.791 | 2.162 | 4.564 | 14-3-3 protein eta |
| UGP2 | 1.784 | 1.033 | 1.427 | UTP--glucose-1-phosphate uridylyltransferase |
| SPTBN1 | 3.040 | 1.531 | 3.172 | Spectrin beta chain, non-erythrocytic 1 |
| SYAP1 | 1.413 | 2.092 | 2.106 | Synapse-associated protein 1 |
| RIDA | 1.429 | 1.557 | 1.585 | 2-iminobutanoate/2-iminopropanoate deaminase |
| RTN4 | 2.891 | 1.425 | 2.913 | Reticulon-4 |
| NME1 | 3.887 | 0.842 | 3.321 | Nucleoside diphosphate kinase A |
| PNPO | 0.944 | 1.748 | 1.282 | Pyridoxine-5'-phosphate oxidase |
| GMFB | 2.349 | 0.968 | 1.906 | Glia maturation factor beta |
| MRTO4 | 1.444 | 1.376 | 1.405 | mRNA turnover protein 4 homolog |
| ARCN1 | 3.930 | 1.192 | 3.704 | Coatomer subunit delta |
| DCN | 1.375 | 2.383 | 2.331 | Decorin |
| PITHD1 | 1.834 | 1.248 | 1.637 | PITH domain-containing protein 1 |
| HNRNPUL2 | 1.214 | 1.334 | 1.100 | Heterogeneous nuclear ribonucleoprotein U-like protein 2 |
| CD59 | 1.386 | 3.147 | 3.084 | CD59 glycoprotein |
| EDF1 | 4.157 | 1.712 | 4.409 | Endothelial differentiation-related factor 1 |
| NRP1 | 0.885 | 4.601 | 4.023 | Neuropilin-1 |
| INPP1 | 2.028 | 0.981 | 1.542 | Inositol polyphosphate 1-phosphatase |
| EEF1B2 | 3.886 | 1.445 | 3.863 | Elongation factor 1-beta |
| GART | 0.815 | 1.384 | 0.729 | Trifunctional purine biosynthetic protein adenosine-3 |
| OAT | 1.785 | 1.597 | 1.910 | Ornithine aminotransferase, mitochondrial |
| TCEA1 | 1.248 | 1.330 | 1.099 | Transcription elongation factor A protein 1 |
| MMP14 | 5.604 | 1.747 | 5.862 | Matrix metalloproteinase-14 |
| LAMB3 | 5.481 | 1.153 | 5.144 | Laminin subunit beta-3 |
| TPR | 1.707 | 1.514 | 1.720 | Nucleoprotein TPR |
| ALDOC | 4.471 | 0.584 | 3.550 | Fructose-bisphosphate aldolase C |
| HLA-C | 4.367 | 1.859 | 4.717 | HLA class I histocompatibility antigen, C alpha chain |
| DENR | 4.495 | 1.479 | 4.458 | Density-regulated protein |
| MIX23 | 2.202 | 1.183 | 1.860 | Protein MIX23 |
| ALDH9A1 | 1.904 | 2.168 | 2.545 | 4-trimethylaminobutyraldehyde dehydrogenase |
| SRI | 1.214 | 1.977 | 1.664 | Isoform 3 of Sorcin |
| HIKESHI | 2.599 | 1.047 | 2.118 | Protein Hikeshi |
| NAE1 | 1.894 | 1.336 | 1.700 | NEDD8-activating enzyme E1 regulatory subunit |
| PRMT1 | 2.976 | 1.071 | 2.509 | Protein arginine N-methyltransferase 1 |
| CCT6B | 1.503 | 1.932 | 1.895 | T-complex protein 1 subunit zeta-2 |
| PPM1G | 0.825 | 2.223 | 1.502 | Protein phosphatase 1G |
| CMPK1 | 3.646 | 1.168 | 3.263 | UMP-CMP kinase |
| ETF1 | 1.668 | 1.451 | 1.560 | Eukaryotic peptide chain release factor subunit 1 |
| ESYT1 | 2.272 | 1.891 | 2.596 | Extended synaptotagmin-1 |
| BCAT1 | 2.012 | 1.523 | 1.964 | Branched-chain-amino-acid aminotransferase, cytosolic |
| PCMT1 | 3.367 | 1.346 | 3.136 | Protein-L-isoaspartate(D-aspartate) O-methyltransferase |
| PLXNA1 | 2.687 | 0.845 | 1.951 | Plexin-A1 |
| IFI16 | 3.397 | 1.198 | 3.002 | Gamma-interferon-inducible protein 16 |
| BID | 2.136 | 1.293 | 1.833 | BH3-interacting domain death agonist |
| OLA1 | 3.518 | 1.295 | 3.215 | Obg-like ATPase 1 |
| DYNLL2 | 1.063 | 1.612 | 1.074 | Dynein light chain 2, cytoplasmic |
| ITGBL1 | 0.763 | 3.347 | 2.499 | Integrin beta-like protein 1 |
| TCOF1 | 5.268 | 1.431 | 5.073 | Isoform 3 of Treacle protein |
| ERO1A | 1.667 | 2.318 | 2.356 | ERO1-like protein alpha |
| VTN | 2.575 | 1.326 | 2.264 | Vitronectin |
| SNRPA | 2.819 | 1.482 | 2.654 | U1 small nuclear ribonucleoprotein A |
| SSRP1 | 1.255 | 1.778 | 1.379 | FACT complex subunit SSRP1 |
| VPS35 | 1.159 | 1.760 | 1.259 | Vacuolar protein sorting-associated protein 35 |
| PSME1 | 3.934 | 0.580 | 2.854 | Proteasome activator complex subunit 1 |
| GNPDA1 | 4.683 | 0.947 | 3.966 | Glucosamine-6-phosphate isomerase 1 |
| EIF6 | 3.450 | 1.978 | 3.761 | Eukaryotic translation initiation factor 6 |
| SEPTIN9 | 2.157 | 1.695 | 2.184 | Septin-9 |
| CDH1 | 7.568 | 1.464 | 7.358 | Cadherin-1 |
| HDGFL3 | 1.759 | 1.428 | 1.512 | Hepatoma-derived growth factor-related protein 3 |
| UBA2 | 1.782 | 0.984 | 1.092 | SUMO-activating enzyme subunit 2 |
| NACA | 2.255 | 1.279 | 1.858 | Nascent polypeptide-associated complex subunit alpha, muscle-specific form |
| IDH1 | 3.944 | 1.693 | 3.943 | Isocitrate dehydrogenase [NADP] cytoplasmic |
| PSMB10 | 2.813 | 0.473 | 1.587 | Proteasome subunit beta type-10 |
| EEF1G | 4.446 | 1.540 | 4.285 | Elongation factor 1-gamma |
| PLOD1 | 2.382 | 0.406 | 1.085 | Procollagen-lysine,2-oxoglutarate 5-dioxygenase 1 |
| RPL12 | 4.409 | 1.191 | 3.891 | 60S ribosomal protein L12 |
| CUTA | 2.978 | 0.871 | 2.131 | Protein CutA |
| RBBP7 | 1.772 | 1.203 | 1.252 | Histone-binding protein RBBP7 |
| CAPN2 | 2.038 | 1.214 | 1.527 | Calpain-2 catalytic subunit |
| PUF60 | 4.360 | 1.388 | 4.016 | Poly(U)-binding-splicing factor PUF60 |
| PPP1R14B | 0.978 | 2.130 | 1.370 | Protein phosphatase 1 regulatory subunit 14B |
| POLR2H | 1.178 | 2.252 | 1.684 | DNA-directed RNA polymerases I, II, and III subunit RPABC3 |
| TRAF2 | 1.548 | 2.545 | 2.328 | TNF receptor-associated factor 2 |
| LOX | 0.972 | 1.991 | 1.192 | Protein-lysine 6-oxidase |
| TWF2 | 2.571 | 2.103 | 2.896 | Twinfilin-2 |
| OGFR | 2.290 | 0.869 | 1.381 | Opioid growth factor receptor |
| ACTR2 | 1.976 | 1.298 | 1.482 | Actin-related protein 2 |
| ADA | 3.448 | 1.302 | 2.957 | Adenosine deaminase |
| PRDX4 | 2.166 | 1.038 | 1.409 | Peroxiredoxin-4 |
| CSTB | 4.160 | 1.811 | 4.175 | Cystatin-B |
| IGLC2 | 2.510 | 2.414 | 3.124 | Immunoglobulin lambda constant 2 |
| LAMC2 | 6.902 | 1.098 | 6.192 | Laminin subunit gamma-2 |
| CMBL | 1.280 | 2.050 | 1.518 | Carboxymethylenebutenolidase homolog |
| IGF2R | 1.772 | 2.558 | 2.513 | Cation-independent mannose-6-phosphate receptor |
| DDX21 | 2.473 | 2.354 | 3.008 | Nucleolar RNA helicase 2 |
| KARS1 | 3.084 | 0.815 | 2.071 | Lysine--tRNA ligase |
| CRELD2 | 2.264 | 1.772 | 2.193 | Protein disulfide isomerase CRELD2 |
| SH3BGRL | 4.879 | 1.412 | 4.445 | Adapter SH3BGRL |
| PGM2 | 2.644 | 1.823 | 2.620 | Phosphopentomutase |
| LAMA3 | 5.293 | 1.971 | 5.414 | Laminin subunit alpha-3 |
| EIF3G | 4.115 | 2.130 | 4.392 | Eukaryotic translation initiation factor 3 subunit G |
| PABPC1 | 0.672 | 2.321 | 1.138 | Polyadenylate-binding protein 1 |
| ABI3BP | 1.822 | 2.234 | 2.199 | Target of Nesh-SH3 |
| SGTA | 0.759 | 2.617 | 1.519 | Small glutamine-rich tetratricopeptide repeat-containing protein alpha |
| LTBP1 | 1.886 | 2.567 | 2.595 | Latent-transforming growth factor beta-binding protein 1 |
| GANAB | 3.469 | 1.460 | 3.068 | Neutral alpha-glucosidase AB |
| EIF2S1 | 1.761 | 1.976 | 1.875 | Eukaryotic translation initiation factor 2 subunit 1 |
| KTN1 | 2.141 | 1.725 | 2.000 | Kinectin |
| PLOD2 | 0.618 | 3.084 | 1.831 | Procollagen-lysine,2-oxoglutarate 5-dioxygenase 2 |
| MAT2A | 3.660 | 0.677 | 2.466 | S-adenosylmethionine synthase isoform type-2 |
| PRPF19 | 2.308 | 1.405 | 1.841 | Pre-mRNA-processing factor 19 |
| POLR2G | 1.364 | 1.335 | 0.816 | DNA-directed RNA polymerase II subunit RPB7 |
| UBE2L3 | 5.140 | 1.293 | 4.547 | Ubiquitin-conjugating enzyme E2 L3 |
| CFL2 | 2.363 | 1.611 | 2.084 | Cofilin-2 |
| PFDN6 | 1.683 | 1.698 | 1.487 | Prefoldin subunit 6 |
| MTDH | 4.373 | 1.903 | 4.373 | Protein LYRIC |
| CTNNA1 | 0.532 | 2.033 | 0.656 | Catenin alpha-1 |
| REXO2 | 3.020 | 1.192 | 2.296 | Oligoribonuclease, mitochondrial |
| KYNU | 1.700 | 1.728 | 1.507 | Kynureninase |
| CSE1L | 2.189 | 1.549 | 1.815 | Exportin-2 |
| TPP2 | 0.809 | 1.923 | 0.805 | Tripeptidyl-peptidase 2 |
| PFDN4 | 1.873 | 1.135 | 1.079 | Prefoldin subunit 4 |
| SUPT16H | 3.147 | 1.977 | 3.191 | FACT complex subunit SPT16 |
| ESD | 1.467 | 2.000 | 1.533 | S-formylglutathione hydrolase |
| SLC2A1 | 2.300 | 1.662 | 2.019 | Solute carrier family 2, facilitated glucose transporter member 1 |
| ERAP1 | 2.386 | 1.027 | 1.462 | Endoplasmic reticulum aminopeptidase 1 |
| SF3B4 | 1.338 | 2.208 | 1.590 | Splicing factor 3B subunit 4 |
| CD63 | 0.492 | 3.186 | 1.721 | CD63 antigen |
| COX17 | 2.182 | 1.791 | 2.015 | Cytochrome c oxidase copper chaperone |
| KPNB1 | 2.962 | 2.291 | 3.288 | Importin subunit beta-1 |
| LSM2 | 1.490 | 2.655 | 2.174 | U6 snRNA-associated Sm-like protein LSm2 |
| TUBB4A | 0.816 | 3.154 | 1.996 | Tubulin beta-4A chain |
| CALML3 | 7.619 | 1.562 | 7.206 | Calmodulin-like protein 3 |
| MCAM | 0.833 | 4.212 | 3.063 | Cell surface glycoprotein MUC18 |
| SEC13 | 0.904 | 1.766 | 0.684 | Protein SEC13 homolog |
| MIF | 8.388 | 0.550 | 6.949 | Macrophage migration inhibitory factor |
| EWSR1 | 3.147 | 1.634 | 2.773 | RNA-binding protein EWS |
| RBBP4 | 3.682 | 0.651 | 2.313 | Histone-binding protein RBBP4 |
| ERP29 | 2.403 | 0.877 | 1.258 | Endoplasmic reticulum resident protein 29 |
| COA6 | 1.593 | 1.478 | 1.041 | Cytochrome c oxidase assembly factor 6 homolog |
| PPIF | 4.176 | 2.934 | 5.059 | Peptidyl-prolyl cis-trans isomerase F, mitochondrial |
| CLTC | 1.617 | 2.606 | 2.171 | Clathrin heavy chain 1 |
| EFEMP1 | 3.381 | 4.861 | 6.190 | EGF-containing fibulin-like extracellular matrix protein 1 |
| PPP1CB | 1.631 | 2.225 | 1.783 | Serine/threonine-protein phosphatase PP1-beta catalytic subunit |
| LTA4H | 4.089 | 0.640 | 2.650 | Leukotriene A-4 hydrolase |
| SERPINB1 | 3.243 | 1.402 | 2.565 | Leukocyte elastase inhibitor |
| CTTN | 1.575 | 4.977 | 4.471 | Src substrate cortactin |
| NT5DC1 | 1.453 | 1.894 | 1.261 | 5'-nucleotidase domain-containing protein 1 |
| SUPT5H | 1.764 | 2.492 | 2.165 | Transcription elongation factor SPT5 |
| ALYREF | 3.796 | 1.367 | 3.073 | THO complex subunit 4 |
| GSR | 5.351 | 1.827 | 5.087 | Glutathione reductase, mitochondrial |
| ERH | 4.002 | 2.423 | 4.319 | Enhancer of rudimentary homolog |
| FKBP1A | 5.615 | 2.306 | 5.815 | Peptidyl-prolyl cis-trans isomerase FKBP1A |
| IL6 | 2.450 | 1.417 | 1.760 | Interleukin-6 |
| C11orf68 | 2.054 | 1.589 | 1.527 | UPF0696 protein C11orf68 |
| FMC1 | 2.132 | 1.244 | 1.247 | Isoform 2 of Protein FMC1 homolog |
| RBKS | 1.371 | 1.069 | 0.311 | Ribokinase |
| CAPZB | 3.436 | 2.202 | 3.506 | F-actin-capping protein subunit beta |
| SRGN | 1.769 | 1.914 | 1.548 | Serglycin |
| HEBP2 | 2.994 | 2.637 | 3.487 | Heme-binding protein 2 |
| TIMM10 | 1.859 | 2.592 | 2.298 | Mitochondrial import inner membrane translocase subunit Tim10 |
| CRK | 2.491 | 2.387 | 2.723 | Adapter molecule crk |
| CCT5 | 2.090 | 2.057 | 1.991 | T-complex protein 1 subunit epsilon |
| CHCHD4 | 2.114 | 1.392 | 1.345 | Mitochondrial intermembrane space import and assembly protein 40 |
| OLFML2A | 4.079 | 2.946 | 4.852 | Olfactomedin-like protein 2A |
| RHOC | 2.242 | 2.083 | 2.148 | Rho-related GTP-binding protein RhoC |
| EIF2S2 | 2.906 | 0.929 | 1.656 | Eukaryotic translation initiation factor 2 subunit 2 |
| EXT2 | 4.065 | 1.390 | 3.269 | Exostosin-2 |
| SLC9A3R1 | 4.160 | 1.182 | 3.155 | Na(+)/H(+) exchange regulatory cofactor NHE-RF1 |
| TFG | 1.238 | 2.197 | 1.237 | Protein TFG |
| SUMO1 | 4.970 | 3.250 | 6.019 | Small ubiquitin-related modifier 1 |
| ST13 | 2.956 | 1.836 | 2.586 | Hsc70-interacting protein |
| C1QBP | 2.065 | 2.510 | 2.368 | Complement component 1 Q subcomponent-binding protein, mitochondrial |
| GRB2 | 2.447 | 1.273 | 1.511 | Growth factor receptor-bound protein 2 |
| SBSN | 1.663 | 2.326 | 1.777 | Suprabasin |
| ALDH1A3 | 2.215 | 1.804 | 1.802 | Aldehyde dehydrogenase family 1 member A3 |
| SERPINB2 | 1.893 | 2.142 | 1.810 | Plasminogen activator inhibitor 2 |
| MAPK1 | 2.588 | 1.432 | 1.790 | Mitogen-activated protein kinase 1 |
| PSPH | 3.229 | 1.137 | 2.126 | Phosphoserine phosphatase |
| PSMF1 | 1.367 | 2.610 | 1.734 | Proteasome inhibitor PI31 subunit |
| ERP44 | 4.017 | 2.004 | 3.753 | Endoplasmic reticulum resident protein 44 |
| UQCRH | 1.235 | 1.617 | 0.581 | Cytochrome b-c1 complex subunit 6, mitochondrial |
| RPS28 | 4.297 | 3.067 | 5.092 | 40S ribosomal protein S28 |
| IQGAP1 | 3.117 | 2.391 | 3.222 | Ras GTPase-activating-like protein IQGAP1 |
| HYOU1 | 4.323 | 0.635 | 2.666 | Hypoxia up-regulated protein 1 |
| PPIA | 5.419 | 1.904 | 5.007 | Isoform 2 of Peptidyl-prolyl cis-trans isomerase A |
| EIF2A | 2.466 | 1.784 | 1.933 | Eukaryotic translation initiation factor 2A |
| CGB1 | 5.003 | 3.671 | 6.351 | Choriogonadotropin subunit beta variant 1 |
| FUS | 0.723 | 2.766 | 1.161 | RNA-binding protein FUS |
| UPP1 | 1.297 | 2.676 | 1.644 | Uridine phosphorylase 1 |
| RBM12 | 1.995 | 1.956 | 1.621 | RNA-binding protein 12 |
| HMGB1 | 5.001 | 2.045 | 4.710 | High mobility group protein B1 |
| COPG1 | 1.988 | 1.881 | 1.528 | Coatomer subunit gamma-1 |
| PPIL1 | 1.907 | 1.747 | 1.307 | Peptidyl-prolyl cis-trans isomerase-like 1 |
| HBB | 1.432 | 2.138 | 1.221 | Hemoglobin subunit beta |
| HNRNPF | 4.152 | 0.993 | 2.788 | Heterogeneous nuclear ribonucleoprotein F |
| CD55 | 1.912 | 5.158 | 4.698 | Complement decay-accelerating factor |
| PSMB8 | 2.473 | 1.445 | 1.540 | Proteasome subunit beta type-8 |
| FKBP9 | 2.144 | 1.497 | 1.260 | Peptidyl-prolyl cis-trans isomerase FKBP9 |
| SRSF1 | 1.852 | 2.518 | 1.988 | Serine/arginine-rich splicing factor 1 |
| PSMB5 | 4.310 | 3.667 | 5.590 | Proteasome subunit beta type-5 |
| FSCN1 | 6.341 | 4.654 | 8.603 | Fascin |
| PTPA | 2.789 | 0.630 | 1.015 | Serine/threonine-protein phosphatase 2A activator |
| GOT2 | 4.617 | 4.272 | 6.480 | Aspartate aminotransferase, mitochondrial |
| EIF3J | 3.394 | 0.900 | 1.878 | Eukaryotic translation initiation factor 3 subunit J |
| CCT4 | 2.737 | 1.987 | 2.303 | T-complex protein 1 subunit delta |
| HSP90AB4P | 1.679 | 1.539 | 0.779 | Putative heat shock protein HSP 90-beta 4 |
| NOLC1 | 3.143 | 1.865 | 2.568 | Nucleolar and coiled-body phosphoprotein 1 |
| IMPA1 | 2.777 | 1.545 | 1.880 | Inositol monophosphatase 1 |
| MAPRE1 | 4.540 | 1.409 | 3.504 | Microtubule-associated protein RP/EB family member 1 |
| STIP1 | 4.565 | 3.447 | 5.566 | Stress-induced-phosphoprotein 1 |
| PLOD3 | 2.710 | 1.465 | 1.721 | Multifunctional procollagen lysine hydroxylase and glycosyltransferase LH3 |
| PSMA5 | 4.816 | 3.197 | 5.553 | Proteasome subunit alpha type-5 |
| TPD52L2 | 3.913 | 1.920 | 3.370 | Tumor protein D54 |
| C20orf27 | 1.829 | 1.719 | 1.080 | UPF0687 protein C20orf27 |
| SEPHS1 | 1.996 | 2.197 | 1.706 | Selenide, water dikinase 1 |
| ARPC1B | 2.326 | 2.135 | 1.974 | Actin-related protein 2/3 complex subunit 1B |
| ILF3 | 4.873 | 1.735 | 4.119 | Interleukin enhancer-binding factor 3 |
| C4A | 0.913 | 4.011 | 2.428 | Complement C4-A |
| IGFBP7 | 1.180 | 8.466 | 7.150 | Insulin-like growth factor-binding protein 7 |
| TMPO | 4.187 | 1.436 | 3.126 | Lamina-associated polypeptide 2, isoform alpha |
| ACO2 | 1.695 | 2.616 | 1.804 | Aconitate hydratase, mitochondrial |
| ACYP2 | 2.428 | 1.681 | 1.600 | Acylphosphatase-2 |
| TFRC | 5.974 | 3.113 | 6.575 | Transferrin receptor protein 1 |
| HNRNPK | 3.441 | 2.862 | 3.789 | Isoform 3 of Heterogeneous nuclear ribonucleoprotein K |
| PTPRS | 3.470 | 1.385 | 2.323 | Receptor-type tyrosine-protein phosphatase S |
| FGFBP1 | 5.024 | 2.255 | 4.739 | Fibroblast growth factor-binding protein 1 |
| EXOSC2 | 1.989 | 1.558 | 0.994 | Exosome complex component RRP4 |
| PSMB9 | 5.184 | 1.801 | 4.428 | Proteasome subunit beta type-9 |
| ANXA5 | 3.669 | 0.791 | 1.902 | Annexin A5 |
| PSMB3 | 4.899 | 2.802 | 5.138 | Proteasome subunit beta type-3 |
| NME2P1 | 3.476 | 0.730 | 1.630 | Putative nucleoside diphosphate kinase |
| LRG1 | 4.365 | 1.490 | 3.254 | Leucine-rich alpha-2-glycoprotein |
| CBX1 | 2.534 | 2.585 | 2.511 | Chromobox protein homolog 1 |
| VASN | 1.312 | 2.429 | 1.122 | Vasorin |
| PFN2 | 3.184 | 2.065 | 2.617 | Profilin-2 |
| KRT7 | 1.577 | 1.855 | 0.787 | Keratin, type II cytoskeletal 7 |
| ACTL6A | 3.246 | 1.326 | 1.919 | Actin-like protein 6A |
| HTRA1 | 0.883 | 3.604 | 1.831 | Serine protease HTRA1 |
| FH | 2.927 | 1.582 | 1.852 | Fumarate hydratase, mitochondrial |
| TES | 2.105 | 1.846 | 1.295 | Testin |
| AFAP1L2 | 2.017 | 2.088 | 1.448 | Actin filament-associated protein 1-like 2 |
| CANX | 2.220 | 2.862 | 2.421 | Calnexin |
| SPINT1 | 4.849 | 2.609 | 4.790 | Kunitz-type protease inhibitor 1 |
| PFDN2 | 3.237 | 1.558 | 2.121 | Prefoldin subunit 2 |
| QDPR | 2.403 | 2.137 | 1.862 | Dihydropteridine reductase |
| HSPA1L | 1.897 | 1.874 | 1.086 | Heat shock 70 kDa protein 1-like |
| BLVRB | 2.659 | 2.738 | 2.711 | Flavin reductase (NADPH) |
| RPLP2 | 3.254 | 1.387 | 1.942 | 60S acidic ribosomal protein P2 |
| COPB2 | 1.852 | 2.346 | 1.496 | Coatomer subunit beta' |
| S100A16 | 2.823 | 2.176 | 2.287 | Protein S100-A16 |
| LMAN2 | 4.962 | 2.143 | 4.388 | Vesicular integral-membrane protein VIP36 |
| AQR | 5.272 | 1.183 | 3.736 | RNA helicase aquarius |
| CLTB | 3.748 | 2.834 | 3.862 | Isoform Non-brain of Clathrin light chain B |
| PLXNB2 | 3.616 | 1.516 | 2.408 | Plexin-B2 |
| FAT2 | 4.243 | 2.266 | 3.784 | Protocadherin Fat 2 |
| UFD1 | 2.057 | 2.825 | 2.140 | Ubiquitin recognition factor in ER-associated degradation protein 1 |
| SP100 | 2.812 | 1.730 | 1.798 | Nuclear autoantigen Sp-100 |
| CAT | 3.188 | 3.604 | 4.045 | Catalase |
| TWF1 | 1.808 | 2.221 | 1.280 | Twinfilin-1 |
| LAMA4 | 2.343 | 2.678 | 2.271 | Laminin subunit alpha-4 |
| NSFL1C | 3.995 | 3.506 | 4.750 | NSFL1 cofactor p47 |
| PSMA2 | 4.936 | 3.649 | 5.832 | Proteasome subunit alpha type-2 |
| GOT1 | 5.289 | 3.874 | 6.407 | Aspartate aminotransferase, cytoplasmic |
| THOP1 | 3.308 | 1.564 | 2.114 | Thimet oligopeptidase |
| TACSTD2 | 3.966 | 1.308 | 2.514 | Tumor-associated calcium signal transducer 2 |
| ITGA3 | 3.372 | 2.260 | 2.867 | Integrin alpha-3 |
| CRIM1 | 3.139 | 4.353 | 4.724 | Cysteine-rich motor neuron 1 protein |
| LGALS3 | 2.420 | 2.832 | 2.475 | Galectin-3 |
| MAP4 | 1.032 | 3.400 | 1.646 | Microtubule-associated protein 4 |
| PLPBP | 1.874 | 2.139 | 1.210 | Pyridoxal phosphate homeostasis protein |
| RCC2 | 3.920 | 2.498 | 3.614 | Protein RCC2 |
| RAB11A | 4.420 | 1.729 | 3.340 | Ras-related protein Rab-11A |
| GSN | 3.676 | 3.720 | 4.582 | Gelsolin |
| CHMP4B | 1.563 | 2.216 | 0.961 | Charged multivesicular body protein 4b |
| IGHG1 | 4.701 | 4.251 | 6.130 | Immunoglobulin heavy constant gamma 1 |
| PRG4 | 5.227 | 1.122 | 3.506 | Proteoglycan 4 |
| HSPD1 | 4.030 | 3.539 | 4.714 | 60 kDa heat shock protein, mitochondrial |
| FASN | 4.009 | 0.879 | 2.027 | Fatty acid synthase |
| PSMB6 | 4.972 | 4.003 | 6.114 | Proteasome subunit beta type-6 |
| NENF | 3.295 | 3.739 | 4.164 | Neudesin |
| MYOF | 2.351 | 2.422 | 1.899 | Myoferlin |
| VCP | 4.534 | 2.757 | 4.411 | Transitional endoplasmic reticulum ATPase |
| TPM4 | 4.865 | 0.535 | 2.517 | Isoform 2 of Tropomyosin alpha-4 chain |
| GSTO1 | 5.745 | 3.014 | 5.874 | Glutathione S-transferase omega-1 |
| BLMH | 3.612 | 4.557 | 5.278 | Bleomycin hydrolase |
| AIFM1 | 5.566 | 2.465 | 5.124 | Apoptosis-inducing factor 1, mitochondrial |
| PRNP | 4.028 | 3.744 | 4.860 | Major prion protein |
| RBMX | 4.916 | 2.571 | 4.569 | RNA-binding motif protein, X chromosome |
| NEDD8 | 3.055 | 1.192 | 1.329 | NEDD8 |
| G6PD | 3.167 | 2.018 | 2.266 | Glucose-6-phosphate 1-dehydrogenase |
| PSMB7 | 4.935 | 3.533 | 5.547 | Proteasome subunit beta type-7 |
| BCAP31 | 3.901 | 0.947 | 1.925 | B-cell receptor-associated protein 31 |
| PA2G4 | 4.035 | 3.866 | 4.967 | Proliferation-associated protein 2G4 |
| PDLIM5 | 2.322 | 1.891 | 1.279 | PDZ and LIM domain protein 5 |
| TPT1 | 4.558 | 4.202 | 5.822 | Translationally-controlled tumor protein |
| MARCKSL1 | 3.274 | 1.354 | 1.671 | MARCKS-related protein |
| KLK6 | 2.584 | 1.691 | 1.310 | Kallikrein-6 |
| LYZ | 2.110 | 3.887 | 3.026 | Lysozyme C |
| G3BP1 | 1.650 | 2.489 | 1.167 | Ras GTPase-activating protein-binding protein 1 |
| IGFBP4 | 1.292 | 6.999 | 5.301 | Insulin-like growth factor-binding protein 4 |
| B4GALT1 | 3.614 | 1.752 | 2.373 | Beta-1,4-galactosyltransferase 1 |
| WDR5 | 2.522 | 1.733 | 1.256 | WD repeat-containing protein 5 |
| TPM1 | 2.385 | 1.732 | 1.118 | Isoform 5 of Tropomyosin alpha-1 chain |
| DDR1 | 6.176 | 2.280 | 5.455 | Epithelial discoidin domain-containing receptor 1 |
| DTD1 | 2.680 | 2.186 | 1.862 | D-aminoacyl-tRNA deacylase 1 |
| PSME2 | 3.156 | 2.332 | 2.475 | Proteasome activator complex subunit 2 |
| ACTR3 | 3.259 | 1.125 | 1.365 | Actin-related protein 3 |
| FUCA1 | 1.393 | 3.681 | 2.042 | Tissue alpha-L-fucosidase |
| FGD6 | 3.718 | 1.136 | 1.819 | FYVE, RhoGEF and PH domain-containing protein 6 |
| PSMA3 | 4.968 | 3.401 | 5.330 | Proteasome subunit alpha type-3 |
| UBE2K | 1.876 | 2.447 | 1.281 | Ubiquitin-conjugating enzyme E2 K |
| PDAP1 | 3.595 | 0.867 | 1.414 | 28 kDa heat- and acid-stable phosphoprotein |
| YWHAQ | 3.673 | 3.169 | 3.790 | 14-3-3 protein theta |
| EIF4G1 | 4.099 | 2.567 | 3.613 | Eukaryotic translation initiation factor 4 gamma 1 |
| NECTIN1 | 4.648 | 1.639 | 3.219 | Nectin-1 |
| GLOD4 | 4.204 | 4.108 | 5.231 | Isoform 2 of Glyoxalase domain-containing protein 4 |
| TXNDC12 | 3.630 | 3.409 | 3.936 | Thioredoxin domain-containing protein 12 |
| SIPA1 | 3.721 | 3.947 | 4.565 | Signal-induced proliferation-associated protein 1 |
| PDIA4 | 4.837 | 2.730 | 4.458 | Protein disulfide-isomerase A4 |
| TUBA1B | 4.211 | 3.342 | 4.442 | Tubulin alpha-1B chain |
| PTPRF | 3.891 | 2.808 | 3.562 | Receptor-type tyrosine-protein phosphatase F |
| GLO1 | 5.241 | 3.451 | 5.552 | Lactoylglutathione lyase |
| PGP | 3.724 | 1.598 | 2.180 | Glycerol-3-phosphate phosphatase |
| PSMA6 | 4.761 | 3.507 | 5.125 | Proteasome subunit alpha type-6 |
| CAPN1 | 3.194 | 1.692 | 1.739 | Calpain-1 catalytic subunit |
| LUM | 0.666 | 5.650 | 3.169 | Lumican |
| AHSA1 | 3.266 | 1.185 | 1.294 | Activator of 90 kDa heat shock protein ATPase homolog 1 |
| SERPINH1 | 3.289 | 1.825 | 1.954 | Serpin H1 |
| LAMB2 | 2.029 | 3.022 | 1.882 | Laminin subunit beta-2 |
| ATP5F1A | 3.120 | 2.266 | 2.212 | ATP synthase subunit alpha, mitochondrial |
| MANBA | 4.531 | 3.286 | 4.624 | Beta-mannosidase |
| ST14 | 4.689 | 2.367 | 3.842 | Suppressor of tumorigenicity 14 protein |
| TATDN1 | 3.386 | 1.939 | 2.110 | Deoxyribonuclease TATDN1 |
| EFNA1 | 7.399 | 1.175 | 5.345 | Ephrin-A1 |
| MS4A14 | 4.446 | 0.115 | 1.323 | Membrane-spanning 4-domains subfamily A member 14 |
| RANBP1 | 4.302 | 0.400 | 1.462 | Ran-specific GTPase-activating protein |
| ARF3 | 3.528 | 1.973 | 2.253 | ADP-ribosylation factor 3 |
| PSAT1 | 5.780 | 2.936 | 5.460 | Phosphoserine aminotransferase |
| LACTB2 | 3.116 | 1.689 | 1.546 | Endoribonuclease LACTB2 |
| PLAUR | 2.744 | 1.188 | 0.669 | Urokinase plasminogen activator surface receptor |
| HNRNPA1 | 4.556 | 3.430 | 4.722 | Heterogeneous nuclear ribonucleoprotein A1 |
| MYDGF | 2.934 | 1.986 | 1.656 | Myeloid-derived growth factor |
| NUDT5 | 5.039 | 3.433 | 5.204 | ADP-sugar pyrophosphatase |
| ANP32A | 6.090 | 3.353 | 6.171 | Acidic leucine-rich nuclear phosphoprotein 32 family member A |
| PEPD | 5.099 | 4.063 | 5.880 | Xaa-Pro dipeptidase |
| SERPINB6 | 4.130 | 1.171 | 2.012 | Serpin B6 |
| MDK | 5.320 | 3.454 | 5.471 | Midkine |
| ITGB1 | 4.204 | 3.172 | 4.068 | Integrin beta-1 |
| SLC7A5 | 5.374 | 1.683 | 3.739 | Large neutral amino acids transporter small subunit 1 |
| RTRAF | 1.488 | 2.565 | 0.724 | RNA transcription, translation and transport factor protein |
| ARPC2 | 3.616 | 2.327 | 2.605 | Actin-related protein 2/3 complex subunit 2 |
| GGCT | 5.569 | 3.310 | 5.538 | Gamma-glutamylcyclotransferase |
| BPNT1 | 3.007 | 1.623 | 1.288 | 3'(2'),5'-bisphosphate nucleotidase 1 |
| ANP32B | 6.059 | 3.323 | 6.028 | Acidic leucine-rich nuclear phosphoprotein 32 family member B |
| ABCF1 | 4.038 | 2.172 | 2.830 | ATP-binding cassette sub-family F member 1 |
| TNFRSF21 | 3.777 | 4.043 | 4.433 | Tumor necrosis factor receptor superfamily member 21 |
| LMNB1 | 4.414 | 3.489 | 4.503 | Lamin-B1 |
| TFPI | 1.818 | 3.628 | 2.045 | Tissue factor pathway inhibitor |
| NCL | 6.362 | 3.693 | 6.645 | Nucleolin |
| RAN | 3.610 | 3.634 | 3.833 | GTP-binding nuclear protein Ran |
| HSPA4 | 4.338 | 3.579 | 4.503 | Heat shock 70 kDa protein 4 |
| S100A2 | 8.149 | 3.307 | 8.022 | Protein S100-A2 |
| HMGN1 | 5.201 | 4.811 | 6.577 | Non-histone chromosomal protein HMG-14 |
| CST6 | 2.700 | 3.310 | 2.563 | Cystatin-M |
| ADAM9 | 3.178 | 3.700 | 3.411 | Disintegrin and metalloproteinase domain-containing protein 9 |
| RBM8A | 4.241 | 4.066 | 4.838 | RNA-binding protein 8A |
| GARS1 | 3.768 | 3.220 | 3.519 | Glycine--tRNA ligase |
| KRT79 | 2.531 | 2.067 | 1.122 | Keratin, type II cytoskeletal 79 |
| EIF3B | 3.055 | 2.527 | 2.104 | Eukaryotic translation initiation factor 3 subunit B |
| LSM8 | 4.117 | 2.842 | 3.477 | U6 snRNA-associated Sm-like protein LSm8 |
| COPA | 3.272 | 1.209 | 0.997 | Coatomer subunit alpha |
| UBE2V1 | 1.770 | 2.776 | 1.062 | Ubiquitin-conjugating enzyme E2 variant 1 |
| SEMA3C | 4.146 | 1.634 | 2.294 | Semaphorin-3C |
| PSMA4 | 5.794 | 3.723 | 6.030 | Proteasome subunit alpha type-4 |
| HSPE1 | 5.910 | 4.222 | 6.641 | 10 kDa heat shock protein, mitochondrial |
| APEX1 | 5.962 | 4.050 | 6.520 | DNA-(apurinic or apyrimidinic site) endonuclease |
| SPTAN1 | 4.954 | 3.112 | 4.562 | Spectrin alpha chain, non-erythrocytic 1 |
| PRKCSH | 5.759 | 3.320 | 5.568 | Glucosidase 2 subunit beta |
| ITIH2 | 3.630 | 1.565 | 1.675 | Inter-alpha-trypsin inhibitor heavy chain H2 |
| CANT1 | 4.413 | 3.622 | 4.511 | Soluble calcium-activated nucleotidase 1 |
| DCTPP1 | 3.360 | 1.622 | 1.445 | dCTP pyrophosphatase 1 |
| PSMB1 | 5.647 | 4.066 | 6.173 | Proteasome subunit beta type-1 |
| FAH | 4.489 | 1.634 | 2.576 | Fumarylacetoacetase |
| CPPED1 | 4.614 | 3.450 | 4.505 | Serine/threonine-protein phosphatase CPPED1 |
| PGLS | 5.499 | 1.928 | 3.857 | 6-phosphogluconolactonase |
| VBP1 | 1.816 | 2.346 | 0.591 | Prefoldin subunit 3 |
| CAST | 2.739 | 3.106 | 2.272 | Calpastatin |
| PTGFRN | 4.598 | 2.659 | 3.684 | Prostaglandin F2 receptor negative regulator |
| APEH | 5.346 | 3.953 | 5.721 | Acylamino-acid-releasing enzyme |
| GM2A | 4.879 | 3.863 | 5.155 | Ganglioside GM2 activator |
| CAP1 | 4.319 | 3.645 | 4.378 | Adenylyl cyclase-associated protein 1 |
| SET | 6.154 | 3.888 | 6.452 | Isoform 2 of Protein SET |
| ATRN | 3.118 | 5.039 | 4.562 | Attractin |
| PKP1 | 3.798 | 3.910 | 4.113 | Isoform 1 of Plakophilin-1 |
| TGM3 | 3.461 | 4.252 | 4.118 | Protein-glutamine gamma-glutamyltransferase E |
| DCBLD2 | 3.600 | 2.054 | 2.058 | Discoidin, CUB and LCCL domain-containing protein 2 |
| LTBP2 | 1.522 | 4.213 | 2.123 | Latent-transforming growth factor beta-binding protein 2 |
| PCBD1 | 3.642 | 1.126 | 1.144 | Pterin-4-alpha-carbinolamine dehydratase |
| APLP2 | 4.831 | 2.961 | 4.166 | Amyloid beta precursor like protein 2 |
| MMP1 | 5.429 | 2.262 | 4.063 | Interstitial collagenase |
| NBL1 | 3.000 | 6.525 | 5.865 | Neuroblastoma suppressor of tumorigenicity 1 |
| TK2 | 1.519 | 4.175 | 2.027 | Isoform 6 of Thymidine kinase 2, mitochondrial |
| LCP1 | 6.670 | 2.419 | 5.415 | Plastin-2 |
| HMGA1 | 5.194 | 5.124 | 6.643 | High mobility group protein HMG-I/HMG-Y |
| MYH3 | 5.018 | 4.875 | 6.207 | Myosin-3 |
| PSMB4 | 5.081 | 3.855 | 5.241 | Proteasome subunit beta type-4 |
| FKBP2 | 3.543 | 1.590 | 1.430 | Peptidyl-prolyl cis-trans isomerase FKBP2 |
| LYPD3 | 4.378 | 5.324 | 5.997 | Ly6/PLAUR domain-containing protein 3 |
| ACY1 | 2.851 | 2.443 | 1.584 | Aminoacylase-1 |
| SERPINB12 | 3.192 | 3.613 | 3.071 | Serpin B12 |
| UBE2N | 5.502 | 2.102 | 3.864 | Ubiquitin-conjugating enzyme E2 N |
| CARS1 | 2.380 | 2.760 | 1.388 | Cysteine--tRNA ligase, cytoplasmic |
| TNC | 6.334 | 0.852 | 3.404 | Tenascin |
| DSG1 | 4.256 | 4.514 | 4.987 | Desmoglein-1 |
| FLNB | 4.870 | 4.229 | 5.309 | Filamin-B |
| PLS3 | 5.669 | 2.746 | 4.624 | Plastin-3 |
| LMNB2 | 4.719 | 3.603 | 4.523 | Lamin-B2 |
| FLG | 3.887 | 4.641 | 4.728 | Filaggrin |
| COL3A1 | 1.507 | 4.957 | 2.664 | Collagen alpha-1(III) chain |
| ILF2 | 3.952 | 1.178 | 1.328 | Interleukin enhancer-binding factor 2 |
| IGHA1 | 2.129 | 5.219 | 3.546 | Immunoglobulin heavy constant alpha 1 |
| PTX3 | 4.368 | 1.369 | 1.921 | Pentraxin-related protein PTX3 |
| EIF5A | 6.259 | 5.475 | 7.911 | Eukaryotic translation initiation factor 5A-1 |
| TPM2 | 2.780 | 2.874 | 1.821 | Isoform 2 of Tropomyosin beta chain |
| ICAM1 | 2.787 | 4.153 | 3.100 | Intercellular adhesion molecule 1 |
| CRYZ | 3.144 | 1.801 | 1.090 | Quinone oxidoreductase |
| HEG1 | 1.167 | 4.774 | 2.053 | Protein HEG homolog 1 |
| NUTF2 | 5.155 | 0.637 | 1.900 | Nuclear transport factor 2 |
| UBE2V2 | 4.773 | 3.291 | 4.164 | Ubiquitin-conjugating enzyme E2 variant 2 |
| WDR1 | 3.870 | 3.671 | 3.601 | WD repeat-containing protein 1 |
| MMP13 | 7.795 | 1.348 | 5.197 | Collagenase 3 |
| RAC1 | 3.221 | 1.874 | 1.148 | Ras-related C3 botulinum toxin substrate 1 |
| CFL1 | 5.928 | 4.736 | 6.710 | Cofilin-1 |
| TGOLN2 | 4.773 | 4.656 | 5.464 | Trans-Golgi network integral membrane protein 2 |
| FLG2 | 3.687 | 5.029 | 4.749 | Filaggrin-2 |
| GBP1 | 4.075 | 1.239 | 1.346 | Guanylate-binding protein 1 |
| PSMB2 | 5.281 | 4.282 | 5.593 | Proteasome subunit beta type-2 |
| HSP90AB1 | 5.674 | 4.169 | 5.868 | Heat shock protein HSP 90-beta |
| DSG2 | 6.805 | 4.347 | 7.169 | Desmoglein-2 |
| PDIA6 | 4.456 | 0.882 | 1.348 | Protein disulfide-isomerase A6 |
| SPON2 | 0.934 | 5.379 | 2.322 | Spondin-2 |
| HSPB1 | 4.366 | 4.842 | 5.209 | Heat shock protein beta-1 |
| C1S | 5.914 | 5.451 | 7.365 | Complement C1s subcomponent |
| NPEPPS | 4.602 | 3.575 | 4.167 | Puromycin-sensitive aminopeptidase |
| EEF2 | 5.474 | 3.902 | 5.354 | Elongation factor 2 |
| IGSF8 | 3.779 | 2.373 | 2.118 | Immunoglobulin superfamily member 8 |
| JPT1 | 5.083 | 4.395 | 5.424 | Jupiter microtubule associated homolog 1 |
| TWSG1 | 1.660 | 7.078 | 4.683 | Twisted gastrulation protein homolog 1 |
| DDB1 | 5.634 | 4.157 | 5.729 | DNA damage-binding protein 1 |
| STMN1 | 4.040 | 4.538 | 4.515 | Stathmin |
| GLG1 | 3.136 | 1.524 | 0.584 | Golgi apparatus protein 1 |
| CCN3 | 2.040 | 7.180 | 5.128 | CCN family member 3 |
| CDH3 | 5.578 | 3.715 | 5.200 | Cadherin-3 |
| RDX | 4.197 | 2.173 | 2.272 | Radixin |
| RPL7A | 5.429 | 5.025 | 6.347 | 60S ribosomal protein L7a |
| STC2 | 3.479 | 4.610 | 3.973 | Stanniocalcin-2 |
| TPP1 | 4.470 | 4.070 | 4.421 | Tripeptidyl-peptidase 1 |
| C1R | 6.383 | 4.543 | 6.806 | Complement C1r subcomponent |
| JPT2 | 3.442 | 2.118 | 1.433 | Jupiter microtubule associated homolog 2 |
| PCSK9 | 1.404 | 5.132 | 2.405 | Proprotein convertase subtilisin/kexin type 9 |
| KRT80 | 4.279 | 4.658 | 4.791 | Keratin, type II cytoskeletal 80 |
| ARG1 | 4.021 | 4.289 | 4.127 | Arginase-1 |
| ANXA1 | 5.133 | 3.798 | 4.745 | Annexin A1 |
| SFN | 7.721 | 4.157 | 7.657 | 14-3-3 protein sigma |
| HMGN2 | 6.040 | 4.765 | 6.581 | Non-histone chromosomal protein HMG-17 |
| PSMA1 | 6.294 | 4.717 | 6.779 | Proteasome subunit alpha type-1 |
| VEGFA | 4.937 | 4.983 | 5.675 | Isoform L-VEGF165 of Vascular endothelial growth factor A |
| HSP90B1 | 4.764 | 3.179 | 3.683 | Endoplasmin |
| COL7A1 | 5.970 | 3.651 | 5.357 | Collagen alpha-1(VII) chain |
| KRT78 | 4.546 | 4.539 | 4.820 | Keratin, type II cytoskeletal 78 |
| ARHGDIA | 5.037 | 2.664 | 3.436 | Rho GDP-dissociation inhibitor 1 |
| H1-5 | 3.678 | 3.562 | 2.943 | Histone H1.5 |
| GSTP1 | 6.934 | 3.753 | 6.391 | Glutathione S-transferase P |
| TF | 4.934 | 5.156 | 5.778 | Serotransferrin |
| GSDMA | 3.494 | 3.893 | 3.073 | Gasdermin-A |
| CDC37 | 4.575 | 0.909 | 1.167 | Hsp90 co-chaperone Cdc37 |
| TXNDC17 | 6.827 | 5.532 | 8.028 | Thioredoxin domain-containing protein 17 |
| TGM1 | 4.711 | 4.183 | 4.553 | Protein-glutamine gamma-glutamyltransferase K |
| PSMA7 | 6.295 | 4.562 | 6.515 | Proteasome subunit alpha type-7 |
| ARSA | 5.119 | 3.658 | 4.414 | Arylsulfatase A |
| SERPINA1 | 5.177 | 0.861 | 1.674 | Alpha-1-antitrypsin |
| DDT | 5.276 | 4.601 | 5.481 | D-dopachrome decarboxylase |
| MAN2B1 | 3.307 | 1.694 | 0.601 | Lysosomal alpha-mannosidase |
| HMGB2 | 4.766 | 2.761 | 3.110 | High mobility group protein B2 |
| COL6A2 | 1.754 | 3.839 | 1.156 | Collagen alpha-2(VI) chain |
| TINAGL1 | 4.893 | 3.304 | 3.752 | Tubulointerstitial nephritis antigen-like |
| KLK10 | 3.565 | 1.902 | 1.019 | Kallikrein-10 |
| YWHAB | 4.637 | 4.845 | 5.020 | 14-3-3 protein beta/alpha |
| MYL6 | 3.954 | 4.206 | 3.695 | Myosin light polypeptide 6 |
| ENOPH1 | 4.797 | 1.872 | 2.203 | Enolase-phosphatase E1 |
| SLC3A2 | 6.055 | 3.083 | 4.666 | 4F2 cell-surface antigen heavy chain |
| COL4A1 | 3.617 | 4.081 | 3.224 | Collagen alpha-1(IV) chain |
| COL4A2 | 4.548 | 4.150 | 4.219 | Collagen alpha-2(IV) chain |
| KRT77 | 3.897 | 4.902 | 4.303 | Keratin, type II cytoskeletal 1b |
| JUP | 4.889 | 5.007 | 5.397 | Junction plakoglobin |
| PTK7 | 4.556 | 4.301 | 4.344 | Inactive tyrosine-protein kinase 7 |
| HSPA1A | 6.383 | 5.394 | 7.250 | Heat shock 70 kDa protein 1A |
| HNRNPU | 3.663 | 3.095 | 2.229 | Heterogeneous nuclear ribonucleoprotein U |
| GSS | 5.422 | 4.393 | 5.283 | Glutathione synthetase |
| IFI30 | 4.866 | 3.376 | 3.695 | Gamma-interferon-inducible lysosomal thiol reductase |
| PARK7 | 5.964 | 4.800 | 6.213 | Parkinson disease protein 7 |
| TALDO1 | 7.295 | 4.170 | 6.912 | Transaldolase |
| CCDC80 | 1.792 | 4.602 | 1.837 | Coiled-coil domain-containing protein 80 |
| SUMF2 | 4.594 | 2.368 | 2.392 | Inactive C-alpha-formylglycine-generating enzyme 2 |
| HPRT1 | 5.299 | 1.277 | 1.998 | Hypoxanthine-guanine phosphoribosyltransferase |
| SUB1 | 4.204 | 1.363 | 0.986 | Activated RNA polymerase II transcriptional coactivator p15 |
| CYCS | 6.480 | 3.779 | 5.674 | Cytochrome c |
| LDHB | 6.209 | 4.071 | 5.684 | L-lactate dehydrogenase B chain |
| MEGF6 | 2.578 | 3.075 | 1.046 | Multiple epidermal growth factor-like domains protein 6 |
| SPOCK1 | 4.017 | 4.405 | 3.789 | Testican-1 |
| VCL | 5.110 | 3.413 | 3.869 | Vinculin |
| GALNT2 | 4.719 | 3.279 | 3.332 | Polypeptide N-acetylgalactosaminyltransferase 2 |
| MMP10 | 3.989 | 1.879 | 1.198 | Stromelysin-2 |
| SDF4 | 4.843 | 5.855 | 6.020 | 45 kDa calcium-binding protein |
| SDCBP | 3.010 | 3.382 | 1.714 | Syntenin-1 |
| PIK3IP1 | 3.857 | 5.754 | 4.932 | Phosphoinositide-3-kinase-interacting protein 1 |
| RNASET2 | 3.406 | 4.673 | 3.389 | Ribonuclease T2 |
| PLBD2 | 5.256 | 3.985 | 4.537 | Putative phospholipase B-like 2 |
| DSC1 | 4.324 | 4.609 | 4.227 | Desmocollin-1 |
| ACTN2 | 3.837 | 4.700 | 3.828 | Alpha-actinin-2 |
| SERPINC1 | 5.203 | 5.328 | 5.821 | Antithrombin-III |
| AHNAK | 4.739 | 4.506 | 4.530 | Neuroblast differentiation-associated protein AHNAK |
| H1-4 | 3.893 | 4.716 | 3.894 | Histone H1.4 |
| PRSS3 | 3.713 | 4.766 | 3.756 | Trypsin-3 |
| RAD23B | 6.654 | 5.069 | 6.964 | UV excision repair protein RAD23 homolog B |
| TMA7 | 6.534 | 3.936 | 5.704 | Translation machinery-associated protein 7 |
| SIRPA | 4.156 | 3.626 | 3.010 | Tyrosine-protein phosphatase non-receptor type substrate 1 |
| TAGLN2 | 6.449 | 3.987 | 5.662 | Transgelin-2 |
| HDGF | 6.126 | 4.231 | 5.556 | Hepatoma-derived growth factor |
| PI3 | 5.206 | 1.644 | 2.048 | Elafin |
| ULBP2 | 6.485 | 3.871 | 5.525 | UL16-binding protein 2 |
| SFPQ | 5.429 | 1.090 | 1.686 | Splicing factor, proline- and glutamine-rich |
| SDC1 | 8.831 | 5.225 | 9.217 | Syndecan-1 |
| MDH2 | 6.685 | 4.258 | 6.104 | Malate dehydrogenase, mitochondrial |
| LSR | 6.643 | 4.257 | 6.039 | Lipolysis-stimulated lipoprotein receptor |
| AZGP1 | 4.359 | 4.504 | 3.981 | Zinc-alpha-2-glycoprotein |
| HAPLN4 | 2.088 | 4.895 | 2.094 | Hyaluronan and proteoglycan link protein 4 |
| LGMN | 4.521 | 4.611 | 4.232 | Legumain |
| SPINT2 | 5.340 | 4.114 | 4.539 | Kunitz-type protease inhibitor 2 |
| BTD | 3.001 | 5.559 | 3.641 | Biotinidase |
| BASP1 | 6.777 | 4.939 | 6.785 | Brain acid soluble protein 1 |
| CLTA | 2.977 | 3.205 | 1.246 | Clathrin light chain A |
| PRDX2 | 5.862 | 4.177 | 5.103 | Peroxiredoxin-2 |
| ECM1 | 5.388 | 4.781 | 5.211 | Extracellular matrix protein 1 |
| ME1 | 4.420 | 2.887 | 2.339 | NADP-dependent malic enzyme |
| GDI1 | 5.675 | 3.016 | 3.712 | Rab GDP dissociation inhibitor alpha |
| AKR1A1 | 4.068 | 2.816 | 1.869 | Aldo-keto reductase family 1 member A1 |
| TXNDC5 | 5.269 | 3.760 | 4.006 | Thioredoxin domain-containing protein 5 |
| PGK1 | 7.187 | 5.837 | 7.990 | Phosphoglycerate kinase 1 |
| PCDH1 | 4.201 | 1.803 | 0.970 | Protocadherin-1 |
| TNFRSF12A | 5.238 | 4.350 | 4.551 | Tumor necrosis factor receptor superfamily member 12A |
| SPRR1B | 4.493 | 5.512 | 4.947 | Cornifin-B |
| S100A11 | 8.186 | 5.659 | 8.783 | Protein S100-A11 |
| HARS1 | 3.383 | 3.014 | 1.331 | Histidine--tRNA ligase, cytoplasmic |
| CSTA | 5.545 | 4.654 | 5.132 | Cystatin-A |
| PVR | 4.011 | 3.492 | 2.431 | Poliovirus receptor |
| PSG9 | 2.367 | 4.436 | 1.723 | Pregnancy-specific beta-1-glycoprotein 9 |
| NAXE | 6.198 | 4.538 | 5.642 | NAD(P)H-hydrate epimerase |
| FSTL3 | 3.930 | 4.363 | 3.199 | Follistatin-related protein 3 |
| HMGN3 | 4.164 | 2.480 | 1.539 | High mobility group nucleosome-binding domain-containing protein 3 |
| PRDX6 | 3.188 | 3.661 | 1.736 | Peroxiredoxin-6 |
| AHSG | 6.111 | 6.626 | 7.621 | Alpha-2-HS-glycoprotein |
| MYH9 | 5.203 | 4.201 | 4.263 | Myosin-9 |
| CD9 | 5.357 | 4.455 | 4.664 | CD9 antigen |
| FAM3C | 6.952 | 4.954 | 6.746 | Protein FAM3C |
| FN1 | 2.973 | 5.840 | 3.642 | Isoform 1 of Fibronectin |
| HNRNPA2B1 | 4.867 | 4.805 | 4.483 | Heterogeneous nuclear ribonucleoproteins A2/B1 |
| CTSC | 4.922 | 4.380 | 4.111 | Dipeptidyl peptidase 1 |
| HSP90AA1 | 7.130 | 4.836 | 6.758 | Heat shock protein HSP 90-alpha |
| LMNA | 6.799 | 5.610 | 7.193 | Prelamin-A/C |
| MCM3 | 4.540 | 2.312 | 1.627 | DNA replication licensing factor MCM3 |
| S100A13 | 6.524 | 3.350 | 4.633 | Protein S100-A13 |
| WFDC2 | 5.185 | 1.628 | 1.535 | WAP four-disulfide core domain protein 2 |
| CD109 | 4.929 | 3.565 | 3.213 | CD109 antigen |
| PLEC | 5.587 | 5.113 | 5.412 | Plectin |
| DPP3 | 5.995 | 4.369 | 5.060 | Dipeptidyl peptidase 3 |
| IGFBP6 | 6.355 | 7.949 | 8.995 | Insulin-like growth factor-binding protein 6 |
| CASP14 | 4.992 | 5.064 | 4.723 | Caspase-14 |
| UBE2I | 5.010 | 3.310 | 2.988 | SUMO-conjugating enzyme UBC9 |
| TBCA | 6.124 | 5.068 | 5.856 | Tubulin-specific chaperone A |
| PKM | 6.613 | 5.148 | 6.414 | Pyruvate kinase PKM |
| CLU | 5.280 | 4.295 | 4.203 | Clusterin |
| PTMA | 8.182 | 4.676 | 7.482 | Prothymosin alpha |
| CTSA | 4.418 | 5.035 | 4.074 | Lysosomal protective protein |
| A2M | 5.242 | 6.028 | 5.876 | Alpha-2-macroglobulin |
| FBN1 | 1.794 | 5.205 | 1.568 | Fibrillin-1 |
| L1CAM | 2.144 | 6.000 | 2.697 | Neural cell adhesion molecule L1 |
| PGAM1 | 6.112 | 4.639 | 5.277 | Phosphoglycerate mutase 1 |
| DYNC1I2 | 4.164 | 2.459 | 1.140 | Cytoplasmic dynein 1 intermediate chain 2 |
| MSN | 6.999 | 5.736 | 7.249 | Moesin |
| LDHA | 7.270 | 6.156 | 7.933 | L-lactate dehydrogenase A chain |
| LAMB1 | 5.556 | 4.652 | 4.708 | Laminin subunit beta-1 |
| EEF1A1 | 6.094 | 5.812 | 6.406 | Elongation factor 1-alpha 1 |
| COL5A2 | 0.986 | 6.554 | 2.037 | Collagen alpha-2(V) chain |
| HLA-A | 5.202 | 3.684 | 3.375 | HLA class I histocompatibility antigen, A alpha chain |
| KRT18 | 4.947 | 5.586 | 5.021 | Keratin, type I cytoskeletal 18 |
| HEXA | 5.871 | 5.150 | 5.501 | Beta-hexosaminidase subunit alpha |
| HBA2 | 4.417 | 5.552 | 4.409 | Hemoglobin subunit alpha |
| ALCAM | 6.034 | 5.345 | 5.804 | CD166 antigen |
| CALR | 7.579 | 4.644 | 6.643 | Calreticulin |
| PRDX1 | 7.034 | 5.946 | 7.399 | Peroxiredoxin-1 |
| PFN1 | 6.657 | 5.564 | 6.625 | Profilin-1 |
| TIMP2 | 1.619 | 6.859 | 2.838 | Metalloproteinase inhibitor 2 |
| HSPA5 | 7.496 | 4.933 | 6.784 | Endoplasmic reticulum chaperone BiP |
| SERPINB3 | 5.397 | 4.970 | 4.704 | Serpin B3 |
| LDLR | 5.515 | 6.720 | 6.515 | Low-density lipoprotein receptor |
| DBI | 4.853 | 2.670 | 1.793 | Acyl-CoA-binding protein |
| AHCY | 5.517 | 5.231 | 4.995 | Adenosylhomocysteinase |
| LAMP1 | 7.125 | 5.781 | 7.149 | Lysosome-associated membrane glycoprotein 1 |
| SF3B2 | 6.109 | 4.940 | 5.287 | Splicing factor 3B subunit 2 |
| NPM1 | 7.612 | 5.927 | 7.773 | Nucleophosmin |
| MDH1 | 7.006 | 5.040 | 6.268 | Malate dehydrogenase, cytoplasmic |
| LGALS1 | 4.851 | 5.907 | 4.968 | Galectin-1 |
| DNASE2 | 7.085 | 5.818 | 7.100 | Deoxyribonuclease-2-alpha |
| CLSTN1 | 7.395 | 7.013 | 8.601 | Isoform 2 of Calsyntenin-1 |
| CDSN | 3.684 | 3.857 | 1.715 | Corneodesmosin |
| RBP4 | 2.404 | 5.007 | 1.586 | Retinol-binding protein 4 |
| ANXA2 | 6.112 | 5.654 | 5.908 | Annexin A2 |
| COTL1 | 6.295 | 5.718 | 6.148 | Coactosin-like protein |
| FBLN1 | 4.987 | 5.371 | 4.478 | Fibulin-1 |
| FABP5 | 7.299 | 5.509 | 6.890 | Fatty acid-binding protein 5 |
| AKR1B1 | 6.370 | 5.023 | 5.475 | Aldo-keto reductase family 1 member B1 |
| KPRP | 5.135 | 6.005 | 5.216 | Keratinocyte proline-rich protein |
| SUMO2 | 7.768 | 5.500 | 7.335 | Small ubiquitin-related modifier 2 |
| DDAH1 | 5.031 | 2.851 | 1.923 | N(G),N(G)-dimethylarginine dimethylaminohydrolase 1 |
| HRNR | 5.837 | 6.153 | 6.021 | Hornerin |
| MT2A | 5.558 | 4.421 | 3.963 | Metallothionein-2 |
| MATN2 | 3.274 | 4.467 | 1.723 | Matrilin-2 |
| ENO1 | 8.210 | 6.075 | 8.266 | Alpha-enolase |
| YWHAG | 8.336 | 5.457 | 7.752 | 14-3-3 protein gamma |
| ENO3 | 5.101 | 5.599 | 4.641 | Beta-enolase |
| COL6A3 | 4.729 | 4.026 | 2.690 | Collagen alpha-3(VI) chain |
| KRT6B | 6.546 | 5.798 | 6.274 | Keratin, type II cytoskeletal 6B |
| KRT19 | 5.405 | 5.661 | 4.994 | Keratin, type I cytoskeletal 19 |
| MSLN | 1.305 | 6.371 | 1.588 | Isoform 3 of Mesothelin |
| KRT13 | 5.897 | 4.815 | 4.614 | Keratin, type I cytoskeletal 13 |
| VIM | 4.255 | 6.802 | 4.957 | Vimentin |
| QSOX1 | 4.679 | 5.037 | 3.589 | Sulfhydryl oxidase 1 |
| H4C16 | 5.553 | 5.601 | 5.019 | Histone H4 |
| P4HB | 6.485 | 4.760 | 5.101 | Protein disulfide-isomerase |
| ACTN1 | 5.588 | 5.541 | 4.985 | Alpha-actinin-1 |
| ACTB | 6.746 | 6.059 | 6.643 | Actin, cytoplasmic 1 |
| SEMA7A | 5.845 | 5.084 | 4.760 | Semaphorin-7A |
| GDI2 | 7.114 | 4.894 | 5.831 | Rab GDP dissociation inhibitor beta |
| EZR | 5.998 | 6.067 | 5.870 | Ezrin |
| GPC1 | 6.893 | 5.538 | 6.227 | Glypican-1 |
| IGFBP5 | 1.839 | 5.293 | 0.922 | Insulin-like growth factor-binding protein 5 |
| NME2 | 7.105 | 4.573 | 5.459 | Isoform 3 of Nucleoside diphosphate kinase B |
| H3C12 | 5.864 | 5.728 | 5.362 | Histone H3.1 |
| COL18A1 | 4.971 | 3.294 | 2.030 | Collagen alpha-1(XVIII) chain |
| TPM3 | 6.888 | 6.117 | 6.758 | Isoform 6 of Tropomyosin alpha-3 chain |
| DSP | 6.554 | 6.588 | 6.892 | Desmoplakin |
| GNS | 5.499 | 5.577 | 4.823 | N-acetylglucosamine-6-sulfatase |
| LAMA5 | 4.816 | 4.229 | 2.759 | Laminin subunit alpha-5 |
| DKK1 | 5.855 | 6.441 | 6.000 | Dickkopf-related protein 1 |
| MYH8 | 5.736 | 6.225 | 5.615 | Myosin-8 |
| AGRN | 7.139 | 6.057 | 6.842 | Isoform 6 of Agrin |
| YWHAZ | 7.623 | 6.800 | 8.032 | 14-3-3 protein zeta/delta |
| GAPDH | 6.618 | 6.397 | 6.604 | Glyceraldehyde-3-phosphate dehydrogenase |
| PTMS | 6.295 | 4.420 | 4.294 | Parathymosin |
| KRT8 | 6.088 | 6.509 | 6.168 | Keratin, type II cytoskeletal 8 |
| RRBP1 | 8.266 | 6.565 | 8.329 | Ribosome-binding protein 1 |
| HAPLN1 | 1.607 | 6.286 | 1.336 | Hyaluronan and proteoglycan link protein 1 |
| COL6A1 | 4.118 | 4.797 | 2.352 | Collagen alpha-1(VI) chain |
| ACTN4 | 7.123 | 5.559 | 6.109 | Alpha-actinin-4 |
| SH3BGRL3 | 6.866 | 7.334 | 7.624 | SH3 domain-binding glutamic acid-rich-like protein 3 |
| TPM4 | 7.595 | 5.253 | 6.253 | Tropomyosin alpha-4 chain |
| COL12A1 | 4.212 | 5.696 | 3.304 | Collagen alpha-1(XII) chain |
| PRCP | 5.064 | 3.837 | 2.284 | Lysosomal Pro-X carboxypeptidase |
| CST3 | 5.835 | 6.124 | 5.338 | Cystatin-C |
| ALB | 7.658 | 8.017 | 9.042 | Albumin |
| LGALS7B | 6.293 | 5.755 | 5.408 | Galectin-7 |
| TXN | 9.118 | 7.108 | 9.585 | Thioredoxin |
| CFB | 6.787 | 4.279 | 4.416 | Complement factor B |
| CTBS | 5.531 | 4.645 | 3.479 | Di-N-acetylchitobiase |
| PDIA3 | 7.752 | 5.520 | 6.568 | Protein disulfide-isomerase A3 |
| KRT16 | 6.841 | 7.447 | 7.579 | Keratin, type I cytoskeletal 16 |
| YWHAE | 8.240 | 7.291 | 8.808 | 14-3-3 protein epsilon |
| ALDOA | 8.395 | 6.714 | 8.365 | Fructose-bisphosphate aldolase A |
| C3 | 5.403 | 5.782 | 4.436 | Complement C3 |
| MARCKS | 7.088 | 5.227 | 5.563 | Myristoylated alanine-rich C-kinase substrate |
| LTF | 6.468 | 6.801 | 6.512 | Lactotransferrin |
| GPI | 7.999 | 6.615 | 7.776 | Glucose-6-phosphate isomerase |
| TPM1 | 5.556 | 5.997 | 4.713 | Isoform 10 of Tropomyosin alpha-1 chain |
| DKK3 | 6.326 | 7.540 | 7.018 | Dickkopf-related protein 3 |
| FLNA | 7.249 | 5.042 | 5.441 | Filamin-A |
| APP | 6.988 | 6.022 | 6.157 | Amyloid-beta precursor protein |
| HEXB | 6.227 | 6.508 | 5.857 | Beta-hexosaminidase subunit beta |
| MYH4 | 6.731 | 6.961 | 6.755 | Myosin-4 |
| GGH | 7.151 | 6.800 | 6.982 | Gamma-glutamyl hydrolase |
| CTSB | 6.453 | 8.033 | 7.511 | Cathepsin B |
| LAMC1 | 5.621 | 5.423 | 4.060 | Laminin subunit gamma-1 |
| MYL11 | 6.815 | 6.950 | 6.780 | Myosin regulatory light chain 11 |
| DAG1 | 5.787 | 6.525 | 5.301 | Dystroglycan 1 |
| PRSS1 | 7.242 | 7.559 | 7.785 | Serine protease 1 |
| PEBP1 | 8.496 | 6.936 | 8.401 | Phosphatidylethanolamine-binding protein 1 |
| CTSD | 7.106 | 7.037 | 7.062 | Cathepsin D |
| LOXL2 | 5.261 | 6.396 | 4.553 | Lysyl oxidase homolog 2 |
| COL1A2 | 4.914 | 7.718 | 5.520 | Collagen alpha-2(I) chain |
| COL5A1 | 6.484 | 7.175 | 6.530 | Collagen alpha-1(V) chain |
| HSPG2 | 6.229 | 6.445 | 5.461 | Basement membrane-specific heparan sulfate proteoglycan core protein |
| SLPI | 7.617 | 5.061 | 5.459 | Antileukoproteinase |
| NUCB1 | 6.369 | 7.657 | 6.782 | Nucleobindin-1 |
| CALU | 6.320 | 6.522 | 5.575 | Calumenin |
| AXL | 4.090 | 7.236 | 4.052 | Tyrosine-protein kinase receptor UFO |
| MYL1 | 6.172 | 6.643 | 5.536 | Myosin light chain 1/3, skeletal muscle isoform |
| NPC2 | 6.389 | 8.099 | 7.208 | NPC intracellular cholesterol transporter 2 |
| RCN1 | 7.012 | 6.761 | 6.478 | Reticulocalbin-1 |
| TXNRD1 | 6.081 | 5.999 | 4.781 | Thioredoxin reductase 1, cytoplasmic |
| H2BC12 | 7.053 | 7.365 | 7.025 | Histone H2B type 1-K |
| HSPA8 | 8.066 | 7.150 | 7.814 | Heat shock cognate 71 kDa protein |
| SPRR2E | 3.392 | 5.956 | 1.899 | Small proline-rich protein 2E |
| CDV3 | 5.734 | 4.122 | 2.404 | Protein CDV3 homolog |
| CTSL | 6.504 | 6.320 | 5.372 | Procathepsin L |
| PTPRK | 6.674 | 6.403 | 5.621 | Isoform 2 of Receptor-type tyrosine-protein phosphatase kappa |
| FUCA2 | 5.569 | 6.235 | 4.329 | Plasma alpha-L-fucosidase |
| CXCL8 | 6.749 | 1.899 | 1.167 | Interleukin-8 |
| CCN2 | 4.100 | 5.475 | 2.076 | CCN family member 2 |
| S100A7 | 5.029 | 4.195 | 1.646 | Protein S100-A7 |
| COL1A1 | 5.454 | 8.399 | 6.238 | Collagen alpha-1(I) chain |
| PPIA | 9.488 | 7.137 | 8.992 | Peptidyl-prolyl cis-trans isomerase A |
| TGFBI | 7.327 | 6.800 | 6.464 | Transforming growth factor-beta-induced protein ig-h3 |
| MYH7 | 6.921 | 6.853 | 6.107 | Myosin-7 |
| S100A9 | 7.075 | 4.593 | 3.972 | Protein S100-A9 |
| PPIB | 8.830 | 6.630 | 7.565 | Peptidyl-prolyl cis-trans isomerase B |
| KRT17 | 7.934 | 5.624 | 5.583 | Keratin, type I cytoskeletal 17 |
| TPI1 | 9.811 | 8.545 | 10.288 | Triosephosphate isomerase |
| GRN | 7.439 | 8.266 | 7.623 | Progranulin |
| S100A6 | 7.471 | 8.703 | 8.092 | Protein S100-A6 |
| TPM3 | 8.872 | 7.860 | 8.467 | Isoform 5 of Tropomyosin alpha-3 chain |
| GOLM1 | 6.829 | 7.441 | 5.996 | Golgi membrane protein 1 |
| PROCR | 5.035 | 4.473 | 1.178 | Endothelial protein C receptor |
| TKT | 8.773 | 6.655 | 7.021 | Transketolase |
| SOD1 | 8.079 | 7.911 | 7.576 | Superoxide dismutase [Cu-Zn] |
| TIMP1 | 5.281 | 8.925 | 5.758 | Metalloproteinase inhibitor 1 |
| SDC4 | 8.491 | 7.965 | 7.983 | Syndecan-4 |
| CALM1 | 9.549 | 8.117 | 9.094 | Calmodulin-1 |
| DCD | 8.148 | 8.529 | 8.082 | Dermcidin |
| THBS1 | 6.490 | 8.593 | 6.437 | Thrombospondin-1 |
| S100A8 | 7.163 | 5.315 | 3.825 | Protein S100-A8 |
| MYH1 | 8.311 | 8.675 | 8.325 | Myosin-1 |
| PSAP | 8.255 | 8.372 | 7.866 | Prosaposin |
| KRT6A | 9.048 | 8.559 | 8.772 | Keratin, type II cytoskeletal 6A |
| UBB | 9.722 | 8.605 | 9.442 | Polyubiquitin-B |
| KRT14 | 8.978 | 9.059 | 9.069 | Keratin, type I cytoskeletal 14 |
| LAMP2 | 5.544 | 4.279 | 0.841 | Lysosome-associated membrane glycoprotein 2 |
| CSF1 | 4.806 | 5.583 | 1.180 | Macrophage colony-stimulating factor 1 |
| SPARC | 0.628 | 10.511 | 1.895 | SPARC |
| SERPINE1 | 9.437 | 5.479 | 5.622 | Plasminogen activator inhibitor 1 |
| ACTC1 | 8.802 | 9.447 | 8.824 | Actin, alpha cardiac muscle 1 |
| CPA4 | 4.002 | 8.015 | 2.514 | Carboxypeptidase A4 |
| CTSZ | 7.608 | 9.064 | 7.027 | Cathepsin Z |
| KRT5 | 9.560 | 9.721 | 9.626 | Keratin, type II cytoskeletal 5 |
| CD44 | 9.887 | 8.860 | 9.024 | CD44 antigen |
| CDH2 | 4.495 | 6.654 | 1.400 | Cadherin-2 |
| LGALS3BP | 9.045 | 10.089 | 9.230 | Galectin-3-binding protein |
| KRT9 | 10.480 | 10.908 | 11.182 | Keratin, type I cytoskeletal 9 |
| KRT2 | 10.372 | 10.682 | 10.222 | Keratin, type II cytoskeletal 2 epidermal |
| B2M | 10.911 | 11.035 | 10.291 | Beta-2-microglobulin |
| KRT1 | 11.782 | 12.247 | 12.242 | Keratin, type II cytoskeletal 1 |
| FSTL1 | 1.479 | 11.860 | 1.473 | Follistatin-related protein 1 |
| KRT10 | 11.651 | 11.923 | 11.572 | Keratin, type I cytoskeletal 10 |
| CCN1 | 8.733 | 8.061 | 4.342 | CCN family member 1 |
| CALML5 | 7.215 | 7.287 | 1.749 | Calmodulin-like protein 5 |

Table S2. List of antibodies used in this study.

| Antigen | Suppliers | Cat. No. | Application | Dilution |
| --- | --- | --- | --- | --- |
| αSMA | Abcam | Ab124964 | ICC/IHC/IF | 1:200 |
| Vimentin | Cell Signaling Technology | 5741 | ICC/IHC/IF | 1:200 |
| phospho-Smad2/3 | Abclonal | AP1343 | WB | 1:1000 |
| Smad2/3 | Cell Signaling Technology | 3102 | WB | 1:1000 |
| phospho-Akt | Abclonal | AP0637 | WB | 1:1000 |
| Akt | Cell Signaling Technology | 2920 | WB | 1:1000 |
| phospho-p38 | Cell Signaling Technology | 4511 | WB | 1:1000 |
| p38 | Cell Signaling Technology | 9212 | WB | 1:1000 |
| phospho-JNK | Cell Signaling Technology | 4668 | WB | 1:1000 |
| JNK | Cell Signaling Technology | 9252 | WB | 1:1000 |
| phospho-ERK | Cell Signaling Technology | 4370 | WB | 1:1000 |
| ERK | Cell Signaling Technology | 4695 | WB | 1:1000 |
| β-actin | Sigma | A3854 | WB | 1:20000 |
| GFP | Biolegend | 338007 | IF | 1:200 |
| Goat anti-Rabbit IgG, Alexa Fluor 488 | Thermo Fisher Scientific | A-11008 | ICC | 1:1000 |
| Goat anti-Rat IgG, Alexa Fluor 488 | Thermo Fisher Scientific | A-11006 | IF | 1:1000 |
| Goat anti-Rabbit IgG , Alexa Fluor 555 | Thermo Fisher Scientific | A-21428 | ICC/IF | 1:1000 |

Abbreviation: αSMA. Alpha smooth muscle actin; GFP, green fluorescent protein; WB, western blotting; ICC, immunocytochemistry; IHC, immunohistochemistry; IF, immunofluorescence

Table S3. List of primers used in this study.

| Gene | Forward primer (5’→3’) | Reverse primer (5’→3’) |
| --- | --- | --- |
| *ACTA2* | CTATGCCTCTGGACGCACAACT | CAGATCCAGACGCATGATGGCA |
| *VIMENTIN* | AGGCAAAGCAGGAGTCCACTGA | ATCTGGCGTTCCAGGGACTCAT |
| *CXCL1* | TGGCTTAGAACAAAGGGGCTT | GGTAGCCCTTGTTTCCCCC |
| *CXCR1* | TCCTTTTCCGCCAGGCTTACCA | GGCACGATGAAGCCAAAGGTGT |
| *CXCR2* | GGGTACAGTGCTATTCTGCCT | AAATCCTGACTGGGTCGCTG |
| *ACTB* | ACTCTTCCAGCCTTCCTTCC | TGTTGGCGTACAGGTCTTTG |
